# Identifying the active ingredients in payment for performance programmes using system dynamics modelling

**DOI:** 10.1016/j.ssmhs.2024.100040

**Published:** 2025-06

**Authors:** Rachel Cassidy, Agnes Rwashana Semwanga, Peter Binyaruka, Karl Blanchet, Neha S. Singh, John Maiba, Josephine Borghi

**Affiliations:** a Department of Global Health and Development, London School of Hygiene and Tropical Medicine, 15-17 Tavistock Place, London WC1H 9SH, UK; b KPM Center for Public Management, University of Bern, Bern 3012, Switzerland; c Swiss Institute for Translational and Entrepreneurial Medicine, Bern 3010, Switzerland; d Information Systems Department, College of Computing and Information Sciences, Makerere University, P.O. Box 7062, Kampala, Uganda; e Ifakara Health Institute, PO Box 78373, Dar es Salaam 78373, Tanzania; f Geneva Centre of Humanitarian Studies, University of Geneva and the Graduate Institute, Rue Rothschild 22, Genève 1211, Switzerland

**Keywords:** Health systems, System dynamics modelling, Payment for performance, Maternal and child health, Primary care, Evaluation, Tanzania

## Abstract

Payment for performance (P4P) is not a uniform intervention, with programme effect dependent on several interconnected factors. In this study, a system dynamics model was developed to explore the pathways to improved outcomes and how changes in the design, implementation and context of a P4P programme affected maternal and child health (MCH) service delivery outcomes in Tanzania. A previously developed causal loop diagram of the programme effects was used to inform model development, with further data sources (including an impact evaluation of programme, health surveys, stakeholder feedback and relevant literature) used to build the model. A number of pathways were identified to improved services under P4P, with increased availability of drugs underpinning the content of care outcome (intermittent preventative treatment during ANC), which together with increased supervision, enhanced health worker motivation. This in turn increased perceived quality of care at the facility which improved the coverage of services outcome (facility-based deliveries), and with increased outreach, increased awareness of services also boosted demand. Minor delays in payment reduced provider purchasing power for medicines, with severe delays driving erosion of provider trust and motivation for programme participation. Allocating a larger share of funds for facility operations can enhance performance effects, particularly for those services that rely on efficient drug administration. Contextual factors including limited baseline provision of essential medications, lower community awareness of facility services and dispersed/distant populations reduced programme effect. This paper demonstrates the feasibility and the potential of such models to inform the design of effective health system interventions.

## 1 Introduction

Payment for performance (P4P) has been implemented in many low- and middle-income countries (LMICs) to improve the quality and coverage of maternal and child health services (Das et al., 2016). P4P encourages achievement of pre-defined indicators through the provision of incentives to health workers and managers for performance attained (Kovacs, 2020, Mannion and Davies, 2008). A recently updated Cochrane review found positive effects of P4P on certain indicators (such as child mortality, quality of child healthcare, medicine availability) and mixed effects on other indicators (including vaccinations, neonatal mortality, and ante-natal care (ANC) utilisation) in articles which compare P4P to a status quo control group (Diaconu et al., 2021). The conclusions drawn from the review intimate that P4P is not a uniform intervention, with programme effect dependent on several variables, including programme design and context within which the programme is implemented.

P4P is a complex intervention acting to influence a complex system, the health system (Diaconu, 2022, Lipsitz, 2012). The properties of the intervention itself are complex; implementers have autonomy in how they respond to and tailor the intervention to their local context, and require expertise, many individuals and groups (cadres of health worker, informal care providers, managers etc.) and service delivery indicators are targeted, and data reporting and measurements are required for performance evaluation (Skivington et al., 2021). The health system in which the intervention is implemented is also complex, exhibiting dynamic, non-linear, emergent behaviour that changes over time in response to numerous stimuli (Paina and Peters, 2012). The relationship between the intervention and health system give rise to an additional layer of complexity; the mechanisms through which the intervention aims to change health system behaviour, and the context in which the intervention is implemented will influence success or failure (Skivington et al., 2021). Tracing the mechanism for impact in response to an intervention is difficult using conventional methods for evaluation that assume linear cause effect relationships and do not account for complexity within the analysis (Borghi and Chalabi, 2017). Furthermore, implementation of a complex intervention can result in unexpected or paradoxical behaviour with suboptimal outcomes as a result of discounting system complexity (Adam and de Savigny, 2012, Paina and Peters, 2012).

Systems thinking methods can be employed to explore the mechanisms through which complex interventions act to influence complex systems (such as the health system), and to better understand what works in a given context. Systems thinking is an umbrella term used to describe a range of tools that can be used for health systems research (Peters, 2014), where system complexity is retained in the analysis. The choice of systems thinking tool depends on the research question (de Savigny et al., 2017). For example, causal loop diagrams (CLDs) can be used to identify and visualise drivers of health system behaviour and pathways to impact for interventions on key health and system outcomes (Baugh Littlejohns, 2018, Cassidy, 2022, Sahin, 2020). CLDs can be used to identify system bottlenecks, catalytic variables (those that have wide spread impact on the rest of the system and should be carefully considered in the design of interventions) and system levers (variables not currently targeted by an intervention but could be incorporated to maximise impact) (Cassidy, 2021, Rwashana, 2014). If there is interest in investigating how the behaviour of the system changes over time in response to new interventions or changes in context and quantifying the effects of such changes on health system outcomes, quantitative system dynamics modelling (SDM) is required (Pruyt, 2017). SDMs can also explore the effects of potential changes in intervention design on health or service delivery outcomes, to determine how programme effects could be maximised. When developed with a user-friendly interface, SDM can be used as a tool to guide policy and support dialogue between stakeholders and researchers (Semwanga et al., 2016). CLDs can be used to develop SDMs, providing a blueprint of dynamic drivers for behaviour that can inform model structure (Pruyt, 2017).

Use of CLDs and SDMs to explore health system behaviour is on the rise (Cassidy, 2019, Cassidy, 2022, Currie et al., 2018, Darabi and Hosseinichimeh, 2020). To our knowledge, five studies have used CLD to study the effect of P4P on health and service outcomes (Alonge, 2017, Cassidy, 2021, Meker and Barlas, 2015, Renmans et al., 2017, Singh, 2021). However, only two studies also developed a simulation model (SDM) to explore the effect of P4P on health systems, in Afghanistan (Alonge et al., 2017) and Turkey (Meker and Barlas, 2015). In a recent systematic review on application of SDM for health systems research (Cassidy et al., 2019), nine articles described simulation of health system behaviour in LMIC settings. Specific to policy evaluation, and in addition to those already mentioned that model P4P, SDM was used to explore policies that would alleviate delays in care for serious heart events in Brazil (Andrade et al., 2014), policies for optimisation of healthcare waste management in Turkey (Ciplak and Barton, 2012) and Indonesia (Chaerul et al., 2008), and interventions to reduce neonatal mortality in Uganda (Semwanga et al., 2016). Given the resource constraints facing many LMIC, there is urgent need for further use of SDM to study health system reforms, such as P4P, in these settings.

The aim of this study was to develop a SDM to explore the mechanisms for impact within a P4P programme, and examine how changes in programme design, implementation and context affect maternal and child health service delivery outcomes in a LMIC setting, Tanzania. This study uses a previously documented CLD of a P4P programme in Tanzania (Cassidy et al., 2021) to inform the development of a SDM.

## 2 Methods

### 2.1 Study setting

The P4P programme in Pwani region of Tanzania is described in detail elsewhere (Binyaruka, 2015, Borghi, 2021), but a summary is provided here. The programme was introduced by the Ministry of Health and Social Welfare in 2011, with funding from the Norwegian Ministry of Foreign Affairs. The programme aimed to improve the coverage and delivery of maternal and child health services through financial incentives for health providers, district and regional managers based on targets achieved. For health providers, targets were aimed at improving the coverage of services (such as percentage of facility-based deliveries), content of care (such as percentage of women who receive intermittent preventative treatment (IPT) as part of ANC) and data reporting practices (Supplementary File 1). Performance was measured every 6 months. To be eligible for a bonus payment, providers needed to either improve by a specified amount in relation to previous performance or achieve an absolute amount of service coverage. For primary health care providers (health centres and dispensaries), 75 % of the incentive payment was to be split between staff at the facility, with the remaining 25 % to be used to improve facility operations (e.g., purchasing additional medicine where needed). District and regional level managers were responsible for supporting facilities and were also eligible to receive incentives based on the performance of facilities within their district/region (Supplementary File 1).

### 2.2 System dynamics modelling

The development of the quantitative SDM involved the development of a CLD and the adaptation of the CLD into a stock and flow diagram (Pruyt, 2017). Whilst CLD notation consists of variables, arrows with attributed polarity (direction of relationship) and feedback loops (Cassidy et al., 2022), stock and flow diagrams consist of stocks , flows , auxiliary variables  and constants  (Pruyt 2017).

**Fig. 1:**
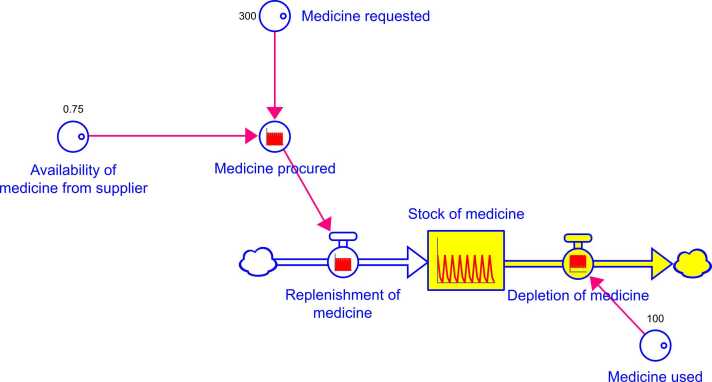
A simple example of a stock and flow diagram.

A simple example of a stock and flow diagram is presented in Fig. 1, demonstrating replenishment and depletion of medicine at a health facility. ‘Stock of medicine’ represents a single stock; a container which changes value over time based on the in and out flows ‘replenishment of medicine’ and ‘depletion of medicine’, respectively. The behaviour of the inflow is dependent on the auxiliary variable ‘medicine procured’, a dynamic variable that changes over time in response to the constant variables ‘availability of medicine from supplier’ and ‘medicine requested’, whose values remain fixed during the simulation. The behaviour of the outflow is dependent on the constant variable ‘medicine used’; in reality, ‘medicine requested’, ‘medicine used’ and ‘availability of medicine from supplier’ are likely to fluctuate over time but for simplicity are given constant variable status here. In this example, a request for medicine is placed every three months (300 items of medicine) but the supplier can only provide 75 % of items requested. Medicine at the health facility is depleting at a steady rate of 100 items per month. The impact is felt on the stock of medicine, which is never fully replenished and often leaves the facility with stockouts.

### 2.3 Model software

The SDM presented in this paper (and in the example above) were developed in STELLA Architect (version 2.1.4) (isee systems inc 2021). STELLA was chosen as the preferred modelling software due to the extensive guidance literature available for model development and functionality that allows users to develop interfaces which can be used to support model testing and discussions with stakeholders.

### 2.4 Development and validation of the SDM

Development and validation of the model can be broadly summarised as following four stages; (i) defining the purpose and goal of the model (ii) creation of model sectors (iii) validation of the model (iv) sensitivity analysis.

#### 2.4.1 Model purpose and data

**Fig. 2:**
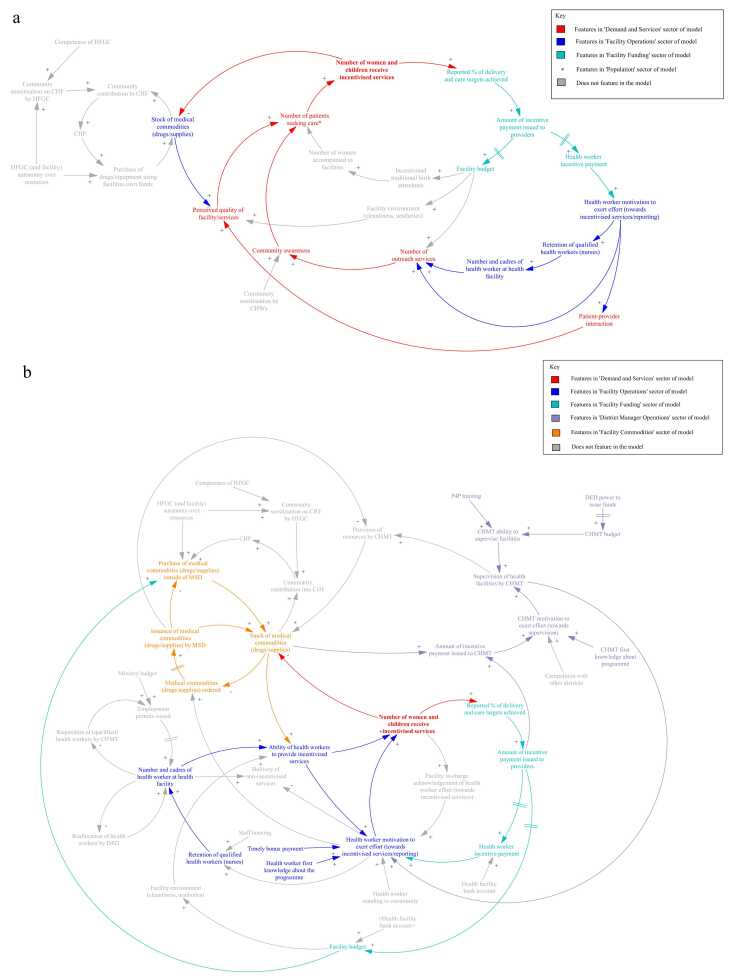
Structure identified in the previously developed CLD, that informed the (a) ‘demand’ and (b) ‘supply’ components in the simulation model. Adapted from Cassidy et al. (2021). Abbreviations: Community health fund (CHF); Community health worker (CHW); Council Health Management Team (CHMT); District Executive Director (DED); Health Facility Governing Committee (HFGC); Medical Stores Department (MSD); Payment for performance (P4P).

The first step for SDM development was to define the problem/health system behaviour to be investigated and the goals of the model. A previously developed CLD, which identified pathways to impact of P4P on delivery and coverage of maternal and child health services using the Pwani programme as a case study (Cassidy et al., 2021), was used as a blueprint for determining model purpose, sector selection and creation (Fig. 2). The health system behaviour explored with SDM was the performance of facilities during the P4P programme in Pwani. The goal of the SDM model was to (i) explore how facility performance responded to the P4P programme and (ii) test whether changes to implementation of the programme or its’ design can result in improved performance in a ‘typical’ primary care facility, (iii) explore how context affects programme outcomes. In the model, we have chosen to monitor the performance of a facility for two incentivised services; a content of care indicator (percentage of women who received two doses of IPT (IPT2) during ANC) and a coverage indicator (percentage of women who had a facility-based delivery), as these indicators showed some improvement during the P4P programme in Pwani and were the primary outcomes in the CLD. The model time step is months (the performance reporting unit), with simulation start time January 2011 and a time horizon of 54 months. The P4P programme in Pwani started with a preparation phase (2010) which included submission of routinely collected data by each participating facility, used to set performance targets at programme commencement. Programme implementation took place from January 2011. The Norwegian Ministry of Foreign Affairs provided funding to support implementation until December 2023, with the World Bank Health Innovation Trust Fund continuing to support the programme thereafter. This agreement was, however, only finalised in March 2015, resulting in delays in payment. The model simulation period traverses this timeline, starting with programme implementation (January 2011) continuing until July 2015 to consider both the short- and long-term effects of the programme which may fluctuate over time (Borghi et al., 2021).

#### 2.4.2 Data

The model was populated with both primary and secondary data sources, see Supplementary File 4 for full details of model variables and data sources used. Examples of secondary data include population and housing census reports and projections (National Bureau of Statistics 2013, 2018), country and district-level health surveys (National Bureau of Statistics 2011, 2015), reports describing human resources for health (Ministry of Health and Social Welfare, 2013, Ministry of Health and Social Welfare, 2014a, Ministry of Health and Social Welfare, 2014b) and evidence drawn from the literature e.g. quantifying the effect of number of antenatal care visits on probability of a facility-based delivery (Ensor et al., 2014), and attrition rate of health workers (Kurowski et al., 2007). Data from the impact evaluation conducted on the Pwani P4P programme was also used for model parameterisation and calibration, including information describing the incentive scheme, fraction of women who attend ANC visits, skill level of health workers at facilities and performance on incentivised services at facilities. The previous evaluation conducted on the Pwani P4P programme is described elsewhere (Binyaruka, 2015, Borghi, 2013, Borghi, 2021), with a summary provided here. The impact evaluation investigated the effect of the P4P programme on all targeted maternal and child health services (including percentage of women who receive IPT2 and percentage of women who seek facility-based delivery) through a controlled before and after study design. Surveys were conducted in all six districts of Pwani region (where P4P had been implemented) and in five control districts in neighbouring regions. The evaluation consisted of a health facility survey, health worker survey, exit survey of patients and survey of women who had delivered in the last 12 months. Data collection took place at three time points: ‘baseline’ (January 2012), ‘short term’ (February 2013) and ‘long term’ (February and March 2015).

#### 2.4.3 Model sectors

##### 2.4.3.1 Sector selection

**Fig. 3:**
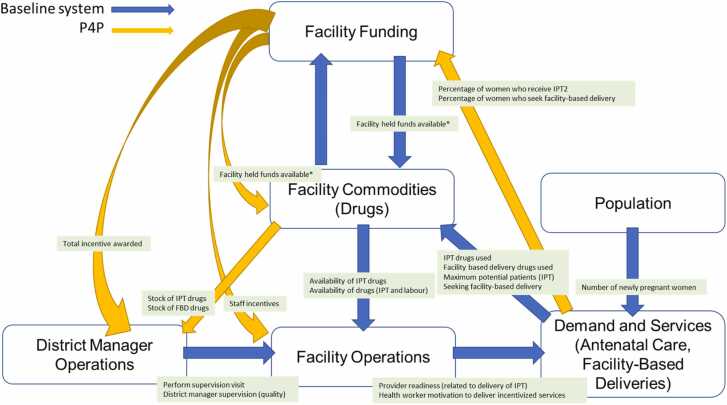
High level overview of simulation model. The model contains six subsectors which pass information to each other (arrows). The key outputs passed between each sector are shown in green boxes. The user can run the model with payment for performance switched 'on' and 'off', with blue (baseline system) and yellow (P4P) arrows showing what information is passed when P4P is switched ‘on’ and ‘off’ in the model. Note: *Facility held funds available features in the diagram twice as the composition of funding for facility operations changes as a result of P4P being switched ‘on’ in the model. Abbreviations: Facility based delivery (FBD); Intermittent preventative treatment (IPT); Payment for performance (P4P); Two doses of Intermittent preventative treatment (IPT2).

The second step for development was the creation of model sectors that drive behaviour in different compartments of the model. The CLD was used as a framework to inform development of the simulation model. Six model sectors were generated from the structures identified in the CLD (as shown in different colours in Fig. 2). Structures identified in the ‘demand’ component of the CLD (Fig. 2a) fed into development of the ‘**Demand and Services**’, ‘**Facility Operations’**, **‘Facility Funding’** and **‘Population’** sectors (Fig. 3). Structures identified in the ‘supply’ component of the CLD (Fig. 2b) fed into development of the ‘**Demand and Services**’, ‘**Facility Operations’**, **‘Facility Funding’**, ‘**District Manager Operations**’ and ‘**Facility Commodities**’ sectors (Fig. 3). Our main focus was on the facility level supply side dynamics related to facility performance as this was the primary target of P4P. As a result, the SDM does not capture a number of demand side elements from the original CLD, shown in ‘grey’ in Fig. 2 including: the dynamics around payment into a community health fund (voluntary community health insurance fund that was used to support provision of services at the facility), mechanisms for employing health workers and the activities of community health workers and traditional birth attendants in service demand creation. An agent-based model is currently under development which will explore the effect of community and service demand dynamics on facility-based deliveries.

##### 2.4.3.2 Simulation model overview

The purpose of each **sector**, *key sector outputs used as inputs to other sectors*, and a description of how sectors pass information are provided in this section. The model sectors and key sector outputs used as inputs to other sectors are shown in Fig. 3. Detailed individual model sector diagrams showing the underlying stock and flow structure and description of model equations are given in Supplementary Files 2 and 3, respectively. This section outlines the model functioning in the absence of P4P.

The **Population** sector controls population dynamics that feed into the **Demand and Services** sector**.** It controls ageing in the population (neonates, infants, pre-schoolers, children, reproductive age adults and adults above 50) over time, which is driven by the respective age mortality rates andfertility rate. The population dynamics for this sector mirror the natural phenomenon of ageing in the Tanzania population. The general function for an ageing population was adapted from Semwanga *et al.* (2016). The sector generates the following key output and population group of interest *number of newly pregnant women,* which contributes to the flow of patients seeking care in the **Demand and Services** sector. The population sector has been structured so that the model can be later adapted to focus on other types of service provision (e.g. infant vaccination).

The **Demand and Services** sector controls the number of ANC patients that receive services and facility-based deliveries. Patients can attend up to four ANC appointments, with three possible pathways for each ANC visit (i) dropping out and not attending ANC visit, (ii) receiving treatment (up to two doses of IPT across all ANC visits, with the goal of two doses for each patient during pregnancy) or (iii) do not receive treatment. These three options reflect the possible outcomes for ANC visits by patients in facilities. Treatment receipt is dependent on *provider readiness* to deliver care (controlled in the **Facility Operations** sector) and attendance rates for each antenatal care visit.

The percentage of facility-based deliveries is determined by (i) the number of antenatal care visits; (ii) distance to facility; (iii) awareness of maternal and child health and healthcare in the community (in part estimated from ability to perform outreach controlled in **Facility Operations** sector and fraction of women attending antenatal care); (iv) perceived quality of facility/services (estimated from an average of availability of drugs in **Facility Commodities** sector and patient-provider interaction from **Facility Operations** sector). This was modelled on the function for facility-based deliveries described in Semwanga *et al.* (2016), where the purpose of the model was to identify system strengthening policies to address neonatal mortality in Uganda. This equation was then further adjusted during model calibration to assign reduced weight to perceived quality of facility/services to ensure a better model fit (see Supplementary File 5 for further details).

For each patient who receives a service (ANC or facility-based delivery), a single unit of a drug is ‘used’ with drug availability depleting in the **Facility Commodities** sector (based on expected dispensing of medication per visit). The **Demand and Services** sector generates key outputs *percentage of women who receive IPT2* and *percentage of women who seek facility-based delivery.*

The **Facility Commodities** sector controls the replenishment and depletion of malaria (IPT) and labour drugs at the facility level. The expected number of ANC and facility-based delivery patients is fed in from the **Demand and Services** sector and used to place orders for drugs on a quarterly basis to the Medical Stores Department (autonomous government department responsible for provision of medical commodities), as is the practice for health providers in Tanzania. Depending on availability of drugs at Medical Stores, facilities may need to try and address the deficit of drugs. Facilities can use funds (facility held funds, managed in the **Facility Funding** sector) where available to purchase additional drugs. Key outputs in the **Facility Commodities** sector are the *availability of IPT drugs* and *availability of drugs (IPT and labour),* which deplete depending on the number of patients treated in the **Demand and Services** sector.

The **Facility Operations** sector manages facility-level dynamics including provider readiness (related to delivery of IPT). Provider readiness related to facility-based deliveries is not captured in the SDM but will be the focus of the in-development agent-based model, built to simulate service demand side dynamics related to facility-based deliveries. Provider readiness (related to delivery of IPT) is calculated as the minimum of availability of IPT drugs or average of (i) knowledge of health workers (IPT); (ii) number of health workers at health facility (percentage of positions filled); (iii) availability of IPT drugs fed in from the **Facility Commodities** sector; (iv) health worker motivation to exert effort towards incentivised services (as observed in the Tanzania CLD). This is so provider readiness to deliver services (related to IPT) does not exceed the stock of medicine available. Health worker motivation is calculated as an average of availability of drugs (IPT and labour) fed in from **Facility Commodities** sectors, district manager supervision (quality) fed in from **District Manager Operations** sector and number of health workers at health facility (percentage of positions filled). Key outputs in this sector are *provider readiness (related to delivery of IPT)* and *health worker motivation to exert effort towards incentivised services.*

The **Facility Funding** sector manages the funding that is held and used at the facility level and can be used to purchase additional drugs where needed. The key output from this sector is *facility held funds available*.

The **District Manager Operations** sector manages supervision visits by members of the Council Health Management Team to facilities. The district manager supervision (quality) is dependent on district level resources, management team motivation and the skill level of district managers. Supervision visits affect knowledge of health workers related to IPT and health worker motivation. The key outputs for this sector are *district manager supervision (quality) and perform supervision visit.* During model development, two members of the original programme evaluation team were consulted to provide insight into model dynamics related to impact of district manager supervision on health worker skill level. Model equations reflect this discussion, where effect of district manager supervision on health worker knowledge is dependent on the ‘base level’ of knowledge at the facility. Where this is lower, it will take a few supervision visits to raise the health worker knowledge (specifically related to provision of IPT during ANC).

##### 2.4.3.3 Introduction of P4P intervention

Health facilities are set targets they need to reach each cycle (6 months) to receive P4P incentive payments. Payment was to be made within three months of the conclusion of the six month performance cycle (Borghi et al., 2013), however, in practice payments were often delayed. In the model, the performance targets are for specific services monitored in the **Demand and Services** sector. These targets are percentage of women who receive IPT2 and percentage of women who seek facility-based delivery. Depending on performance against these targets, providers may receive incentive payments which are deposited in the **Facility Funding** sector.

The payment is split 75:25, with the larger portion allocated for health worker incentive payments and the remaining portion to be used to improve facility operations (e.g. purchasing additional medical commodities where needed) (Binyaruka et al., 2015). The health worker incentive payment is fed from **Facility Funding** to the **Facility Operations** sector. Incentive payments (specifically timeliness of payments) influence health worker trust in the programme and health worker motivation to exert effort towards incentivised services. The remainder of the incentive payment, in the model, supplements facility held funds (**Facility Funding sector**) and can be used to purchase drugs (malaria and labour drugs) where needed in the **Facility Commodities** sector. A new key output from the **Facility Funding** sector is *staff incentives*.

The district management team are also eligible for incentive payments, which are processed in the **District Manager Operations** sector, with payments influencing district manager motivation to support facilities. In the simulation model, the district management targets (and determinant of incentive payment issued) are to reduce stockouts of medicine (observed in the **Facility Commodities** sector) and overall performance of health facilities (observed in **Demand and Services** sector) (Borghi et al., 2013).

#### 2.4.4 Model validation

The third step for model development was subjecting the model to a series of verification and validation tests to build confidence in the structure, behaviour, and robustness of the model. To check for internal validity, every equation in the model was reviewed for dimensional consistency i.e. that model units were appropriate for the given variable i.e. population parameters are measured in units of ‘persons’, and that units used for outputs were appropriate based on variable input units. The model was also subjected to extreme condition testing, whereby selected model parameters were adjusted to extreme values and model output was evaluated to ensure expected results. For example, when the dropout rate for attending a first ANC visit is 0.999, we expect only a handful of patients to attend this first visit and move through the ANC part of the demand and services sector; or when the provision of medicine by the Medical Stores is severely impacted, we expect to see a drastic depletion of medicine available at facilities. The model performed well when subjected to testing, producing expected behaviour under extreme conditions. Model equations and structure were also independently reviewed by a team member. To check for external validity, selected model output projections were also compared to real data where available, with equation and parameter adjustments made where required so that model outputs were aligned with data (model calibration). The model was adequately able to replicate known trends, see Supplementary File 5 for further details on how selected inputs were calibrated to data.

To check model face validity, the resulting model was presented to nine key stakeholders involved in the implementation or evaluation of the Pwani programme during virtual interviews (conducted via Zoom) as a final validation step. A model interface was developed using Stella Architect to assist with presentation of model outputs and key assumptions, see Supplementary File 6 for the interview guide and details on model interface. The interview consisted of two segments, where stakeholders were first shown key model output and dynamics and asked to comment on whether model behaviour was realistic and aligned with their experience of the P4P programme, and then shown model assumptions and asked to provide feedback on their validity.

The feedback received during these interviews resulted in some new additions and adjustments to existing model structure (see Supplementary File 7 for details). Stakeholders also reflected on the presentation of the model, commenting that a high-level diagram showing how the model worked would be useful to them (see Fig. 3).

Stakeholders remarked on the importance of community health workers and traditional birth attendants in increasing community awareness of services and escorting women to facilities for facility-based deliveries. These dynamics are not included in this current version of the model for the reasons set out above (see sector selection).

#### 2.4.5 Sensitivity analysis

The final step for model development was subjecting the model to sensitivity analyses to determine the sensitivity of key outcomes (percentage of women who receive at least two doses of IPT during ANC, percentage of women who seek facility-based delivery) to changes in model parameters. Model parameters deemed appropriate for analysis (see Supplementary File 8) were adjusted by 10 %, with key outcome results recorded. The following scale was used to determine sensitivity to changes in model variables; sensitive (5 % ≤ change in outcome < 15 %), very sensitive (15 % ≤ change in outcome < 25 %) and highly sensitive (change in outcome ≥ 25 %). The scale is adapted from Semwanga *et al.* (2016) and presented with smaller intervals for higher sensitivity categories, to further distinguish ‘very sensitive’ from ‘highly sensitive’ results. As the variables included in the sensitivity analysis reflect health system characteristics, this analysis also shed light on the likely effect of changes to the health system context in which P4P is implemented on key outcomes.

### 2.5 Simulation and scenario testing

Model scenarios were selected to contribute evidence towards the knowledge gap identified by reviews of P4P effects in LMIC settings (Das et al., 2016, Diaconu, 2021, Patel, 2018); to further understanding on pathways to effect for P4P, acknowledging the influence of programme design, implementation and context.

The model was first used to explore how health system performance changed under P4P, to examine the effects of the programme as it was implemented (as mentioned, there were delays in issuing programme incentive payments) on pathways to effect for the programme (e.g. health worker motivation, availability of medicine) and targeted services, percentage of women who receive at least two doses of IPT during ANC and percentage of women who seek facility-based delivery. The effect of changes in programme implementation (payments made on time vs. with delays) and design (adjusting the share of funds between staff incentives and funds to strengthen facility operations) on these outcomes were then tested in the model. The effect of payment delays on procurement of additional medicines in the **Facility Commodities** sector is logical (health providers can purchase medicine when funding is available, unable to purchase when funds are unavailable). The effect of payment delays on health worker motivation and trust in the **Facility Operations** sector was determined through stakeholder consultation (see Supplementary File 7 for details). The variable ‘use of incentives’ in the **Facility Funding** sector was altered to adjust the share of funds between staff incentives and funds to strengthen facility operations.

Finally, the sensitivity analysis results were used to explore the effect of changes to programme and health system contextual factors (including provision of medicine from Medical Stores, amount of alternative facility held funding and staffing levels) on targeted services, percentage of women who receive at least two doses of IPT during ANC and percentage of women who seek facility-based delivery.

## 3 Results

### 3.1 Unpacking the mechanisms of P4P

**Fig. 4:**
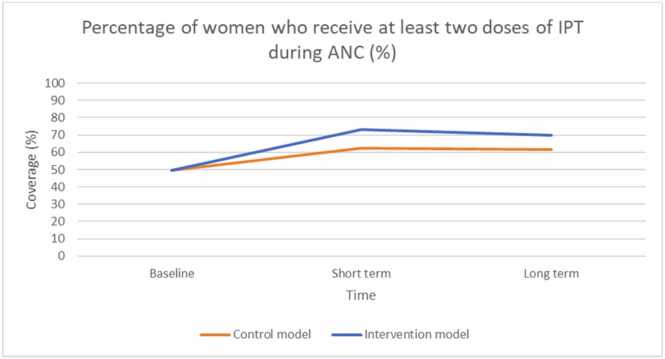
O1: Model output for percentage of women who receive at least two doses of IPT during ANC (%) in the control and intervention groups. Abbreviations: Antenatal care (ANC); Intermittent preventative treatment (IPT); Output (O).

**Fig. 5:**
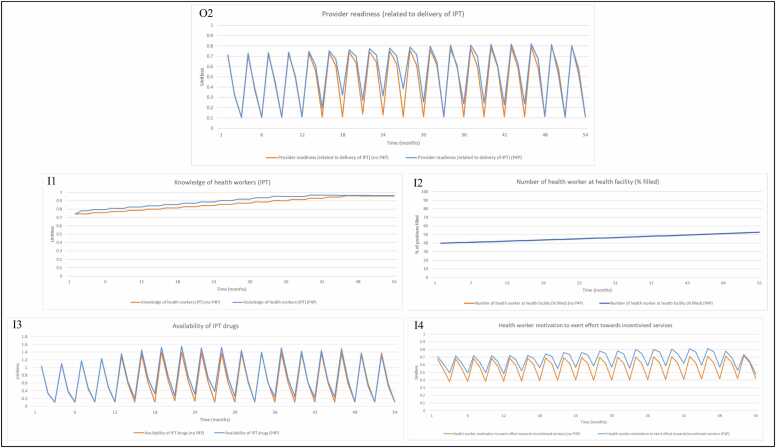
Model output for (O2) provider readiness (related to delivery of IPT during ANC) with inputs (I1) knowledge of health workers (IPT), (I2) number of health workers at health facility (% filled), (I3) availability of IPT drugs, (I4) health worker motivation to exert effort towards incentivised services, when P4P is turned ‘on’ and ‘off’ in the model. Note 1: In the model, number of health workers at health facility (% filled) is unaffected by P4P but included as input to provider readiness to deliver services (related to delivery of IPT). Note 2: The axis for Fig. 5 (I3) has different numerical bounds compared to the standard axes in other graphs. Abbreviations: Input (I); Intermittent preventative treatment (IPT); Output (O); Payment for performance (P4P).

As in the original intervention evaluation, we observe the stark contrast between intervention and control sites at the short-term evaluation, before performance starts to drop off in the intervention group at the long term evaluation for percentage of women who received two doses of IPT (Fig. 4, O1). The change in performance observed in the intervention group in the model is attributed to changes in provider readiness (related to the delivery of IPT) (Fig. 5, O2), which is a factor of knowledge of health workers in delivery of IPT (Fig. 5, I1), number of health workers at the facility (% filled) (Fig. 5, I2), availability of IPT drugs (Fig. 5, I3) and health worker motivation (Fig. 5, I4). Availability of IPT drugs and health worker motivation to deliver incentivised services experienced the most change as a result of the programme, and are driving improvements in the IPT during ANC outcome.

Availability of IPT drugs increases when a delivery is made from the Medical Stores Department (every three months) or when facility held funding is used to purchase drugs (Fig. 5, I3). Where incentive payments are received by facilities (months 13, 18, 22, 27, 34 and 42) these are used to purchase additional drugs and improve drug availability. Availability of IPT drugs is volatile, exhibiting improved behaviour where funds are available to purchase more drugs outside Medical Stores, and behaviour closer to the control group where additional funds are not available. This extreme volatility is reflected in provider readiness (Fig. 5, O2), increasing when drugs are procured from the Medical Stores every three months, depleting over a three month period as drugs are dispensed to patients, with volatility somewhat stemmed when P4P payments are used to purchase additional medicines.

**Fig. 6:**
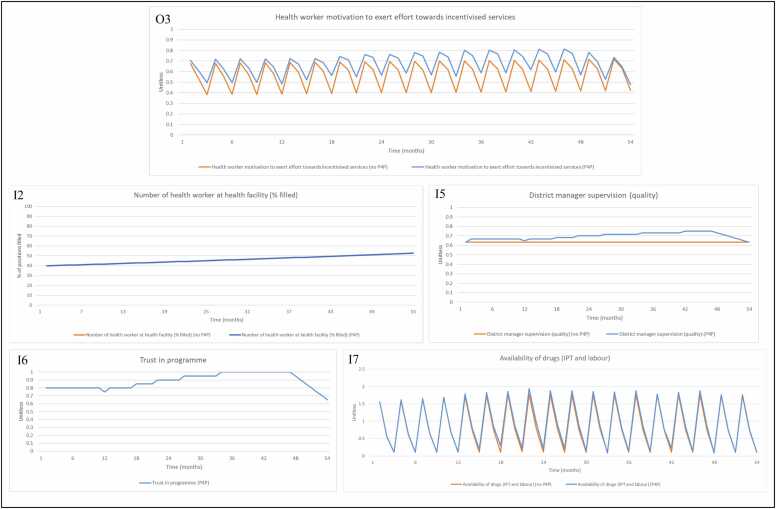
Model output for (O3) health worker motivation to exert effort towards incentivised services with inputs (I2) number of health workers at health facility (% filled), (I5) district manager supervision (quality), (I6) health worker trust in programme, (I7) availability of drugs (IPT and labour), when P4P is turned ‘on’ and ‘off’ in the model. Note 1: In the model, number of health workers at health facility (% filled) is unaffected by P4P but included as input to health worker motivation to exert effort towards incentivised services. Note 2: In Fig. 6 (I6) ‘Trust in programme’ only exists (and impacts health worker motivation) when the P4P programme is switched ‘on’ in the model. Note 3: The axis for Fig. 6 (I7) has different numerical bounds compared to the standard axes in other graphs. Abbreviations: Input (I); Intermittent preventative treatment (IPT); Output (O); Payment for performance (P4P).

Health worker motivation (Fig. 6, O3) is fluctuating as a result of changes in district manager supervision (quality) (Fig. 6, I5), trust in the P4P programme (Fig. 6, I6) and availability of all drugs (Fig. 6, I7), which exhibits a similar trend to availability of IPT drugs. Trust in the programme gently increases whenever payments are made but decreases when there are severe (4 + months) delays in payment, with district manager supervision also exhibiting this trend.

**Fig. 7:**
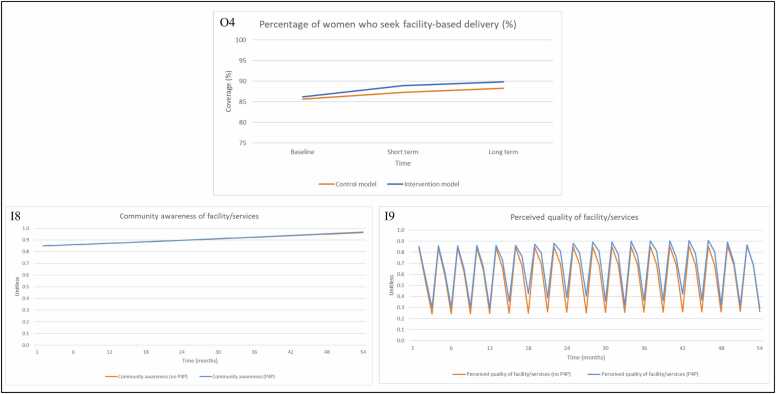
Model output for (O4) percentage of women who seek facility-based deliveries (%) in the control and intervention groups with inputs (I8) community awareness, (I9) perceived quality of facility/services, when P4P is turned ‘on’ and ‘off’ in the model. Note 1: The axis for Fig. 7 (O4) has different numerical bounds compared to the standard axes in other graphs. Abbreviations: Input (I); Output (O); Payment for performance (P4P).

For facility-based deliveries, we observe an improvement between the intervention and control sites for the short term and long term evaluations (Fig. 7, O4). The change in performance observed in the intervention group is attributed to changes in community awareness of facility and services (Fig. 7, I8) and perceived quality of facility and services (Fig. 7, I9). In the model, P4P has very limited impact on provider behaviour in performing outreach activities. If outreach activities were to increase, it would drive improvement in community awareness of facility services, which would lead to an increase in facility-based deliveries. The change in percentage of women delivering in health facilities as a result of P4P is therefore driven in the model by the perceived quality of the facility and services. Perceived quality is constrained by medicine availability, with improvements seen as a result of increased drug availability and health worker motivation (taken as a proxy for patient-provider interaction in the model).

### 3.2 Effect of changes to programme implementation and design

#### 3.2.1 Model with and without payment delays

The model was run with payment delays switched ‘on’ and ‘off’ to observe the impact of delays on the delivery of incentivised services. Where delay in payments is switched ‘off’, payments are made as was originally planned, every six months (3 months after each performance cycle, i.e. month 9, 15, 21 etc.). Where delay in payments is switched ‘on’, payments are made according to the actual schedule of payments that took place (months 13, 18, 22, 27, 34 and 42).

**Fig. 8:**
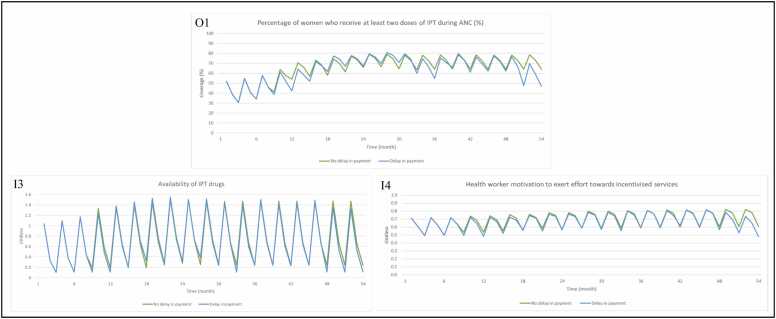
Model output for (O1) percentage of women who receive at least two doses of IPT during ANC (%), with inputs (I3) availability of IPT drugs and (I4) health worker motivation to exert effort towards incentivised services, via provider readiness (related to delivery of IPT during ANC), when payments are made on time vs. when payments are delayed. Note 1: The axis for Fig. 8 (I3) has different numerical bounds compared to the standard axes in other graphs. Abbreviations: Antenatal care (ANC); Input (I); Intermittent preventative treatment (IPT); Output (O).

**Fig. 9:**
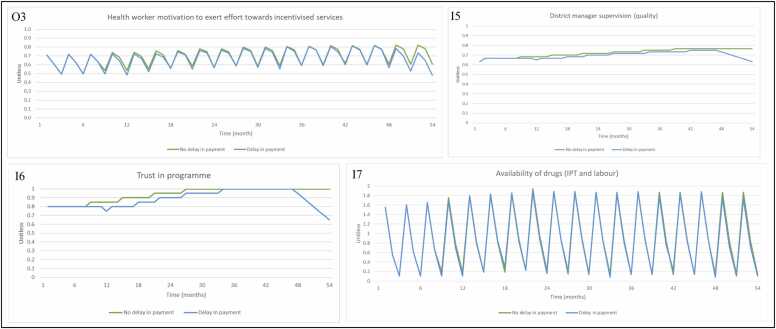
Model output for (O3) health worker motivation to exert effort towards incentivised services with inputs (I5) district manager supervision (quality), (I6) health worker trust in programme, (I7) availability of drugs (IPT and labour), when payments are made on time vs. when payments are delayed. Note 1: The axis for Fig. 9 (I7) has different numerical bounds compared to the standard axes in other graphs. Abbreviations: Input (I); Intermittent preventative treatment (IPT); Output (O).

When there are no payment delays, percentage of women who receive two doses of IPT (Fig. 8, O1) rises and falls as a result of changes in availability of IPT drugs (drug replenishment by the Medical Stores Department, procurement of drugs using P4P payments; and drug depletion through dispensing to patients) (Fig. 8, I3) and health worker motivation (district manager supervision visits, increasing trust in the programme as payments are being made on time and availability of all drugs driving changes) (Fig. 8, I4). In the absence of delays, further improvement in availability of IPT and labour drugs (Fig. 8, I3 and Fig. 9, I7) is observed as a result of P4P payments. There are also improvements (compared to the no P4P scenario) in health worker motivation (Fig. 9, O3), due to periodic increases in the quality of supervision (Fig. 9, I5) and health worker trust in the programme (Fig. 9, I6) each time a payment is made, and continued availability of drugs (Fig. 9, I7). Short term delays in payment (those less than 4 months) suspend improvement in health worker trust in programme (and therefore motivation) and quality of supervision, which then improve when payments are made. Impact on availability of drugs from short terms delays is minimal as payments are used in the next month or so to recover stock. When there are severe delays in payment (4 or more months), as seen from month 48, trust in the programme and quality of supervision decrease and struggle to recover. Prolonged reduction in funding also negatively impacts availability of drugs and ability to deliver incentivised services.

**Fig. 10:**
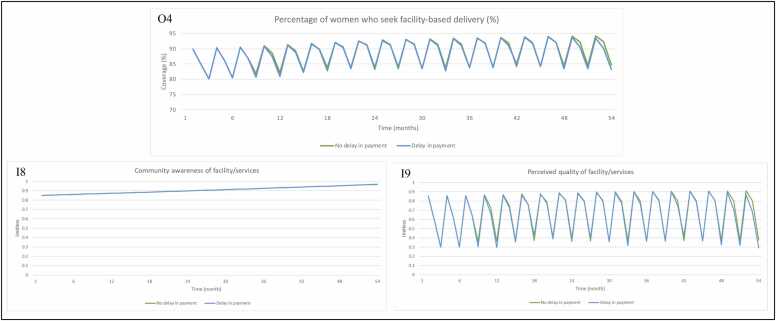
Model output for (O4) percentage of women who seek facility-based deliveries (%) with inputs (I8) community awareness, (I9) perceived quality of facility/services, when payments are made on time vs. when payments are delayed. Note 1: The axis for Fig. 10 (O4) has different numerical bounds compared to the standard axes in other graphs. Abbreviations: Input (I); Output (O).

Percentage of women who seek facility-based delivery slightly improves under the no delay scenario (Fig. 10, O4). There is no noticeable improvement in community awareness of facility and services (Fig. 10, I8), with a slight improvement in perceived quality of facility/services (through consistent improvements in drug availability and health worker motivation) (Fig. 10, I9).

#### 3.2.2 Model with changes to allocation and use of payment

The model was then run with changes to the design of the P4P programme. In the original programme design, 75 % of the incentive payment was to be split between staff at the facility, with the remaining 25 % to be used to improve facility operations (e.g., purchasing additional medicine where needed). The share of funds between staff incentives and facility operations was adjusted and tested in the model, comparing the 10:90, 25:75, 50:50, 75:25 (original design), 90:10 payment shares to the no P4P scenario. These design changes were first simulated in a no payment delay context, before simulating the design changes with payment delays.

**Fig. 11:**
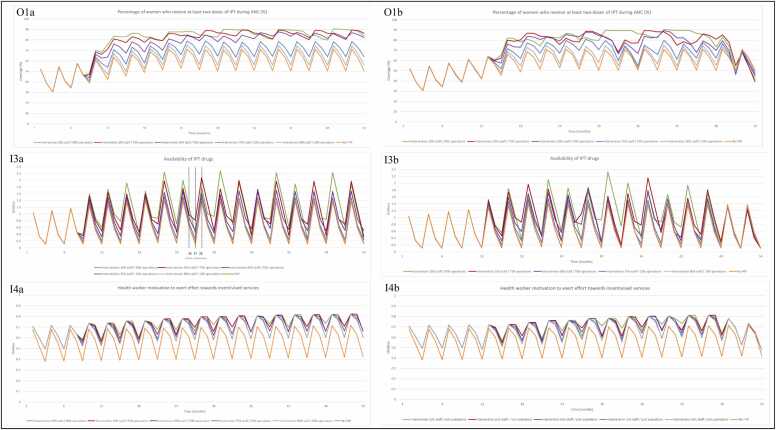
Model output when allocation of payments is adjusted(O1)percentage of women who receive at least two doses of IPT during ANC (%) with inputs (I3) availability of IPT drugs, (I4) health worker motivation to exert effort towards incentivised services, via provider readiness (related to delivery of IPT during ANC). The left-hand column shows simulation results when (a) payments are made on time vs. (b) the right-hand column when payments are delayed. Note 1: The axes for Fig. 11 (I3a and I3b) have different numerical bounds compared to the standard axes in other graphs. Abbreviations: Antenatal care (ANC); Input (I); Intermittent preventative treatment (IPT); Output (O); Payment for performance (P4P).

**Fig. 12:**
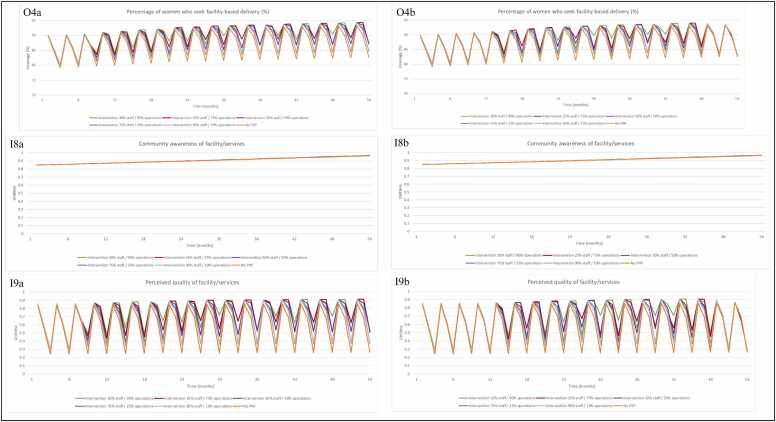
Model output when allocation of payments is adjusted (O4) percentage of women who seek facility-based deliveries (%) with inputs (I8) community awareness, (I9) perceived quality of facility/services. The left-hand column shows simulation results when (a) payments are made on time vs. (b) the right-hand column when payments are delayed. Note 1: The axes for Fig. 12 (O4a and O4b) have different numerical bounds compared to the standard axes in other graphs. Abbreviations: Input (I); Output (O); Payment for performance (P4P).

When the allocation of incentive payments between staff and facility operations is adjusted and there are no delays in payment, we see a direct impact on both incentivised services (Fig. 11, O1a, Fig. 12, O4a). Fig. 11 demonstrates the importance of the facility operations component of the incentive for achieving model outcomes – the 10:90 share of funds between staff and facility operations design resulted in the greatest improvement to incentivised services and key outcomes, with the 90:10 share performing worse than the original P4P design. With a higher level of facility operation funding, more funds are available to purchase drugs. This eases the burden of inadequate stock (Fig. 11, I3a) and enables provision of care for more patients, as we see for the outcome, percentage of women who receive two doses of IPT outcome (Fig. 11, O1a). As availability of drugs also affects health worker motivation, this also improves the IPT outcome through this pathway (Fig. 11, I4a). Over the time horizon of the simulation, the 10:90 share of funds design outperforms the 25:75 share of funds design for improvement in incentivised services (Fig. 11, O1a, Fig. 12, O4a). However, the 25:75 design intermittently supersedes the 10:90 design, which is attributed to the threshold for ordering additional drugs in the model, observed in Fig. 11, I3a at time-step 26–28.^1^

When payments are made according to the actual schedule of payments observed during programme implementation (with delays), the 10:90 share of funds design still produces the greatest improvement in incentivised services over time (Fig. 11, O1b, Fig. 12, O4b), but the overall improvement observed during the simulation period (Fig. 11, I3b and I4b, Fig. 12, I8b and I9b) is worse than the simulations without payment delays. As observed in previous simulations, percentage of women who seek facility-based deliveries is less sensitive to changes in drug availability as the target is not entirely dependent on availability of labour drugs (Fig. 12, O4a). The change in payment design has little effect on community awareness of facility and services (Fig. 12, I8a). We do observe improvement in perceived quality of care (Fig. 12, I9a) as a result of increasing allocation of payment towards facility operations, but the overall effect on facility-based deliveries is less acute than for the IPT outcome.

### 3.3 Sensitivity analysis

Further analyses were performed to examine the sensitivity of key outcomes (percentage of women who receive at least two doses of IPT during ANC, percentage of women who seek facility-based delivery) to changes in model parameters, including the health system context, (see Supplementary File 8 for details and full results) under the programme. Adjustments to community awareness, distance to facility and Medical Stores Department provision of drugs prompted a ‘sensitive’ response from key outcome ‘percentage of women who receive at least two doses of IPT during ANC’ (Table 1), with low sensitivity observed across all variables for the other key outcome, percentage of women who seek facility-based delivery.

**Table 1:** Sensitivity analysis to examine the sensitivity of key outcomes (percentage of women who receive at least two doses of IPT during ANC, percentage of women who seek facility-based delivery) to changes in model parameters, with results indicating sensitivity to model parameters presented here.

Note 1: The following scale was used to determine sensitivity to changes in model variables; sensitive (5 % ≤ change in outcome < 15 %), very sensitive (15 % ≤ change in outcome < 25 %) and highly sensitive (25 % ≥ change in outcome). Table cells are highlighted where outputs are categorised as sensitive (yellow), very sensitive (orange) and highly sensitive (red).

Note 2: (a) initial stock value adjusted (b) constant variable value(s) adjusted.

Abbreviations: Antenatal care (ANC); Intermittent preventative treatment (IPT); Medical Stores Department (MSD).

It is clear that provision of IPT by the Medical Stores Department has a marked effect on the percentage of women who receive at least two doses of IPT during ANC, that can facilitate or prevent facility achievement of the target, producing a ‘sensitive’ response from this key outcome. There is some effect on percentage of women who seek facility-based delivery, through the perceived quality of facility and services pathway. For percentage of women who receive at least two doses of IPT during ANC, it is around the ‘baseline’ period that additional provision of drugs by the Medical Stores has the greatest effect (although a marked effect is noted throughout the simulation). This is attributed to facilities having more alternative funding (P4P incentive payments) after this period, where providers could purchase drugs, funding permitting.

Reducing the initial stock value for community awareness of services and constant variable distance to facility has a small negative effect on percentage of women who seek facility-based delivery, but surprisingly a significant short-term knock-on effect to percentage of women who receive at least two doses of IPT during ANC. Reducing the values of these parameters by a small amount resulted in ‘sensitive’ model behaviour and a large reduction to IPT during ANC outcome; closer inspection of model behaviour revealed that performance on the facility-based delivery target was reduced in previous months, which led to targets (in month 6 and 12) being missed. This resulted in a reduction in P4P payments and therefore in funding available to purchase medicine, leading to a reduction in percentage of women who received at least two doses of IPT during ANC. Once percentage of women who sought facility-based delivery surpassed the 85 % target later in the simulation (month 18), providers just needed to maintain this average to achieve targets (Supplementary File 1, Table S1.1) and consequently P4P funding returned to previous levels.

Adjustments to all other model parameters did not elicit sensitive model behaviour, including other contextual factors, such as alternative sources of funding available to facilities and number of health workers. Altering the amount of alternative facility held funding in the model produced only minor changes in key outcomes (Supplementary File 8, Table S8.14). The amount of assumed alternative funding in the model is already limited, with modifications by 10 % only adjusting the funding amount by 1 %. Altering the amount prevents or facilitates providers from purchasing much needed medications, impacting facility performance on incentivised targets. Impact is greater for percentage of women who receive at least two doses of IPT during ANC, as this target is heavily dependent on availability of drugs.

Adjusting number of health workers at health facility (% of positions filled) seems, initially, to have little effect on percentage of women who receive at least two doses of IPT during ANC (Supplementary File 8, Table S8.12). Small improvement in number of health workers leads to a small initial improvement in provider readiness to deliver services, resulting in more patients treated. However, this means that facilities have a reduced level of drugs to treat patients with, balancing out the previous improvement in the IPT during ANC outcome. Adjusting number of health workers has very little effect on percentage of women who seek facility-based deliveries in the model; although this improves ability to perform outreach, the impact on community awareness of services and fraction who seek facility-based deliveries is minimal.

## 4 Discussion

This paper describes the development and application of a SDM to explore the mechanisms through which a P4P programme affected maternal and child health service delivery outcomes in a primary care facility setting and the effect of changes in the design, implementation, and context of a P4P programme in a LMIC setting, Tanzania. The feasibility of building a SDM from a CLD is demonstrated, with the model subjected to internal, external and face validity testing.

Each time a payment is made in the model, health worker trust in the programme and motivation to deliver incentivised services gently increase, and facilities use the funding to purchase additional needed medications to support service delivery, this also improves perceived quality of care, influencing demand for institutional deliveries. Minor delays in programme payments (less than 4 months) had a minimal impact on provider performance of incentivised services. Severe delays in programme payments limited provider capacity to achieve targets for the IPT during ANC indicator, due to reduced provider purchasing power for medicines. Prolonged delays also resulted in erosion of provider trust in the programme and reduced motivation for programme participation. As the facility-based deliveries indicator was not entirely beholden to drug availability, a smaller negative effect on the outcome was observed here (through the perceived quality of care pathway, which is affected by changes in drug availability and health worker motivation). Model results show facility funding is a key driver of P4P programme success, with increased allocation of funding towards strengthening facility operations (e.g. purchasing additional drugs for service delivery) leading to greater improvements in coverage and content of care for maternal and child health services. For the content of care indicator (2 doses of IPT during ANC), allocating a higher proportion of funding for facility operations alleviates the burden of inadequate stock and enabled provision of care for more patients, whilst also impacting health worker motivation. Allocating a higher proportion of funding for facility operations also had a positive effect on the coverage of care indicator (facility-based deliveries), through the perceived quality of care pathway, but the effect is less acute when compared to the content of care indicator.

The sensitivity analyses also identified three relevant contextual factors which have a significant effect on facility ability to achieve targets: with P4P schemes being more effective where there is adequate provision of IPT by the medical stores department at baseline, community awareness of facility services, and where facilities don’t serve very dispersed/distant populations. We also identified dependencies between the two target indicators, with lower performance on facility-based deliveries resulting from lower community awareness or greater distance to facilities, leading to a reduction in performance payments which impacted provider purchasing power for ANC medication, limiting the IPT during ANC target.

In this study, programme implementation, design and context were shown to be critical determinants of provider performance during P4P. This study contributes further evidence on how, why and under what circumstances P4P does (or does not) work in LMIC settings, and how P4P design influences pathways to impact and health system outcomes, cited as critical areas for future research by a recent realist review of P4P in LMIC settings (Singh et al., 2021). Although the P4P programme accounted for baseline performance of facilities in setting targets, study results indicate further refinement of how funding is allocated for facilities may produce further improvements in performance; for example, those facilities who have low drug availability before programme implementation would benefit from a higher share of funds towards facility operations. Study results also indicate that the effect of certain design features is not necessarily uniform across performance targets within a given P4P scheme, while incentives for strengthening facility operations (specifically purchasing of essential medicines) was a critical pathway for improvement for the content of care indicator (2 doses of IPT during ANC) this had less impact for the coverage indicator (facility-based deliveries) which depended on demand stimulation. Model results also demonstrate that programme effects are not constant over time and can vary substantially, fluctuating in response to stimuli and events in the wider system overcoming the limitation of cross-sectional or one time evaluation assessments that struggle to identify and disentangle such dynamic system behaviour.

To our knowledge, there have been five applications of CLD and SDM methodology to explore the effect of P4P programmes in LMICs; all five articles present research with CLDs (Alonge, 2017, Cassidy, 2021, Meker and Barlas, 2015, Renmans et al., 2017, Singh, 2021), with two articles also using the CLD to develop a SDM (Alonge, 2017, Meker and Barlas, 2015). Renmans et al. (2017) mapped a P4P programme in Uganda, similarly identifying supervision and work environment (availability of equipment and medicine) as key mechanisms influencing health worker motivation and performance during P4P. Work environment was aggregated at a high level in the Uganda CLD, without teasing out procurement and supply chain processes, which proved to be a critical bottleneck for provider performance in the current study. Singh et al. (2021) used a CLD to visualise the results from a realist review of P4P in LMIC settings. The realist review and current study both highlight patient provider interactions, availability of medicine and outreach activity as pathways through which P4P programmes impact patient uptake of services; the current study contributes further evidence on attributes of provider readiness (staffing, drug availability) influencing health worker motivation, and through this pathway, provider performance during P4P. As the framework for the CLD presented in Cassidy et al. (2021) was used to develop the current model, there are broad similarities in their structural composition. What the current study adds, and adds to the aforementioned CLDs of P4P, is simulation of the programme over time and therefore capacity to test design and implementation changes and effect of contextual factors on health system behaviour.

The current study and Alonge et al. (2017) both model the effect of P4P on health worker motivation and quality of services. Although availability of drugs, an important input to facility readiness in the current study, features in Alonge et al. (2017) as part of an aggregated quality variable, supply chain mechanics are not present in the model. In the current study, exploration of this process proved key to identifying where bottlenecks were occurring, with reflection on how support of procurement and supply of medicines may be integrated into the design of P4P. Alonge et al. (2017) and the current study both simulate the effect of payment delays on provider performance. Whilst Alonge et al. (2017) assume system performance will eventually follow the same trajectory as when there are no delays in payment, in the current study, minor delays impact service delivery (procurement of additional medicines) but have minimal impact on provider motivation and trust in the programme. Alonge et al. (2017) do not explore the effect of major payment delays on provider behaviour and service outcomes, or impact of changes to allocation and use of payments, results which are presented in the current study. There is little overlap between the content and results from Meker and Barlas (2015) and the current study, aside from observation on the effect of P4P on providers seeking to treat more patients. The model crucially doesn’t feature provider readiness to deliver services which was critical in the current SDM to understanding the effect of the programme on service delivery. The resource constraints faced by providers in lower income settings is also not accounted for in the model, making it difficult to generalise results to settings like Tanzania.

There are several limitations to this study. The model does not capture patient morbidity, mortality or health outcomes (likely to be affected by the programme), instead focussing on coverage and content of care for facility-based services as these were the primary targets measured by the programme, providing data on which to build the model. Certain community-level and care-seeking dynamics, such as the role and impact of community health workers and effect of peer networks on patient decision making, could not be captured in the current version of the model due to the level of aggregation required. The composition of heterogenous drivers for motivation was also difficult to capture in the SDM, including how individual health worker characteristics impact motivation and are affected by P4P programmes. An agent-based model that focusses on care-seeking behaviour for maternal services and health worker behaviour during P4P programmes is currently under development. Agent-based models enable simulation of individual ‘agents’ (patients, health workers), each with their own characteristics, that make decisions based on these attributes, the actions of other agents and events that take place in the wider system (Badham, 2018, Tracy et al., 2018). Agent-based models are ideally suited to capture these micro-level dynamics, such as the drivers and behaviour for individual actors, relevant for studying the impact of schemes like payment for performance. The model is currently being developed as a standalone model, with plans for a hybrid simulation that will enable analysis of both micro and macro-level health system behaviour during P4P programmes.

Assumptions were made for certain model parameters and functions where it proved difficult to draw from existing data sources. Stakeholder feedback was used to shape certain assumptions to induce realistic system behaviour (such as impact of payment delays on delivery of services and trust in the programme). The model underwent various verification and validity tests (internal, external and face validity) but was not subjected to a test of generalisability, checking model robustness and ability to replicate system structure and behaviour in another setting. A test of generalisability is currently underway for the previously described CLD of the programme in Tanzania (Cassidy et al., 2021) to a comparable P4P programme in Zambia (Shen et al., 2017), with motivation to also test the generalisability of the SDM using the Zambia programme as a case study. In testing model generalisability, we can ascertain whether the underlying structure of the CLD and SDM are relevant for settings with comparable programmes (e.g. Tanzania and Zambia) and also the extent to which the results and conclusions drawn from the Tanzania study are relevant outside this country context. Key results will include identifying those central structures that do not differ between country settings (are generalisable) and those which do differ and are relevant for understanding the implementation and impact of P4P in different settings.

There is a global movement underway, with focus shifting from P4P style health system strengthening programmes towards Direct Health Facility financing (Kapologwe, 2019, de Walque and Kandpal, 2022). In line with goals for P4P, these programmes also aim to improve healthcare quality, reduce health system and service inefficiencies, and better mobilise facility and community human resources for strengthened service delivery (Mæstad et al., 2021); however, the design of Direct Health Facility financing programmes place more weight on provider autonomy and funding to improve facility operations. The results from this current study potentially support this change in programme design, with clear benefits to higher allocation of funding towards facility operations in low resource settings. Study results indicated that this funding design would have greatest improvement on content of care services such as IPT2 during ANC; coverage of services targets like facility-based deliveries would see greater improvement with focussed funding and support for outreach activities to enhance service coverage. Effectual implementation of the programme, specifically timely bonus payments, will strengthen pathways to impact for the programme to improve healthcare service delivery outcomes.

## Ethics approval and consent to participate

This study received a favourable ethical opinion from the Observational/Interventions Research Ethics Committee at The London School of Hygiene and Tropical Medicine (LSHTM Ethics Ref: 16139 – 2), the Institutional Review Board at Ifakara Health Institute (IHI/IRB/No:15–2019) and National Institute for Medical Research (NIMR/HQ/R.8a/Vol. IX/3154) in Tanzania.

## Funding

This research is funded by the 10.13039/501100000265Medical Research Council under the Health Systems Research Initiative grant (MR/R013454/1).

## CRediT authorship contribution statement

**Rachel Cassidy:** Conceptualization, Data curation, Formal analysis, Investigation, Methodology, Visualization, Writing – original draft, Writing – review & editing. **Agnes Rwashana Semwanga:** Methodology, Supervision, Writing – review & editing. **Peter Binyaruka:** Data curation, Resources, Writing – review & editing. **Karl Blanchet:** Conceptualization, Funding acquisition, Writing – review & editing. **Neha S. Singh:** Conceptualization, Funding acquisition, Writing – review & editing. **John Maiba:** Investigation, Writing – review & editing. **Josephine Borghi:** Conceptualization, Data curation, Funding acquisition, Methodology, Resources, Supervision, Writing – review & editing.

## Declaration of Competing Interest

The authors declare that they have no known competing financial interests or personal relationships that could have appeared to influence the work reported in this paper.

## Data Availability

Model description and equations are given in the Appendix.

## Footnotes

Appendix A Supplementary data associated with this article can be found in the online version at doi:10.1016/j.ssmhs.2024.100040.

## Footnotes

1 *In*Fig. 11*(I3a), time step 26, 27 and 28 (identified with dotted lines) illustrate where the 25:75 design performs better than the 10:90 design. In the model, when drug availability falls below ‘1’ (when there is an inadequate supply of drugs for patients seeking care), providers can use P4P payments to purchase additional drugs. At time step 26, this criteria for purchasing additional drugs is met for the simulation with the 25:75 design but not for the simulation with the 10:90 design. At time step 27, the availability of drugs is therefore lower for the 10:90 simulation than 25:75 simulation, because providers did not use additional funding to purchase drugs at the previous time step. For both simulations at time step 27, providers use additional funding to purchase drugs and also receive a delivery from the Medical Stores Department. At time step 28, availability of drugs is still most improved under the 25:75 simulation before dropping back below the performance of the 10:90 design simulation at time step 29.*
