# Supplementary material for: Identifying the active ingredients in payment for performance programmes using system dynamics modelling
Source: SSM Health Syst. 2025 Jun;4:100040. doi: 10.1016/j.ssmhs.2024.100040 (PMC12062200; doi:10.1016/j.ssmhs.2024.100040)
Supplement: Supplementary file 1 — Supplementary material [file mmc1.docx]

### Supplementary material

*Supplementary File 1 - Facility and district manager indicators and performance targets during the Pwani P4P programme in Tanzania*

Table S1.1: Facility coverage, content of care and HMIS strengthening indicators and performance targets set during the Pwani P4P programme in Tanzania.

| **Indicator** | **Measure** | **Baseline coverage (previous cycle)** | | | | |
| --- | --- | --- | --- | --- | --- | --- |
|  |  | **0-20%** | **21-40%** | **41-70%** | **71-85%** | **85%+*** |
| *Coverage indicators* |  |  |  |  |  |  |
| % of institutional deliveries | Percentage point increase | 15% | 10% | 5% | 5% | Maintain |
| % of mothers attending a facility within 7 days of delivery | Percentage point increase | 15% | 10% | 5% | 5% | Maintain |
| % of women using long term contraceptives | Percentage point increase | 20% | 15% | 10% | Maintain above 71% | Maintain |
| % children under 1 year receiving Penta3 vaccine | Overall result | 50% | 65% | 75% | 80% + | Maintain |
| % children under 1 year receiving measles vaccine | Overall result | 50% | 65% | 75% | 80% + | Maintain |
| *Content of care indicators* |  |  |  |  |  |  |
| % ANC clients receiving IPT2 | Overall result | 80% | 80% | 80% | 80%+ | Maintain above 80% |
| % HIV+ ANC clients on ART | Overall result | 40% | 60% | 75% | 75%+ | Maintain |
| % of newborns receiving polio vaccine (OPV0) | Overall result | 60% | 75% | 80% | 80%+ | Maintain |
| *HMIS strengthening* |  |  |  |  |  |  |
| HMIS monthly reports correctly filled and submitted on time to CHMT | Overall result | 100% | 100% | 100% | 100% | 100% |

Notes to Table: +*85% or more. Antiretroviral therapy (ART), Antenatal care (ANC), Council Health Management Team (CHMT), Management Information System (HMIS), Human Immunodeficiency Virus (HIV), Intermittent Preventative Treatment (IPT2).

Source: Binyaruka *et al.* (2015), ﻿Ministry of Health and Social Welfare (2012) and Cassidy *et al.* (2021).

Table S1.2: Council Health Management Team and Regional Health Management Team performance indicators set during the pilot P4P programme in Tanzania.

| **CHMT/RHMT/Both** | **Indicator** | **Measure** |
| --- | --- | --- |
|  | *Coverage indicators* |  |
| Both | % of maternal and newborn deaths that are appropriately audited on time | Overall result |
|  | *Health system strengthening* |  |
| CHMT | % of facilities reporting stock-outs of either one or more of the tracer medicines in a specified period (< 8 days) | Overall result |
|  | *HMIS strengthening* |  |
| CHMT | % of facilities included in the HMIS monthly reports exported through DHIS to RHMT in timely manner | Overall result |
|  | *Management* |  |
| RHMT | Submission to MoHSW of a Semi-Annual Regional Health Profile report, based on DHIS | Overall result |
| CHMT | % of facilities receiving a copy of a Quarterly District Health Profile report, based on DHIS | Overall result |
|  | *Overall* |  |
| Both | Overall performance along P4P facility-based indicators | Overall result |

Notes to Table: Council Health Management Team (CHMT), District Health Information Software (DHIS), Health Management Information System (HMIS), Ministry of Health and Social Welfare (MoHSW), Regional Health Management Team (RHMT).

Source: ﻿Ministry of Health and Social Welfare (2012) and Cassidy *et al.* (2021).

*Supplementary File 2 – Detailed views of model sectors*

Detailed views of each model sector are given in this Supplementary File; **Population, Demand and Services, Facility Commodities**, **Facility Operations, Facility Funding and District Manager Operations**. Supplementary Files 3 and 4 provide further information on model equations and descriptions of data used.

N.B. The **Demand and Services** and **Facility Commodities** sectors are each presented here with two sub-sectors, describing functions related to the two different health services of interest in the model (percentage of women who received two doses of IPT during ANC and percentage of women who had a facility-based delivery). This was an artifact of model development (for ease of viewing and analysis), for all intents and purposes they can be considered ‘subsectors’ of a single model sector.

**Population sector**

Figure S2.2: Detailed model view of Population sector

**Demand and Services sector (ANC)**

Figure S2.3: Detailed model view of Demand and Services sector

**Demand and Services sector (Facility-based deliveries)**

Figure S2.4: Detailed model view of Demand and Services sector

**Facility Commodities sector (ANC)**

Figure S2.5: Detailed model view of Facility Commodities sector

**Facility Commodities sector (Facility-based deliveries)**

Figure S2.6: Detailed model view of Facility Commodities sector

**Facility Operations sector**

Figure S2.7: Detailed model view of Facility Operations sector

**Facility Funding sector**

Figure S2.8: Detailed model view of Facility Funding sector

**District Manager Operations sector**

Figure S2.9: Detailed model view of District Manager Operations sector

*Supplementary File 3 - Description of Model Equations*

**Model summary description**

Time horizon for model: 54 months

Time step for model: 1 month

Time units: Months

**Model equations and documentation**

*Population sector*

***Stocks***

Neonates(t) = Neonates(t - dt) + (NeonatesBeingBorn - GrowtoInfant - DyingNeonates) * dt

INIT Neonates = 4,740 {people}

DOCUMENT: Number of neonates (infants who are less than a month old).

INFLOWS:

NeonatesBeingBorn = Number of newly pregnant women {people/month}

OUTFLOWS:

GrowtoInfant = Neonates/DurationInfant {people/month}

DyingNeonates = Neonates*NeonateMortalityRate {people/month}

Infants(t) = Infants(t - dt) + (GrowtoInfant - DyingInfants - GrowtoPreschool) * dt

INIT Infants = 49,500 {people}

DOCUMENT: Number of infants over 1 month and up to 1 year.

INFLOWS:

GrowtoInfant = Neonates/DurationInfant {people/month}

OUTFLOWS:

DyingInfants = Infants*InfantMortalityRate {people/month}

GrowtoPreschool = Infants/DurationPreschool {people/month}

Preschoolers(t) = Preschoolers(t - dt) + (GrowtoPreschool - DyingPreschoolers - GrowtoChild) * dt

INIT Infants = 175,000 {people}

DOCUMENT: Number of preschoolers over 1 year and up to 5 years.

INFLOWS:

GrowtoPreschool = Infants/DurationPreschool {people/month}

OUTFLOWS:

DyingPreschoolers = Preschoolers*PreschoolerMortalityRate {people/month}

GrowtoChild = Preschoolers/DurationChild {people/month}

Children(t) = Children(t - dt) + (GrowtoChild - GrowtoReproductiveAge - DyingChildren) * dt

INIT Children = 284,308 {people}

DOCUMENT: Number of children aged between 5 and up to 15 years.

INFLOWS:

GrowtoChild = Preschoolers /DurationChild {people/month}

OUTFLOWS:

GrowtoReproductiveAge = Children/DurationReprod {people/month}

DyingChildren = Children*ChildMortalityRate {people/month}

ReproductiveAgeAdults(t) = ReproductiveAgeAdults(t - dt) + (GrowtoReproductiveAge - Growabove50 - DyingReprodPeople) * dt

INIT ReproductiveAgeAdults = 509,934 {people}

DOCUMENT: This is reproductive population aged between 15 and up to 50 years.

INFLOWS:

GrowtoReproductiveAge = Children/DurationReprod {people/month}

OUTFLOWS:

Growabove50 = ReproductiveAgeAdults/DurationAdultAbove50 {people/month}

DyingReprodPeople = ReproductiveAgeAdults*ReprodAgeMortalityRate {people/month}

AdultsAbove50(t) = AdultsAbove50(t - dt) + (Growabove50 - DyingAbove50) * dt

INIT AdultsAbove50 = 150,094 {people}

DOCUMENT: Number of adults above 50 years.

INFLOWS:

Growabove50 = ReproductiveAgeAdults/DurationAdultAbove50 {people/month}

OUTFLOWS:

DyingAbove50 = AdultsAbove50*Above50MortalityRate {people/month}

***Auxiliary variables***

NeonateMotalityRate = 0.026/12 {1/month}

DOCUMENT: The rate at which neonates die per month.

InfantMortalityRate = 0.025/12 {1/month}

DOCUMENT: The rate at which infants die per month.

PreschoolerMortalityRate = 0.032/12 {1/month}

DOCUMENT: The rate at which children of preschool age die per month.

ChildMortalityRate = 0.0024/12 {1/month}

DOCUMENT: The rate at which children die per month.

ReprodAgeMortalityRate = 0.005/12 {1/month}

DOCUMENT: The rate at which people from reproductive age group die per month.

Above50MortalityRate = 0.0575/12 {1/month}

DOCUMENT: The rate at which people aged 50 and above die per month.

GeneralFertilityRate = 0.2148/12 {1/month}

DOCUMENT: This is the birth rate per 1000 people.

DurationInfant = 1 {month}

DOCUMENT: Time it takes a neonate to become an infant.

DurationPreschool = 11 {month}

DOCUMENT: Time it takes an infant to become a preschooler.

DurationChildren = 48 {month}

DOCUMENT: Duration it takes for a preschooler to become a child.

DurationReprod = 120 {month}

DOCUMENT: Duration it takes for a child to become reproductive.

DurationAdultAbove50 = 420 {month}

DOCUMENT: Duration it takes for an adult to stop being reproductive.

﻿FractionofReproductiveAgeAdultsFemale = 0.52 {unitless}

DOCUMENT: Fraction of reproductive age adults who are female per year.

Femalereproductiveadults = FractionofReproductiveAgeAdultsFemale * ReproductiveAgeAdults(t) {people}

DOCUMENT: Number of female reproductive age adults.

Numberofnewlypregnantwomen = GeneralFertilityRate * Femalereproductiveadults {people}

DOCUMENT: Number of newly pregnant women of reproductive age per month.

*Demand and Services (Antenatal Care) sector*

***Stocks***

ANC visit 1(t) = ANC visit 1(t - dt) + (Eligible ANC1 – DropoutANC1 - Treated 1 (IPT) – Not treated 1) * dt

INIT ANC visit 1 = 4750 {people}

DOCUMENT: Number of women eligible to attend first ANC visit. Either drop out, receive a dose of malaria treatment or do not receive treatment depending on provider readiness and dropout rate.

INFLOWS:

Eligible ANC1 = Number of newly pregnant women {people/month}

OUTFLOWS:

DropoutANC1 = ANC visit 1*Dropoutrate1 {people/month}

Treated 1 (IPT) = (ANC visit 1-DropoutANC1)*Provider readiness (IPT) {people/month}

Not treated 1 = (ANC visit 1-DropoutANC1)*(1- Provider readiness (IPT)) {people/month}

ANC visit 2(t) = ANC visit 2(t - dt) + (Treated 1 (IPT) – DropoutANC2 – Treated 2 (IPT2) - Not treated 2 (IPT)) * dt

INIT ANC visit 2 = 470 {people}

DOCUMENT: Number of women eligible to attend second ANC visit (having received their first dose of malaria treatment in previous visit). Either drop out, receive a second dose of malaria treatment or receive no treatment depending on provider readiness and dropout rate.

INFLOWS:

Treated 1 (IPT) = (ANC visit 1-DropoutANC1)*Provider readiness (IPT) {people/month}

OUTFLOWS:

DropoutANC2 = ANC visit 2 * Dropoutrate2 {people/month}

Treated 2 (IPT2) = (ANC visit 2-DropoutANC2)*Provider readiness (IPT) {people/month}

Not treated 2 (IPT) = (ANC visit 2-DropoutANC2)*(1- Provider readiness (IPT)) {people/month}

ANC visit 2a(t) = ANC visit 2a(t - dt) + (Not treated 1 – DropoutANC2a – Treated 2 (IPT) - Not treated 2) * dt

INIT ANC visit 2a = 4190 {people}

DOCUMENT: Number of women eligible to attend second ANC visit (who did not receive their first dose of malaria treatment in previous visit). Either drop out, receive a first dose of malaria treatment or receive no treatment depending on provider readiness and dropout rate.

INFLOWS:

Not treated 1 = (ANC visit 1-DropoutANC1)*(1- Provider readiness (IPT)) {people/month}

OUTFLOWS:

DropoutANC2a = ANC visit 2a * Dropoutrate2 {people/month}

Treated 2 (IPT) = (ANC visit 2a-DropoutANC2a)*Provider readiness (IPT) {people/month}

Not treated 2 = (ANC visit 2a-DropoutANC2a)*(1- Provider readiness (IPT)) {people/month}

ANC visit 3(t) = ANC visit 3(t - dt) + (Treated 2 (IPT2) - DropoutANC3 – Treated 3) * dt

INIT ANC visit 3 = 156 {people}

DOCUMENT: Number of women eligible to attend third ANC visit (who have had their second dose of IPT). Either drop out or become eligible to attend ANC visit 4 depending on dropout rate.

INFLOWS:

Treated 2 (IPT2) = (ANC visit 2-DropoutANC2)*Provider readiness (IPT) {people/month}

OUTFLOWS:

DropoutANC3 = ANC visit 3 * Dropoutrate3 {people/month}

Treated 3 = (ANC visit 3-DropoutANC3) {people/month}

ANC visit 3a(t) = ANC visit 3a(t - dt) + (Not treated 2 – DropoutANC3a – Treated 3 (IPT) - Not treated 3) * dt

INIT ANC visit 3a = 2700 {people}

DOCUMENT: Number of women eligible to attend third ANC visit (who did not receive their first dose of malaria treatment in previous visit). Either drop out, receive a first dose of malaria treatment or receive no treatment depending on provider readiness and dropout rate.

INFLOWS:

Not treated 2 = (ANC visit 2a-DropoutANC2a)*(1- Provider readiness (IPT)) {people/month}

OUTFLOWS:

DropoutANC3a = ANC visit 3a * Dropoutrate3 {people/month}

Treated 3 (IPT) = (ANC visit 3a-DropoutANC3a)*Provider readiness (IPT) {people/month}

Not treated 3 = (ANC visit 3a-DropoutANC3a)*(1- Provider readiness (IPT)) {people/month}

ANC visit 3b(t) = ANC visit 3b(t - dt) + (Not treated 2 (IPT) + Treated 2 (IPT) – DropoutANC3b – Treated 3 (IPT2) - Not treated 3 (IPT) ) * dt

INIT ANC visit 3b = 1650 {people}

DOCUMENT: Number of women eligible to attend third ANC visit (who did receive their second dose of malaria treatment in previous visit). Either drop out, receive a second dose of malaria treatment or do not receive a second dose of treatment depending on provider readiness and dropout rate.

INFLOWS:

Not treated 2 (IPT) = (ANC visit 2-DropoutANC2)*(1- Provider readiness (IPT)) {people/month}

Treated 2 (IPT) = (ANC visit 2a-DropoutANC2a)*Provider readiness (IPT) {people/month}

OUTFLOWS:

DropoutANC3b = ANC visit 3b * Dropoutrate3 {people/month}

Treated 3 (IPT2) = (ANC visit 3b-DropoutANC3b)*Provider readiness (IPT) {people/month}

Not treated 3 (IPT) = (ANC visit 3b-DropoutANC3b)*(1- Provider readiness (IPT)) {people/month}

ANC visit 4(t) = ANC visit 4(t - dt) + (Treated 3 – DropoutANC4 – Treated 4) * dt

INIT ANC visit 4 = 1000 {people}

DOCUMENT: Number of women eligible to attend forth ANC visit (who have had their second dose of IPT). Either drop out or become eligible to attend ANC visit 5.

INFLOWS:

Treated 3 = (ANC visit 3-DropoutANC3) {people/month}

OUTFLOWS:

DropoutANC4 = ANC visit 4 * Dropoutrate4 {people/month}

Treated 4 = (ANC visit 4 – DropoutANC4) {people/month}

ANC visit 4a(t) = ANC visit 4a(t - dt) + (Not treated 3 – DropoutANC4a – Treated 4 (IPT) - Not treated 4) * dt

INIT ANC visit 4a = 740 {people}

DOCUMENT: Number of women eligible to attend fourth ANC visit (who did not receive their first dose of malaria treatment in previous visit). Either drop out, receive a first dose of malaria treatment or receive no treatment depending on provider readiness and dropout rate.

INFLOWS:

Not treated 3 = (ANC visit 3a-DropoutANC3a)*(1- Provider readiness (IPT)) {people/month}

OUTFLOWS:

DropoutANC4a = ANC visit 4a * Dropoutrate4 {people/month}

Treated 4 (IPT) = (ANC visit 4a-DropoutANC4a)*Provider readiness (IPT) {people/month}

Not treated 4 = (ANC visit 4a-DropoutANC4a)*(1- Provider readiness (IPT)) {people/month}

ANC visit 4b(t) = ANC visit 4b(t - dt) + (Not treated 3 (IPT) + Treated 3 (IPT) – DropoutANC4b – Treated 4 (IPT2) - Not treated 4 (IPT) ) * dt

INIT ANC visit 4b = 2170 {people}

DOCUMENT: Number of women eligible to attend fourth ANC visit (who received their first dose of malaria treatment in previous visit). Either drop out, receive a second dose of malaria treatment or do not receive a second dose of treatment depending on provider readiness and dropout rate.

INFLOWS:

Not treated 3 (IPT) = (ANC visit 3b-DropoutANC3b)*(1- Provider readiness (IPT)) {people/month}

Treated 3 (IPT) = (ANC visit 3a-DropoutANC3a)*Provider readiness (IPT) {people/month}

OUTFLOWS:

DropoutANC4b = ANC visit 4b * Dropoutrate4

Treated 4 (IPT2) = (ANC visit 4b-DropoutANC4b)*Provider readiness (IPT) {people/month}

Not treated 4 (IPT) = (ANC visit 4b-DropoutANC4b)*(1- Provider readiness (IPT)) {people/month}

ANC visit 4c(t) = ANC visit 4c(t - dt) + (Treated 3 (IPT2) – DropoutANC4c – Treated 4c) * dt

INIT ANC visit 4c = 235 {people}

DOCUMENT: Number of women eligible to attend fourth ANC visit (who did receive their second dose of malaria treatment in previous visit). Either drop out or become eligible to attend ANC visit 5.

INFLOWS:

Treated 3 (IPT2) = (ANC visit 3b-DropoutANC3b)*Provider readiness (IPT) {people/month}

OUTFLOWS:

DropoutANC4c = ANC visit 4c * Dropoutrate4

Treated 4c = (ANC visit 4c-DropoutANC4c) {people/month}

***Auxiliary variables***

Dropoutrate1 = GRAPH(TIME{people/month})

(1, 0.0163), (54, 0.0146)

DOCUMENT: Fraction of pregnant women who do not attend one ANC appointment.

Dropoutrate2 = GRAPH(TIME{ people/month })

(1, 0.0237), (54, 0.026)

DOCUMENT: Fraction of pregnant women who do not attend a second ANC appointment.

Dropoutrate3 = GRAPH(TIME{ people/month })

(1, 0.0705), (54, 0.0646)

DOCUMENT: Fraction of pregnant women who do not attend a third ANC appointment.

Dropoutrate4 = GRAPH(TIME{ people/month })

(1, 0.2455), (54, 0.2876)

DOCUMENT: Fraction of pregnant women who do not attend a fourth ANC appointment.

﻿

Maximum potential patients (First dose) =

(ANC visit 1-(ANC visit 1*Dropoutrate1))+

(ANC visit 2a-(ANC visit 2a*Dropoutrate2))+

(ANC visit 3a-(ANC visit 3a*Dropoutrate3))+

(ANC visit 4a-(ANC visit 4a*Dropoutrate4)) {people}

DOCUMENT: Maximum number of patients who present for treatment (first dose IPT)

Maximum potential patients (Second dose) =

(ANC visit 2-(ANC visit 2*Dropoutrate2))+

(ANC visit 3b-(ANC visit 3b*Dropoutrate3))+

(ANC visit 4b-(ANC visit 4b*Dropoutrate4)) {people}

DOCUMENT: Maximum number of patients who present for treatment (second dose IPT)

Maximum potential patients (IPT) =

Maximum potential patients (First dose)+Maximum potential patients (Second dose) {people}

DOCUMENT: Maximum number of patients who present for treatment (first and second dose IPT)

Percentage of women receive IPT =

IF TIME <= 4

THEN

IF TIME <= 3

THEN

IF TIME <=2

THEN

IF TIME <= 1

THEN

("Treated 4 (IPT)"+

Feed in initial attendance data[Treated 3 IPT, 4]+

Feed in initial attendance data[Treated 2 IPT, 3]+

Feed in initial attendance data[Treated 1 IPT, 2])/

Feed in initial attendance data[Attend ANC1,1]

ELSE

("Treated 4 (IPT)"+

HISTORY("Treated 3 (IPT)", TIME-1)+

Feed in initial attendance data[Treated 2 IPT, 4]+

Feed in initial attendance data[Treated 1 IPT, 3])/

Feed in initial attendance data[Attend ANC1,2]

ELSE

("Treated 4 (IPT)"+

HISTORY("Treated 3 (IPT)", TIME-1)+

HISTORY("Treated 2 (IPT)", TIME-2)+

Feed in initial attendance data[Treated 1 IPT, 4])/

Feed in initial attendance data[Attend ANC1,3]

ELSE

("Treated 4 (IPT)"+

HISTORY("Treated 3 (IPT)", TIME-1)+

HISTORY("Treated 2 (IPT)", TIME-2)+

HISTORY("Treated 1 (IPT)", TIME-3))/

Feed in initial attendance data[Attend ANC1,4]

ELSE

("Treated 4 (IPT)"+

HISTORY("Treated 3 (IPT)", TIME-1)+

HISTORY("Treated 2 (IPT)", TIME-2)+

HISTORY("Treated 1 (IPT)", TIME-3))/

HISTORY(Attend ANC1, TIME-4) {unitless}

DOCUMENT: Percentage of women who receive 1^st^ dose of IPT (of those who present for treatment). Patients have four opportunities to receive a first dose of IPT; this variable computes the percentage who receive their first dose for each cohort of ANC patients that passes through the model, once all opportunities for treatment have passed (at visit 4).

Percentage of women receive IPT2 =

IF TIME <= 4

THEN

IF TIME <= 3

THEN

IF TIME <=2

THEN

IF TIME <= 1

THEN

("Treated 4 (IPT2)"+

Feed in initial attendance data[Treated 2 IPT2,3]+

Feed in initial attendance data[Treated 3 IPT2,4])/

Feed in initial attendance data[Attend ANC1,1]

ELSE

("Treated 4 (IPT2)"+

Feed in initial attendance data[Treated 2 IPT2,4]+

HISTORY("Treated 3 (IPT2)", TIME-1))/

Feed in initial attendance data[Attend ANC1,2]

ELSE

("Treated 4 (IPT2)"+

HISTORY("Treated 2 (IPT2)", TIME-2)+

HISTORY("Treated 3 (IPT2)", TIME-1))/

Feed in initial attendance data[Attend ANC1,3]

ELSE

("Treated 4 (IPT2)"+

HISTORY("Treated 2 (IPT2)", TIME-2)+

HISTORY("Treated 3 (IPT2)", TIME-1))/

Feed in initial attendance data[Attend ANC1,4]

ELSE

("Treated 4 (IPT2)"+

HISTORY("Treated 2 (IPT2)", TIME-2)+

HISTORY("Treated 3 (IPT2)", TIME-1))/

HISTORY(Attend ANC1, TIME-4) {unitless}

DOCUMENT: Percentage of women who receive 2^nd^ dose of IPT (of those who present for treatment). Patients who receive a first dose of IPT have up to three opportunities to receive a second dose of IPT; this variable computes the percentage who receive their second dose for each cohort of ANC patients that passes through the model, once all opportunities for treatment have passed (at visit 3).

Attend ANC1 = ANC visit 1-(ANC visit 1*Dropoutrate1) {people/month}

DOCUMENT: Number attending ANC visit 1.

Drugs used =

Treated 1 (IPT)+

Treated 2 (IPT)+

Treated 3 (IPT)+

Treated 4 (IPT)+

Treated 2 (IPT2)+

Treated 3 (IPT2)+

Treated 4 (IPT2) {people/month}

DOCUMENT: Number of drugs used in treatment for IPT.

No attending ANC1 = ("Treated 1 (IPT)"+Not treated 1)/HISTORY(ANC visit 1, TIME-1) {per month}

DOCUMENT: Number of patients attending 1 ANC visit.

No attending ANC2 = ("Treated 2 (IPT)"+"Treated 2 (IPT2)"+"Not treated 2 (IPT)"+Not treated 2)/

HISTORY(ANC visit 1, TIME-2) {per month}

DOCUMENT: Number of patients attending 2 ANC visit.

No attending ANC3 = (Treated 3+"Treated 3 (IPT2)"+"Not treated 3 (IPT)"+"Treated 3 (IPT)"+Not treated 3)/

HISTORY(ANC visit 1, TIME-3) {per month}

DOCUMENT: Number of patients attending 3 ANC visit.

No attending ANC4 =

(Not treated 4+"Treated 4 (IPT)"+"Not treated 4 (IPT)"+"Treated 4 (IPT2)"+Treated 4c+Treated 4)/

HISTORY(ANC visit 1, TIME-4) {per month}

DOCUMENT: Number of patients attending 4 ANC visit.

*Facility Commodities (Drugs), Specifically IPT Drugs sector*

***Stocks***

Order of IPT drugs(t) = Order of IPT drugs(t – dt) + (IPT drugs needed –IPT drugs ordered) * dt

INIT Order of drugs = 14,600 {drugs}

DOCUMENT: Tracking the number of drugs used quarterly, this is then used to calculate the drugs ordered by the facility from the Medical Stores Department. Orders are made quarterly basis, stock is wiped every three months as orders are sent.

INFLOWS:

IPT drugs needed = Maximum potential patients (IPT) {drugs/month}

OUTFLOWS:

IPT drugs ordered = ﻿PULSE(Order of IPT drugs, 3, 3){drugs/month}

Stock of IPT drugs(t) = Stock of IPT drugs(t – dt) + (Replenishment of IPT drugs – Depletion of IPT drugs) * dt

INIT Stock of IPT drugs = 16,000 {drugs}

DOCUMENT: The stock of drugs available at the facility. The number of drugs reduces on a monthly basis based on services rendered and is topped up on a quarterly basis from MSD and monthly basis where facilities use own funds to purchase drugs.

INFLOWS:

Replenishment of drugs = ﻿MSD provision of IPT drugs to facility + Facility purchase of IPT drugs {drugs/month}

OUTFLOWS:

Depletion of IPT drugs = ﻿ IPT drugs used{drugs/month}

***Auxiliary variables***

MSD provision of IPT drugs to facility = ﻿IPT drugs ordered * MSD provision of IPT ordered {drugs/month}

DOCUMENT: Number of IPT drugs ordered that are then supplied by the MSD.

MSD provision of IPT ordered = {drugs}

GRAPH(TIME{drugs/month})

(1, 0.33), (18, 0.45), (54, 0.45)

DOCUMENT: Percentage of drugs ordered that are supplied by the MSD.

Decision to buy IPT drugs outside MSD = {unitless}

IF Stock of IPT drugs/Maximum potential patients (IPT) < 1

THEN 1

ELSE 0

DOCUMENT: Facility decision on whether to buy IPT drugs outside MSD. If Stock of IPT drugs/Maximum potential patients (IPT) falls below 1 i.e. stocks fall below what is required for service delivery, facility held funding can be used to purchase drugs if available.

Facility purchase of IPT drugs = {unitless}

IF Decision to buy IPT drugs outside MSD = 1

THEN

(Facility held funds available)*Maximum potential patients (IPT)

ELSE

0

DOCUMENT: Use of facility held funding to purchase drugs. If a decision is made on using facility held funds to purchase drugs in ‘Decision to buy IPT drugs outside MSD’, facility held funds are used to purchase drugs.

*Facility Operations sector*

***Stocks***

Number of health worker at health facility (% filled)(t)= Number of health worker at health facility (% filled)(t-dt) + (Change in staffing level) * dt

INIT Number of health worker at health facility (% filled) = 0.3991 {unitless}

DOCUMENT: Percentage of positions filled at health facilities.

BIFLOW = {per month}

IF "Number of health worker at health facility (% filled)" >= 1

THEN

-(Average attrition rate*"Number of health worker at health facility (% filled)")

ELSE (Hiring rate*"Number of health worker at health facility (% filled)")-(Average attrition rate*"Number of health worker at health facility (% filled)")

Knowledge of health workers (IPT)(t) = Knowledge of health workers (IPT)(t-dt) + (Change in knowledge (IPT)) * dt

INIT Knowledge of health workers (IPT) = 0.742 {unitless}

DOCUMENT: Percentage of time health workers are prescribing IPT during ANC visits, 0 (not prescribing at all) to 1 (prescribing to all patients).

BIFLOW = {per month}

(Perform supervision visit*(Effect of supervision on knowledge[1,ROUND(10*"Knowledge of health workers (IPT)")])+(P4P training*"Knowledge of health workers (IPT)"))

-

(Degradation of knowledge rate)

Trust in programme(t) = Trust in programme(t-dt) + (Change in trust) * dt

INIT 0.8 {unitless}

DOCUMENT: Trust in P4P changes over time in response to timing of incentive payments.

BIFLOW = {per month}

IF Trust in programme <1

THEN

Effect of incentives on trust-(Effect of delays in payment on trust["Effect of delays on trust (mode)",TIME])*Delay?

ELSE

MIN(0, (Effect of incentives on trust-(Effect of delays in payment on trust["Effect of delays on trust (mode)",TIME])*Delay?))

***Auxiliary variables***

Effect of supervision on knowledge = TABLE[Supervision on knowledge, Current knowledge] {unitless}

DOCUMENT = Table function, see table at end of section. Effect of supervision on knowledge, depending on current knowledge of provider.

Effect of incentives on trust = {unitless}

IF "Staff incentives (time corrected)">0

THEN Effect of incentive on trust (change)

ELSE 0

DOCUMENT = How payment of incentives changes health worker trust in P4P over time.

Effect of incentive on trust (change)= 0.05

DOCUMENT = Change in trust (either negative or positive) based on incentive payment.

Effect of delays in payment on trust = TABLE[Delays on trust mode, Delay P4P payment] {unitless}

DOCUMENT: Table function, see table at end of section. Effect of delays in payment on trust of health workers in P4P programme.

Effect of delays on trust (mode) =

3

DOCUMENT: There are three possible effects of payment delays on trust (1,2,3).

Hiring rate = 0.12/12 {unitless}

DOCUMENT: The rate at which health worker positions are filled.

Average attrition rate = 0.057/12 {unitless}

DOCUMENT = The rate at which health workers are leaving their positions.

Degradation of knowledge rate = (0.057/12)/10 {unitless}

DOCUMENT = Decrease in health worker skill over time. Related to attrition rate. Average attrition rate/10

P4P training = {unitless}

IF TIME = 1

THEN 0.05*P4P switch

ELSE 0

DOCUMENT = Initial increase in health worker knowledge attributed to training at start of programme. Only viable at time 1.

Provider readiness (IPT) = {unitless}

MIN(Availability of IPT drugs, MEAN("Number of health worker at health facility (% filled)", Health worker motivation to exert effort towards incentivised services, Availability of IPT drugs, "Knowledge of health workers (IPT)"))

DOCUMENT = Provider readiness to deliver services to patients, range 0 (low provider readiness) to 1 (max provider readiness, can treat all patients who present for care.

Health worker motivation = {unitless}

IF P4P switch = 1

THEN MEAN("Availability of drugs (IPT and labour)", "Number of health worker at health facility (% filled)", "District manager supervision (quality)", Trust in programme)

ELSE

MEAN("Availability of drugs (IPT and labour)", "Number of health worker at health facility (% filled)", "District manager supervision (quality)")

DOCUMENT = Health worker motivation to deliver incentivized services, range 0 (low motivation) to 1 (high motivation).

Staff incentives (time corrected) = {unitless}

DELAY("Staff incentives (delays?)", 1)

DOCUMENT = There is a lag between incentive payment issued and payment received (payment is issued month 6, received by month 9 etc.).

***Auxiliary variables - table functions***

Effect of supervision on knowledge = TABLE[Supervision on knowledge, Current knowledge] {unitless}

DOCUMENT = Table function, see table at end of section. Effect of supervision on knowledge, depending on current knowledge of provider.

|  | **1** | **2** | **3** | **4** | **5** | **6** | **7** | **8** | **9** | **10** |
| --- | --- | --- | --- | --- | --- | --- | --- | --- | --- | --- |
| **1** | 0.1 | 0.1 | 0.1 | 0.1 | 0.2 | 0.2 | 0.025 | 0.025 | 0.025 | 0 |

Effect of delays in payment on trust = TABLE[Delays on trust mode, Delay P4P payment] {unitless}

DOCUMENT: Table function, see table at end of section. Effect of delays in payment on trust of health workers in P4P programme.

|  | **1** | **2** | **3** | **4** | **5** | **6** | **7** | **8** | **9** | **10** | **11** | **12** | **13** | **14** | **15** | **16** | **17** | **18** | **19** | **20** |
| --- | --- | --- | --- | --- | --- | --- | --- | --- | --- | --- | --- | --- | --- | --- | --- | --- | --- | --- | --- | --- |
| **1** | 0 | 0 | 0 | 0 | 0 | 0 | 0 | 0 | 0 | 0 | 0 | 0 | 0 | 0 | 0 | 0 | 0 | 0 | 0 | 0 |
| **2** | 0 | 0 | 0 | 0 | 0 | 0 | 0 | 0.05 | 0.05 | 0.05 | 0.05 | 0 | 0 | 0.05 | 0.05 | 0.05 | 0 | 0 | 0 | 0.05 |
| **3** | 0 | 0 | 0 | 0 | 0 | 0 | 0 | 0 | 0 | 0 | 0.05 | 0 | 0 | 0 | 0 | 0 | 0 | 0 | 0 | 0 |

| **21** | **22** | **23** | **24** | **25** | **26** | **27** | **28** | **29** | **30** | **31** | **32** | **33** | **34** | **35** | **36** | **37** | **38** | **39** | **40** |
| --- | --- | --- | --- | --- | --- | --- | --- | --- | --- | --- | --- | --- | --- | --- | --- | --- | --- | --- | --- |
| 0 | 0 | 0 | 0 | 0 | 0 | 0 | 0 | 0 | 0 | 0 | 0 | 0 | 0 | 0 | 0 | 0 | 0 | 0 | 0 |
| 0 | 0 | 0 | 0 | 0 | 0 | 0 | 0 | 0 | 0 | 0 | 0.05 | 0 | 0 | 0 | 0 | 0 | 0.05 | 0.05 | 0.05 |
| 0 | 0 | 0 | 0 | 0 | 0 | 0 | 0 | 0 | 0 | 0 | 0 | 0 | 0 | 0 | 0 | 0 | 0 | 0 | 0 |

| **41** | **42** | **43** | **44** | **45** | **46** | **47** | **48** | **49** | **50** | **51** | **52** | **53** | **54** |
| --- | --- | --- | --- | --- | --- | --- | --- | --- | --- | --- | --- | --- | --- |
| 0 | 0 | 0 | 0 | 0 | 0 | 0 | 0 | 0 | 0 | 0 | 0 | 0 | 0 |
| 0 | 0 | 0 | 0.05 | 0.05 | 0.05 | 0.05 | 0.05 | 0.05 | 0.05 | 0.05 | 0.05 | 0.05 | 0.05 |
| 0 | 0 | 0 | 0 | 0 | 0 | 0.05 | 0.05 | 0.05 | 0.05 | 0.05 | 0.05 | 0.05 | 0.05 |

*District Manager Operations sector*

***Stocks***

District manager motivation to support facilities = District manager motivation to support facilities(t-dt) + Change in motivation * dt

INIT: 0.6 Scale of 0 to 1, 0 (low motivation), 1 (high motivation). {unitless}

DOCUMENT: District manager motivation to supervise facilities.

BIFLOW: {per month}

IF District manager motivation to support facilities > Baseline motivation

THEN

IF District manager motivation to support facilities < 1

THEN

(Effect of initial knowledge of P4P on motivation+Effect of incentive on motivation-Effect of delays in payment on trust["Effect of delays on trust (mode)",TIME])*P4P switch

ELSE

MIN(0, (Effect of initial knowledge of P4P on motivation+Effect of incentive on motivation-Effect of delays in payment on trust["Effect of delays on trust (mode)",TIME])*P4P switch)

ELSE

MAX(0, (Effect of initial knowledge of P4P on motivation+Effect of incentive on motivation-Effect of delays in payment on trust["Effect of delays on trust (mode)",TIME])*P4P switch)

***Auxiliary variables***

District manager supervision (quality) = {unitless}

MEAN(District manager motivation to support facilities, "Skill level or knowledge of district manager (supervision)", "District budget/resources")

DOCUMENT: Quality of district manager supervision, dependent on motivation, knowledge and budget. Scale of 0 to 1, 0 (low quality), 1 (high quality).

Skill level or knowledge of district manager (supervision) = 0.8 {unitless}

DOCUMENT: Skill level or knowledge of managers regarding supervision. Scale of 0 to 1, 0 (low skill), 1 (high skill).

District budget/resources = 0.5 {unitless}

DOCUMENT: Percentage of allocated supervision budget that is available. Scale of 0 to 1, 0 (no funds), 1 (all allocated funds available).

Perform supervision visit = {unitless}

IF District budget/resources>= 0.5

THEN PULSE(District manager supervision (quality), 4, 4)

ELSE 0

DOCUMENT: This auxiliary variable passes the quality of supervision variable to the change in health worker knowledge stock every 4 months (if there are enough resources available for visits).

Baseline motivation = 0.6 {unitless}

DOCUMENT: Minimum level for motivation.

Effect of incentive on motivation = {unitless}

IF "Total incentive awarded to CHMT (time corrected)">0

THEN

Effect of incentive amount on motivation

ELSE

0

DOCUMENT: Effect of incentive payment on motivation to supervise.

Effect of incentive amount on motivation = 0.05 {unitless}

DOCUMENT: Increase (or decrease) in motivation induced by incentive payments.

Effect of initial knowledge of P4P on motivation = {unitless}

IF TIME = 1

THEN 0.1

ELSE 0

DOCUMENT: Effect of initial knowledge of P4P on motivation

Total incentive awarded to CHMT = {unitless}

DELAY((Eligible for facility performance incentive?+Eligible for stock out of RCH drugs incentive?)/2, 1)

DOCUMENT: Total incentive amount awarded to CHMT.

Total incentive awarded to CHMT (time corrected) = {unitless}

DELAY("Total incentive awarded to CHMT (delays?)", 1)

DOCUMENT: There is a lag between incentive payment issued and payment received (payment is issued month 6, received by month 9 etc.).

Total incentive awarded to CHMT (delays?) = {unitless}

IF TIME>6 AND Delay? = 1

THEN

IF TIME>6 AND TIME<13

THEN DELAY(Total incentive awarded to CHMT, 4)

ELSE

IF TIME>12 AND TIME<19

THEN DELAY(Total incentive awarded to CHMT, 3)

ELSE

IF TIME>18 AND TIME<25

THEN DELAY(Total incentive awarded to CHMT, 1)

ELSE

IF TIME>24 AND TIME<31

THEN Total incentive awarded to CHMT

ELSE

IF TIME>30 AND TIME<37

THEN DELAY(Total incentive awarded to CHMT, 1)

ELSE

IF TIME>36 AND TIME<43

THEN DELAY(Total incentive awarded to CHMT, 3)

ELSE

IF TIME>42

THEN 0

ELSE 999

ELSE Total incentive awarded to CHMT

DOCUMENT: Implements a delay in payment if the delay switch is turned ‘on’.

Eligible for facility performance incentive? = {unitless}

IF (Total incentive awarded/"Target to receive CHMT incentive (Overall result)") >= Lower performance band

THEN

(IF (Total incentive awarded/"Target to receive CHMT incentive (Overall result)") >= Upper performance band

THEN 1

ELSE 0.5)

ELSE 0

DOCUMENT: Checking whether CHMT is eligible to receive incentive payment for this target.

Target to receive CHMT incentive (Overall result) = 0.8 {unitless}

DOCUMENT: Facility performance needed to induce CHMT payment for this target.

Eligible for stock out of RCH drugs incentive? = {unitless}

(Stock out of IPT?*Stock out of FBD?)

DOCUMENT: Checking if CHMT are eligible to receive incentive payment for this target.

Stock out of FBD? = {unitless}

IF Reporting cycle = 1

THEN

IF

Stock of FBD drugs = 0 OR

HISTORY(Stock of FBD drugs, TIME-1) = 0 OR

HISTORY(Stock of FBD drugs, TIME-2) = 0 OR

HISTORY(Stock of FBD drugs, TIME-3) = 0 OR

HISTORY(Stock of FBD drugs, TIME-4) = 0 OR

HISTORY(Stock of FBD drugs, TIME-5) = 0

THEN 0

ELSE 1

ELSE 0

DOCUMENT: Checking if facilities have had a stock out in the last 6 months.

Stock out of IPT? = {unitless}

IF Reporting cycle = 1

THEN

IF

Stock of IPT drugs = 0 OR

HISTORY(Stock of IPT drugs, TIME-1) = 0 OR

HISTORY(Stock of IPT drugs, TIME-2) = 0 OR

HISTORY(Stock of IPT drugs, TIME-3) = 0 OR

HISTORY(Stock of IPT drugs, TIME-4) = 0 OR

HISTORY(Stock of IPT drugs, TIME-5) = 0

THEN 0

ELSE 1

ELSE 0

DOCUMENT: Checking if facilities have had a stock out in the last 6 months.

*Demand and Services (Facility-Based Deliveries) sector*

***Stocks***

Community awareness(t) = Community awareness(t-dt) + (Change in awareness) * dt

INIT Community awareness = 0.85 {unitless}

DOCUMENT = Represents awareness of maternal and child health and healthcare in the community. Awareness is improved through outreach, attendance at ANC and facility-based deliveries in community and depleted by belief in myths and decay of awareness (in absence of activities that promote awareness).

BIFLOW = {per month}

IF Community awareness < 1

THEN (

((Fraction of women attending at least one ANC*Effect ANC awareness)

+(Ability to perform outreach*Effect outreach awareness)

+(Percentage of women who seek facility-based delivery*Effect FBD awareness))

-(Effect myths literacy awareness*Decay awareness))*Community awareness

ELSE

-(Effect myths literacy awareness*Decay awareness)*Community awareness

Number of facility-based deliveries(t) = Number of facility-based deliveries(t-dt) + (Seeking FBD – FBD) * dt

INIT Number of facility-based deliveries = 3670 {people}

DOCUMENT = Number of facility based deliveries.

INFLOWS:

Seeking FBD = Number of Newly Pregnant Women*Fraction who seek FBD {people/month}

OUTFLOWS:

FBD = Number of facility-based deliveries {people/month}

***Auxiliary variables***

Percentage of women who seek facility-based delivery = {unitless}

0.18*"Perceived quality of facility/services"+

0.273*Community awareness+

0.273*Effect ANC on FBD+

0.273*Distance to facility

DOCUMENT = Fraction of women who seek facility-based delivery.

Patient-provider interaction = Health worker motivation to exert effort towards incentivised services {unitless}

DOCUMENT: Interaction between health workers and patients (tied to health worker motivation). Scale of 0 (low quality interaction), 1 (high quality interaction).

Perceived quality of facility/services =

MEAN("Patient-provider interaction", "Availability of drugs (IPT and labour)") {unitless}

DOCUMENT: Patient perceived quality of services, 0 (low quality) to 1 (high quality).

Distance to facility =

GRAPH(TIME{unitless })

(1, 0.948), (18, 0.983), (54, 0.957)

DOCUMENT: Percentage of women that do not perceive distance to being a barrier for facility-based delivery.

Effect ANC on FBD = {unitless}

((Dropoutrate2*Prob FBD ANC 1)+

(Dropoutrate3*Prob FBD ANC 2)+

(Dropoutrate4*Prob FBD ANC 3)+

((1-Dropoutrate1-Dropoutrate2-Dropoutrate3-Dropoutrate4)*Prob FBD ANC 4))*1.2

DOCUMENT: Effect of number of ANC visits on facility-based deliveries.

Prob FBD ANC 1= EXP(0.47)/1+EXP(0.47) =61.54 {Unitless}

DOCUMENT = Probability of facility-based delivery for those who attend one ANC visit.

Prob FBD ANC 2= EXP(0.74)/1+EXP(0.74) = 67.70 {unitless}

DOCUMENT = Probability of facility-based delivery for those who attend two ANC visit.

Prob FBD ANC 3= EXP(1.13)/1+EXP(1.13) = 75.58 {unitless}

DOCUMENT = Probability of facility-based delivery for those who attend three ANC visit.

Prob FBD ANC 4= EXP(1.52)/1+EXP(1.52) = 82.05{unitless}

DOCUMENT = Probability of facility-based delivery for those who attend four + ANC visits.

FBD drugs used = Number of facility-based deliveries {drugs}

DOCUMENT: Number of facility-based deliveries and facility-based delivery drugs used per month.

Fraction of women attending at least one ANC = 1 – Dropoutrate1 {unitless}

DOCUMENT: The fraction of women who attend at least 1 ANC appointment.

Effect ANC awareness = 0.001{unitless}

DOCUMENT: Effect of ANC on awareness of maternal and child health and healthcare in the community.

Literacy = TIME [(2011, 0.730), (2012, 0.781), (2013, 0.780), (2014, 0.780), (2015, 0.779)] {unitless}

DOCUMENT: Literacy rates for Tanzania adults (15+).

Effect literacy awareness = 0.001 {unitless}

DOCUMENT: Effect of literacy on awareness of maternal and child health and healthcare.

Belief in myths = 0.01{unitless}

DOCUMENT: Percentage of the population that believes in myths.

Effect myths = 0.001{unitless}

DOCUMENT: Effect of myths on awareness of maternal and child health and healthcare in the community.

Effect myths literacy awareness = (Belief in myths*Effect myths)*(Literacy*Effect literacy awareness) {unitless}

DOCUMENT: Effect of myths and literacy rate on awareness of maternal and child health and healthcare in the community.

Decay awareness = 0.015{unitless}

DOCUMENT: Decay of awareness in community over time.

Ability to perform outreach = {unitless}

MEAN(Number of health worker at health facility % filled), Health worker motivation to exert effort towards incentivised services)

DOCUMENT = Ability of health workers to perform outreach activities, dependent on number of health workers at facility and motivation.

Effect outreach awareness = 0.001{unitless}

DOCUMENT = Effect of outreach on awareness of maternal and child health and healthcare in the community.

Effect FBD awareness = 0.001{unitless}

DOCUMENT = Effect of FBD on awareness of maternal and child health and healthcare in the community.

*Facility Commodities (Drugs), Specifically Labour Drugs sector*

***Stocks***

Order of FBD drugs(t) = Order of FBD drugs(t – dt) + (FBD drugs needed –FBD drugs ordered) * dt

INIT Order of FBD drugs = 3,720 {drugs}

DOCUMENT: Tracking the number of drugs used quarterly, this is then used to calculate the drugs ordered by the facility from the Medical Stores Department. Orders are made on quarterly basis, stock is wiped every three months as orders are sent.

INFLOWS:

FBD drugs needed = Seeking FBD {drugs/month}

OUTFLOWS:

FBD drugs ordered = ﻿PULSE(Order of FBD drugs, 3, 3){drugs/month}

Stock of FBD drugs(t) = Stock of FBD drugs(t – dt) + (Replenishment of FBD drugs – Depletion of FBD drugs) * dt

INIT Stock of FBD drugs = 7,600 {drugs}

DOCUMENT: The stock of drugs available at the facility. The number of drugs reduces on a monthly basis based on services rendered and is topped up on a quarterly basis from MSD and monthly basis where facilities use own funds to purchase drugs.

INFLOWS:

Replenishment of FBD drugs = ﻿MSD provision of FBD drugs to facility + Facility purchase of FBD drugs {drugs/month}

OUTFLOWS:

Depletion of FBD drugs = ﻿ FBD drugs used{drugs/month}

***Auxiliary variables***

MSD provision of FBD drugs to facility = ﻿FBD drugs ordered * MSD provision of FBD drugs ordered {drugs}

DOCUMENT: Number of FBD drugs ordered that are then supplied by the MSD.

MSD provision of FBD drugs ordered =

GRAPH(TIME{drugs/month})

(1, 0.63), (18, 0.63), (54, 0.63)

DOCUMENT: Percentage of drugs ordered that are supplied by the MSD.

Decision to buy FBD drugs outside MSD =

IF Stock of FBD drugs/Number of facility-based deliveries < 1

THEN 1

ELSE 0

DOCUMENT: Facility decision on whether to buy FBD drugs outside MSD. If Stock of FBD drugs/Number of facility-based deliveries falls below 1 i.e. stocks fall below what is required for service delivery, facility held funding can be used to purchase drugs if available.

Facility purchase of FBD drugs =

IF Decision to buy FBD drugs outside MSD = 1

THEN

(Facility held funds available)* Number of facility-based deliveries

ELSE

0

DOCUMENT: Use of facility held funding to purchase drugs. If a decision is made on using facility held funds to purchase drugs in ‘Decision to buy FBD drugs outside MSD’, facility held funds are used to purchase drugs.

*Facility Funding sector*

***Stocks***

Incentives used for drugs(t) = Incentives used for drugs(t - dt) + (Incoming incentive payment – Funds used) * dt

INIT Incentives used for drugs = 0 {unitless}

DOCUMENT: Incentives available to purchase drugs where needed.

INFLOW:

Incoming incentive payment = IF TIME>6 AND Delay? = 1 {per month}

THEN

IF TIME>6 AND TIME<13

THEN DELAY(Operation incentives*0.6, 4)

ELSE

IF TIME>12 AND TIME<19

THEN DELAY(Operation incentives*0.6, 3)

ELSE

IF TIME>18 AND TIME<25

THEN DELAY(Operation incentives*0.6, 1)

ELSE

IF TIME>24 AND TIME<31

THEN Operation incentives*0.6

ELSE

IF TIME>30 AND TIME<37

THEN DELAY(Operation incentives*0.6, 1)

ELSE

IF TIME>36 AND TIME<43

THEN DELAY(Operation incentives*0.6, 3)

ELSE

IF TIME>42

THEN 0

ELSE 999

ELSE Operation incentives*0.6

OUTFLOW:

Funds used = IF TIME>5 AND Delay? = 1 {per month}

THEN

IF TIME = 17 OR TIME= 22 OR TIME = 26 OR TIME =

31 OR TIME = 38 OR TIME = 46

THEN HISTORY(Incoming incentive payment, TIME-6)

ELSE 0

ELSE

PULSE(Incentives used for drugs, 13, 6)

***Auxiliary variables***

Actual timing of payment =

((DELAY(Incoming incentive payment, 2)*2)/(1-Use of incentives))*P4P switch {unitless}

DOCUMENT: This number is used to represent the actual timing of incentive payments when there are delays, which is represented graphically in the model interface.

Alternative facility held funding available = 0.1 {unitless}

DOCUMENT: Facilities can buy 10% of the monthly drugs they need with alterative facility held funding.

Cycle 1 check =

IF TIME = 6

THEN

IF FBD performance change >= 0.85

THEN FBD performance change

ELSE 0

ELSE

FBD performance change

DOCUMENT: There is no historical performance preceding first cycle of facility-based deliveries; if cycle 1 performance is 0.85 or better, they achieve the target. If it is less, we assume they did not achieve the target for cycle 1.

Delay? = 1 {unitless}

DOCUMENT: A switch that turns payment delays on and off.

Eligible for FBD incentive? =

IF "Fraction who seek FBD (6 month period)" >= 0.85

THEN

1*P4P switch

ELSE

IF

(Cycle 1 check/(("Target to receive facility incentive (increase in % points)"[1,ROUND((Previous cycle performance*100)+1)]))) >= Lower performance band

THEN

(IF

(Cycle 1 check/"Target to receive facility incentive (increase in % points)"[1,ROUND((Previous cycle performance*100)+1)]) >= Upper performance band

THEN 1*P4P switch

ELSE 0.5*P4P switch)

ELSE 0

DOCUMENT: Calculating how much incentive payment should be paid for FBD target.

Eligible for IPT2 incentive? = {unitless}

IF ("Percentage of women receive IPT2 (6 month period)"/"Target to receive facility incentive (Overall result)") >= Lower performance band

THEN

(IF ("Percentage of women receive IPT2 (6 month period)"/"Target to receive facility incentive (Overall result)") >= Upper performance band

THEN 1*P4P switch

ELSE 0.5*P4P switch)

ELSE 0

DOCUMENT: Calculating how much incentive payment should be paid for IPT target.

Facility held funds available = (Incentives used for drugs*P4P switch)+Alternative facility held funding available

DOCUMENT: Facility held funds available to purchase drugs.

FBD performance change = {unitless}

IF Previous cycle performance >= 0.85

THEN

IF "Percentage of women who seek facility-based delivery (6 month period)" >= 0.85

THEN 1

ELSE 0

ELSE

IF ("Percentage of women who seek facility-based delivery (6 month period)"-Previous cycle performance)<=0

THEN 0

ELSE ("Percentage of women who seek facility-based delivery (6 month period)"-Previous cycle performance)

DOCUMENT: The change in performance between cycles for the FBD target.

FBD lower performance band = {unitless}

IF TIME = 6

THEN NAN

ELSE

IF Reporting cycle = 1

THEN

IF "Percentage of women who seek facility-based delivery (6 month period)" >= 0.85

THEN 0.85

ELSE

IF "Percentage of women who seek facility-based delivery (6 month period)">= 0.85 AND Previous cycle performance >= 0.85

THEN 0.85

ELSE

Previous cycle performance+

("Target to receive facility incentive (increase in % points)"[1,ROUND((Previous cycle performance*100)+1)]*0.75)

ELSE PREVIOUS(SELF, NAN)

DOCUMENT: This number is used to represent the lower performance band for this target on the performance graph presented on the model interface.

FBD upper performance band = {unitless}

IF TIME = 6

THEN NAN

ELSE

IF Reporting cycle = 1

THEN

IF "Percentage of women who seek facility-based delivery (6 month period)" >= 0.85

THEN 0.85

ELSE

IF "Percentage of women who seek facility-based delivery (6 month period)" >= 0.85 AND Previous cycle performance >= 0.85

THEN 0.85

ELSE

Previous cycle performance+

"Target to receive facility incentive (increase in % points)"[1,ROUND((Previous cycle performance*100)+1)]

ELSE PREVIOUS(SELF, NAN)

DOCUMENT: This number is used to represent the upper performance band for this target on the performance graph presented on the model interface.

"Fraction who seek FBD (6 month period)" = {unitless}

IF Reporting cycle = 1

THEN

MEAN(

Percentage of women who seek facility-based delivery,

HISTORY(Percentage of women who seek facility-based delivery, TIME-1),

HISTORY(Percentage of women who seek facility-based delivery, TIME-2),

HISTORY(Percentage of women who seek facility-based delivery, TIME-3),

HISTORY(Percentage of women who seek facility-based delivery, TIME-4),

HISTORY(Percentage of women who seek facility-based delivery, TIME-5)

)

ELSE 0

DOCUMENT: Calculating performance for FBD.

IPT lower performance band = {unitless}

"Target to receive facility incentive (Overall result)"*Lower performance band

DOCUMENT: This number is used to represent the lower performance band for this target on the performance graph presented on the model interface.

IPT upper performance band = {unitless}

"Target to receive facility incentive (Overall result)"*Upper performance band

DOCUMENT: This number is used to represent the upper performance band for this target on the performance graph presented on the model interface.

Lower performance band = 0.75 {unitless}

DOCUMENT: Facilities need to achieve at least 75% of the target to receive 50% of the payment for that target.

Operation incentives = DELAY(Total incentive awarded*(1-Use of incentives), 1)

DOCUMENT: Percentage of incentive used for facility operations (drugs).

P4P switch = 0 {unitless}

DOCUMENT: The switch to turn P4P programme on (1) or off (0).

"Percentage of women receive IPT2 (6 month period)" = {unitless}

IF Reporting cycle = 1

THEN

MEAN(

Percentage of women receive IPT2,

HISTORY(Percentage of women receive IPT2, TIME-1),

HISTORY(Percentage of women receive IPT2, TIME-2),

HISTORY(Percentage of women receive IPT2, TIME-3),

HISTORY(Percentage of women receive IPT2, TIME-4),

HISTORY(Percentage of women receive IPT2, TIME-5)

)

ELSE 0

DOCUMENT: Performance of facility on the IPT2 target.

Planned timing of payment = {unitless}

DELAY(Total incentive awarded, 3)*P4P switch

DOCUMENT: This number is used to represent the planned timing of incentive payments, which is represented graphically in the model interface.

Previous cycle performance = {unitless}

IF TIME = 6

THEN 0

ELSE

HISTORY("Percentage of women who seek facility-based delivery (6 month period)", TIME-6)

DOCUMENT: Previous cycles FBD performance.

Reporting cycle = PULSE(1, 6, 6) {unitless}

DOCUMENT: Facilities report performance data every six months in order to be eligible for incentive payments.

Staff incentives = DELAY(Total incentive awarded*(Use of incentives), 1) {unitless}

DOCUMENT: Incentives are paid three months after incentive amount is agreed.

Staff incentives (delays?) = {unitless}

IF TIME>6 AND Delay? = 1

THEN

IF TIME>6 AND TIME<13

THEN DELAY(Staff incentives, 4)

ELSE

IF TIME>12 AND TIME<19

THEN DELAY(Staff incentives, 3)

ELSE

IF TIME>18 AND TIME<25

THEN DELAY(Staff incentives, 1)

ELSE

IF TIME>24 AND TIME<31

THEN Staff incentives

ELSE

IF TIME>30 AND TIME<37

THEN DELAY(Staff incentives, 1)

ELSE

IF TIME>36 AND TIME<43

THEN DELAY(Staff incentives, 3)

ELSE

IF TIME>42

THEN 0

ELSE 999

ELSE Staff incentives

DOCUMENT: If ‘delay’ is switched on, delays in payment are made.

"Target to receive facility incentive (increase in % points)" = TABLE[Increase in % required, Previous cycle] {unitless}

DOCUMENT: Table function, see table at end of section. Outputs the change in performance needed to achieve incentive payment.

"Target to receive facility incentive (Overall result)" = 0.8 {unitless}

DOCUMENT: Target for IPT2 delivery to achieve incentive payment.

Total incentive awarded = (Eligible for IPT2 incentive?*0.5)+(Eligible for FBD incentive?*0.5) {unitless}

DOCUMENT: Total incentive awarded based on performance of IPT2 and FBD.

Upper performance band = 1 {unitless}

DOCUMENT: Facilities need to achieve 100% of the target to receive 100% of the payment for that target.

Use of incentives = 0.75 {unitless}

DOCUMENT: 75% of the incentive payment goes to health workers, 25% for facility operations.

***Auxiliary variables - table functions***

"Target to receive facility incentive (increase in % points)" = TABLE[Increase in % required, Previous cycle] {unitless}

DOCUMENT: Table function. Outputs the change in performance needed to achieve incentive payment.

|  | 1 | 2 | 3 | 4 | 5 | 6 | 7 | 8 | 9 | 10 | 11 | 12 | 13 | 14 | 15 | 16 | 17 | 18 | 19 | 20 |
| --- | --- | --- | --- | --- | --- | --- | --- | --- | --- | --- | --- | --- | --- | --- | --- | --- | --- | --- | --- | --- |
| 1 | 0.15 | 0.15 | 0.15 | 0.15 | 0.15 | 0.15 | 0.15 | 0.15 | 0.15 | 0.15 | 0.15 | 0.15 | 0.15 | 0.15 | 0.15 | 0.15 | 0.15 | 0.15 | 0.15 | 0.1 |

| 21 | 22 | 23 | 24 | 25 | 26 | 27 | 28 | 29 | 30 | 31 | 32 | 33 | 34 | 35 | 36 | 37 | 38 | 39 | 40 | 41 |
| --- | --- | --- | --- | --- | --- | --- | --- | --- | --- | --- | --- | --- | --- | --- | --- | --- | --- | --- | --- | --- |
| 0.1 | 0.1 | 0.1 | 0.1 | 0.1 | 0.1 | 0.1 | 0.1 | 0.1 | 0.1 | 0.1 | 0.1 | 0.1 | 0.1 | 0.1 | 0.1 | 0.1 | 0.1 | 0.1 | 0.05 | 0.05 |

| 42 | 43 | 44 | 45 | 46 | 47 | 48 | 49 | 50 | 51 | 52 | 53 | 54 | 55 | 56 | 57 | 58 | 59 | 60 | 61 | 62 |
| --- | --- | --- | --- | --- | --- | --- | --- | --- | --- | --- | --- | --- | --- | --- | --- | --- | --- | --- | --- | --- |
| 0.05 | 0.05 | 0.05 | 0.05 | 0.05 | 0.05 | 0.05 | 0.05 | 0.05 | 0.05 | 0.05 | 0.05 | 0.05 | 0.05 | 0.05 | 0.05 | 0.05 | 0.05 | 0.05 | 0.05 | 0.05 |

| 63 | 64 | 65 | 66 | 67 | 68 | 69 | 70 | 71 | 72 | 73 | 74 | 75 | 76 | 77 | 78 | 79 | 80 | 81 | 82 | 83 |
| --- | --- | --- | --- | --- | --- | --- | --- | --- | --- | --- | --- | --- | --- | --- | --- | --- | --- | --- | --- | --- |
| 0.05 | 0.05 | 0.05 | 0.05 | 0.05 | 0.05 | 0.05 | 0.05 | 0.05 | 0.05 | 0.05 | 0.05 | 0.05 | 0.05 | 0.05 | 0.05 | 0.05 | 0.05 | 0.05 | 0.05 | 0.05 |

| 63 | 64 | 65 | 66 | 67 | 68 | 69 | 70 | 71 | 72 | 73 | 74 | 75 | 76 | 77 | 78 | 79 | 80 | 81 | 82 | 83 |
| --- | --- | --- | --- | --- | --- | --- | --- | --- | --- | --- | --- | --- | --- | --- | --- | --- | --- | --- | --- | --- |
| 0.05 | 0.05 | 0.05 | 0.05 | 0.05 | 0.05 | 0.05 | 0.05 | 0.05 | 0.05 | 0.05 | 0.05 | 0.05 | 0.05 | 0.05 | 0.05 | 0.05 | 0.05 | 0.05 | 0.05 | 0.05 |

| 84 | 85 | 86 | 87 | 88 | 89 | 90 | 91 | 92 | 93 | 94 | 95 | 96 | 97 | 98 | 99 | 100 | 101 |
| --- | --- | --- | --- | --- | --- | --- | --- | --- | --- | --- | --- | --- | --- | --- | --- | --- | --- |
| 0.05 | 0.000001 | 0.000001 | 0.000001 | 0.000001 | 0.000001 | 0.000001 | 0.000001 | 0.000001 | 0.000001 | 0.000001 | 0.000001 | 0.000001 | 0.000001 | 0.000001 | 0.000001 | 0.000001 | 0.000001 |

*Supplementary File 4 - Description of Parameters Document*

This Supplementary file provides a description of model parameters and data used for the following model sectors; **Population, Demand and Services, Facility Commodities**, **Facility Operations, Facility Funding and District Manager Operations**. Supplementary Files 2 and 3 provide further information on model sectors and model equations.

N.B. The **Demand and Services** and **Facility Commodities** sectors are each presented here with two sub-sectors, describing data related to the model equations for two different health services of interest in the model (percentage of women who received two doses of IPT during ANC and percentage of women who had a facility-based delivery). This was an artifact of model development (for ease of viewing and analysis), for all intents and purposes they can be considered ‘subsectors’ of a single model sector.

*Population sector*

Table S4.1: Population sector variable descriptions and data sources

|  | **Variable Name** | **Description** | **Initial Value** | **Units** | **Reference** |
| --- | --- | --- | --- | --- | --- |
| 1 | Neonates | Number of neonates (infants who are less than a month old) in Pwani region. Estimated at a 1/12 of the population of infants. (32830/12) = 2736  0 ≤ x < 1/12 | 2,736  Changed to 4740 during calibration | People | National Bureau of Statistics (2013). |
| 2 | Infants | Number of infants over 1 month and up to 1 year in Pwani region.. The number of infants below 1 year were 32830. (32830-neonates(2736)=30094)  1/12 ≤ x < 1 | 30,094  Changed to 49500 during calibration | People | National Bureau of Statistics (2013). |
| 3 | Preschoolers | Number of children aged  1 ≤ x < 5 in Pwani region. | 154,332  Changed to 175000 during calibration | People | National Bureau of Statistics (2013). |
| 4 | Children | Number of children aged  5 ≤ x < 15 in Pwani region. | 284,308 | People | National Bureau of Statistics (2013). |
| 5 | ReproductiveAgeAdults | Number of adults of reproductive age  15 ≤ x < 50 in Pwani region. | 509,934 | People | National Bureau of Statistics (2013). |
| 6 | AdultsAbove50 | Number of adults above 50 years.  x ≥ 50 in Pwani region. | 150,094 | People | National Bureau of Statistics (2013). |
|  | DurationInfant | The duration it takes a neonate to become an infant. 1 month | 1 | Months | -- |
|  | DurationPreschool | The duration it takes an infant to become a preschooler. 11 months | 11 | Months | -- |
| 7 | DurationChild | The duration it takes for a preschooler to become a child.  4 years  4*12 = 48 | 48 | Months | -- |
| 8 | DurationReprod | Duration it takes for a child to become reproductive.  10 years  10*12 = 120 | 120 | Months | -- |
| 9 | DurationAdultAbove50 | The duration it takes for an adult to stop being reproductive.  35 years  35*12 = 420 | 420 | Months | -- |
| 10 | FractionofReproductiveAgeAdultsFemale | The fraction of reproductive age adults who are female. | 0.52 | Unitless | National Bureau of Statistics (2013). |
| 11 | NeonateMortalityRate | The rate at which neonates (less than one month) die per year.  For Tanzania - five years preceding the survey (2006-2010) neonatal mortality is 26 deaths per 1000 live births. | 0.026/12 | 1/month | National Bureau of Statistics (2011). |
| 12 | InfantMortalityRate | The rate at which infants (1 month to 1 year) die per year.  For Tanzania - five years preceding the survey (2006-2010) infant mortality (1 month to 1 year) is 25 deaths per 1000 live births. | 0.025/12 | 1/month | National Bureau of Statistics (2011). |
|  | PreschoolMortRate | The rate at which preschoolers die per year.  For Tanzania - five years preceding the survey (2006-2010) preschool mortality (1 year to 5 years) is 32 deaths per 1000 live births. | 0.032/12 | 1/month | National Bureau of Statistics (2011). |
| 13 | ChildMortRate | The rate at which children die per year.  Number of children dying between age 5 and 14 for Tanzania mainland is 2.4 per 1000 persons. | 0.0024/12 | 1/month | National Bureau of Statistics (2015). |
| 14 | ReprodAgeMortality | The rate at which people from reproductive age group die per year.  Age-specific mortality rates for women and men age 15-49 based on the survivorship of sisters and brothers of survey respondents, for the ten-year period preceding the survey, Tanzania 2010.  Women (age standardized rate) 5.1 deaths per 1000 years of exposure)  Men (age standardized rate) 5 deaths per 1000 years of exposure) | 0.005/12 | 1/month | National Bureau of Statistics (2011). |
| 15 | Above50MortalityRate | The rate at which people over the age of 50 die per year.  Tanzania mainland mortality 60+ is 57.5 per 1000 persons. | 0.0575/12 | 1/month | National Bureau of Statistics (2015). |
| 16 | GeneralFertilityRate | Number of live births per 1000 women of reproductive age.  Tanzania (2010 DHS) general fertility rate three years preceding the survey, number of live births per 1000 women of reproductive age (15-49), 189 per 1000 women.  NOTES DURING CALIBRATION: Changing 0.189/12 to 0.2148/12 | 0.2148/12 | 1/month | National Bureau of Statistics (2011). |
| 17 | Projected population growth rates for Tanzania mainland | Using the raw data, numbers for each age category reported, percentage change per year:  2013 3.2  2014 3.2  2015 3.2  2016 3.2  2017 3.2  2018 3.1  2019 3.1  2020 3.1  2021 3.1  2022 3.1  2023 3.1  2024 3.1  2025 3.0 | Used raw data percentage change to calibrate growth in population | 1/year | National Bureau of Statistics (2018). |

*Demand and Services (Antenatal Care) sector*

Table S4.2: Demand and Services sector variable descriptions and data sources

|  | **Variable Name** | **Description** | **Initial Value** | **Units** | **Reference** |
| --- | --- | --- | --- | --- | --- |
| 1 | ANC visit 1 | Number of women eligible to attend first ANC visit. | Calibrated based on incoming flow.  4,750 | People | -- |
| 2 | ANC visit 2 | Number of women eligible to attend second ANC visit (who have had their first dose of IPT) | Calibrated based on incoming flow.  470 | People | -- |
| 3 | ANC visit 2a | Number of women eligible to attend second ANC visit (who have not had first dose of IPT) | Calibrated based on incoming flow.  4190 | People | -- |
| 4 | ANC visit 3 | Number of women eligible to attend third ANC visit (who have had their second dose of IPT) | Calibrated based on incoming flow.  156 | People | -- |
| 5 | ANC visit 3a | Number of women eligible to attend third ANC visit (who have not had first dose of IPT) | Calibrated based on incoming flow.  2700 | People | -- |
| 6 | ANC visit 3b | Number of women eligible to attend third ANC visit (who have had their first dose of IPT). | Calibrated based on incoming flow.  1650 | People | -- |
| 7 | ANC visit 4 | Number of women eligible to attend fourth ANC visit (who have had their second dose of IPT) | Calibrated based on incoming flow.  1000 | People | -- |
| 8 | ANC visit 4a | Number of women eligible to attend fourth ANC visit (who have not had first dose of IPT) | Calibrated based on incoming flow.  740 | People | -- |
| 9 | ANC visit 4b | Number of women eligible to attend fourth ANC visit (who have had their first dose of IPT). | Calibrated based on incoming flow.  2170 | People | -- |
| 10 | ANC visit 4c | Number of women eligible to attend fourth ANC visit (who have had their second dose of IPT). | Calibrated based on incoming flow.  235 | People | -- |
| 11 | Dropoutrate1 | The fraction of pregnant women who do not attend 1 ANC visit. | TIME  [(2011, 0.0163), (2015, 0.0146)] | People/  Month | Pwani IHI/LSHTM impact evaluation, (2011, 2015) |
| 12 | Dropoutrate2 | Fraction of pregnant women who do not attend a second ANC appointment. | TIME  [(2011, 0.0237), (2015, 0.026)] | People/  Month | Pwani IHI/LSHTM impact evaluation, (2011, 2015) |
| 13 | Dropoutrate3 | Fraction of pregnant women who do not attend a third ANC appointment. | TIME  [(2011, 0.0705), (2015, 0.0646)] | People/  Month | Pwani IHI/LSHTM impact evaluation, (2011, 2015) |
| 14 | Dropoutrate4 | Fraction of pregnant women who do not attend a fourth ANC appointment. | TIME  [(2011, 0.2455), (2015, 0.2876)] | People/  Month | Pwani IHI/LSHTM impact evaluation, (2011, 2015) |

*Demand and Services variables (Facility-based delivery care)*

Table S4.3: Demand and Services sector variable descriptions and data sources

|  | **Variable Name** | **Description** | **Initial Value** | **Units** | **Reference** |
| --- | --- | --- | --- | --- | --- |
| 1 | Community awareness | Knowledge and awareness of maternal and child health and healthcare (including facility-based deliveries). | 0.85  Assumed. | Unitless | -- |
| 2 | Number of facility-based deliveries | Number of facility-based deliveries. | Calibrated based on incoming flow.  3670 | People | -- |
| 3 | Distance to facility | Percentage of women that do not perceive distance to being a barrier for facility-based delivery. | TIME  [(2011, 0.948), (2013, 0.983), (2015, 0.957)] | Unitless | Pwani IHI/LSHTM impact evaluation, (2011, 2013, 2015) |
| 4 | Belief in myths | Percentage of the female population that believes in myths. Model assumes 1% of the female population believes in myths. | 0.01 | Unitless | Semwanga *et al.* (2016) |
| 5 | Literacy | Percent of adult population (15+) that are literate. | TIME  [(2011, 0.730), (2012, 0.781), (2013, 0.780), (2014, 0.780), (2015, 0.779)]  Assumed 2011, 2013 and 2014 based on adjacent years data (2010, 2012, 2015). | Unitless | UNESCO Institute for Statistics |
| 6 | Decay awareness | Decay in awareness in community over time. | Assumed  0.015 | Unitless | -- |
| 7 | Prob FBD ANC 1 | Probability of facility-based delivery for those who attend one ANC visit. | EXP(0.47)/  1+EXP(0.47)=  61.54 | Unitless | Ensor *et al.* (2014) |
| 8 | Prob FBD ANC 2 | Probability of facility-based delivery for those who attend two ANC visit. | EXP(0.74)/  1+EXP(0.74)=  67.70 | Unitless | Ensor *et al.* (2014) |
| 9 | Prob FBD ANC 3 | Probability of facility-based delivery for those who attend three ANC visit. | EXP(1.13)/  1+EXP(1.13)=  75.58 | Unitless | Ensor *et al.* (2014) |
| 10 | Prob FBD ANC 4+ | Probability of facility-based delivery for those who attend four + ANC visits. | EXP(1.52)/  1+EXP(1.52)=  82.05 | Unitless | Ensor *et al.* (2014) |

*Facility Commodities variables, specifically for antenatal care*

Table S4.4: Facility Commodities sector variable descriptions and data sources

|  | **Variable Name** | **Description** | **Initial Value** | **Units** | **Reference** |
| --- | --- | --- | --- | --- | --- |
| 1 | Order of drugs | Number of drugs used quarterly (every four months). | Calibrated based on incoming flow.  14,600 | Drugs | -- |
| 2 | Stock of drugs | Stock of drugs available at the facility. | Calibrated based on incoming flow.  16,000 | Drugs | -- |
| 3 | MSD provision of IPT drugs ordered (%) | Percentage of drugs ordered that are then supplied by the MSD. | Original assumption:  0.5  Calibrated to percentage of women who receive IPT2.  GRAPH(TIME{drugs/month})  (1, 0.33), (18, 0.45), (54, 0.45) | Drugs/  Month | -- |

*Facility Commodities variables, specifically for facility-based delivery care*

Table S4.5: Facility Commodities sector variable descriptions and data sources

|  | **Variable Name** | **Description** | **Initial Value** | **Units** | **Reference** |
| --- | --- | --- | --- | --- | --- |
| 1 | Order of FBD drugs | Number of drugs used quarterly (every four months). | Calibrated based on incoming flow.  3,720 | Drugs | -- |
| 2 | Stock of FBD drugs | Stock of drugs available at the facility. | Calibrated based on incoming flow.  7,600 | Drugs | -- |
| 3 | MSD provision of drugs ordered (%) | Percentage of drugs ordered that are then supplied by the MSD. | Original assumption:  0.5  Calibrated to fraction of women who seek facility-based delivery.  GRAPH(TIME{drugs/month})  (1, 0.63), (18, 0.63), (54, 0.63) | Drugs/  Month | -- |

*Facility Operations variables*

Table S4.6: Facility Operations sector variable descriptions and data sources

|  | **Variable Name** | **Description** | **Initial Value** | **Units** | **Reference** |
| --- | --- | --- | --- | --- | --- |
| 1 | Number of health worker at health facility (% filled) | Percentage of positions filled at health facilities. (0 no positions filled, 1 all positions filled) | Used the following data to calibrate hiring rate:  2011 – 39.91  2012 – 42.37  2013 – 48.25  2014 – 47.00  Assumed 2011, based on adjacent years 2009 (35.00) and 2012 (42.37) data. | Unitless | Ministry of Health and Social Welfare (2013)  Ministry of Health and Social Welfare (2014a) |
| 2 | Hiring rate | The rate at which health worker positions are filled from hiring | 2011 – 0.11  2012 – 0.09  2013 – 0.11  With calibration, 0.12/12 | Unitless | Ministry of Health and Social Welfare (2014b) |
| 4 | Average attrition rate | The rate at which health workers are leaving their positions. | 0.057 per year  0.057/12 | Unitless | Kurowski *et al.* (2007) |
| 5 | Skill level/knowledge of health workers (IPT2) | The skill level of health workers at facilities (percentage, 0 low skill, 1 high skill). | 0.742 | Unitless | Pwani IHI/LSHTM impact evaluation, (2011). |
| 6 | Effect of supervision on knowledge | Effect district manager supervision has on health worker knowledge to prescribe IPT during ANC. | Determined through qualitative interview with stakeholder.  There will be some improvement after even one visit. Depends on what knowledge level is like at facility already. Need reminders to keep up knowledge.  For those with high knowledge/skill, didn’t need several visits for improvement, single visit enough.  Table function (see page 11 of model equations document) | Unitless | -- |
| 6 | Degradation of skill rate | Decrease in health worker skill over time. Related to attrition rate.  Average attrition rate/10 | Assumed.  Attrition/10  (0.057/12)/10 | Unitless | -- |
| 7 | P4P training | Initial increase in health worker skill level/knowledge attributed to training at start of programme | Assumed.  0.05 | Unitless | -- |
| 8 | Effect of incentive on trust (change) | Change in trust (either negative or positive) based on payment of incentives. | Assumed. 0.05 | Unitless | -- |
| 9 | Effect of delays in payment on trust | Effect of delays in payment on trust, incremental increase or decrease. | Determined through qualitative interview with stakeholders.  Only severe delays in payment negatively impact trust (4+ months delay).  Table function (see page 11 of model equations document) | Unitless | -- |

*Facility Funding variables*

Table S4.7: Facility Funding sector variable descriptions and data sources

|  | **Variable Name** | **Description** | **Initial Value** | **Units** | **Reference** |
| --- | --- | --- | --- | --- | --- |
| 1 | P4P switch | Switch for turning P4P on (1) and off (0) in the model. | 0 | Unitless | -- |
| 2 | Use of incentives | 75% of the incentive payment goes to health workers, 25% for facility operations. | 0.75 | Unitless | Binyaruka *et al.* (2015) |
| 3 | Target to receive facility incentive (Overall result) | Target for IPT2 delivery to achieve incentive payment. | 0.8 | Unitless | Binyaruka *et al.* (2015) |
| 4 | Upper performance band | Facilities need to achieve 100% of the target to receive 100% of the payment for that target. | 1 | Unitless | Binyaruka *et al.* (2015) |
| 5 | Lower performance band | Facilities need to achieve at least 75% of the target to receive 50% of the payment for that target. | 0.75 | Unitless | Binyaruka *et al.* (2015) |
| 6 | Target to receive facility incentive (increase in % points) | Table function. Outputs the change in performance needed to achieve incentive payment. | Table function (see page 23 of model equations document) | Unitless | Binyaruka *et al.* (2015) |

*District Manager Operations variables*

Table S4.8: District Manager Operations sector variable descriptions and data sources

|  | **Variable Name** | **Description** | **Initial Value** | **Units** | **Reference** |
| --- | --- | --- | --- | --- | --- |
| 1 | Skill level or knowledge of district manager (supervision) | The skill or knowledge of district managers related to supervision activities (percentage, 0 low skill, 1 high skill). | 0.8 assumed. | Unitless | -- |
| 2 | District budget/resources | The percentage of the budget (allocated for supervision activities) that district managers can effectively use for supervision (percentage, 0 none of the budget, 1 all of the budget). | 0.5 assumed. | Unitless | -- |
| 3 | District manager motivation | Motivation of district level managers to perform supervision activities (percentage, 0 low motivation, 1 high motivation). | 0.6 assumed. | Unitless | -- |
| 4 | Effect of incentive amount on motivation | Increase (or decrease) in motivation induced by incentive payments. | 0.05 assumed. | Unitless | -- |
| 5 | Target to receive CHMT incentive (Overall result) | Facility performance needed to induce CHMT payment for this target. | 0.8 | Unitless | Ministry of Health and Social Welfare (2012) |
| 6 | Baseline motivation | Minimum level for motivation. | 0.6 assumed. | Unitless | -- |

*Supplementary File 5 – Model calibration graphs*

*Population sector*

The percentage population change was calculated for each year in the model (Figure S5.1) and compared to projected population growth rates for Tanzania (Supplementary File 4, Table S4.1). The initial values for each age category and fertility rate for adults of reproductive age were adjusted until a good fit for population change was achieved (original and calibrated initial values and rates are shown in Supplementary File 4, Table S4.1). The model output and data were also compared for population growth within each age category over time (Figures S5.2 – S5.7). The model output for neonate and infant population growth is a close fit (apart from the first year of data, 2012/13). The data for older age groups reads as quite volatile; without understanding the cause for the dramatic change year on year, it is difficult to replicate in the model. The model instead follows a general smoothed trend for these age categories.

Figure S5.1: Model calibration results for total population change each year. Source: National Bureau of Statistics (2013, 2018).

Figure S5.2: Model calibration results for number of neonates each year. Source: National Bureau of Statistics (2013, 2018).

Figure S5.3: Model calibration results for number of infants each year. Source: National Bureau of Statistics (2013, 2018).

Figure S5.4: Model calibration results for number of preschoolers each year. Source: National Bureau of Statistics (2013, 2018).

Figure S5.5: Model calibration results for number of children each year. Source: National Bureau of Statistics (2013, 2018).

Figure S5.6: Model calibration results for number of reproductive age adults each year. Source: National Bureau of Statistics (2013, 2018).

Figure S5.7: Model calibration results for number of adults above 50 each year. Source: National Bureau of Statistics (2013, 2018).

*Facility operations*

The percentage of positions filled at health facilities was output for each year in the model (Figure S5.8) and compared to data (Supplementary File 4, Table S4.6). The hiring rate for health worker positions was adjusted until a good fit for percentage of positions filled at health facilities was achieved (original and calibrated rates are shown in Supplementary File 4, Table S4.6). The model output is a good fit for the data aside from year 2013, where it is slightly underestimated.

Figure S5.8: Model calibration results for number of health workers at health facility (% filled) each year. Source: Ministry of Health and Social Welfare (2013, 2014a, 2014b).

*Facility funding*

The performance of targeted services (percentage of women who received two doses of IPT and percentage of women who had a facility-based delivery) when P4P is switched ‘on’ and ‘off’ in the model was output for ‘baseline’, ‘short term’ and long term’ time points and compared to data (Table S5.1) (Borghi *et al.* 2021). The improvement and decline between intervention and control sites was noted (Table S5.2) and used to calculate model calibration ‘aim’ for when P4P is switched ‘off’ in the model (Table S5.3), as initialisation of the model is the same regardless of whether P4P is then switched ‘on’ or ‘off’ in the model. The constant and uncertain (unable to extrapolate from data) variables ‘MSD provision of IPT ordered’ and ‘MSD provision of FBD drugs ordered’ were adjusted until a relatively good fit for both outputs were achieved (original and calibrated rates are shown in Supplementary File 4, Table S4.4 and Table S4.5).

Table S5.1: Performance at intervention and control sites for targeted services (at least two doses of IPT during ANC (%), institutional delivery rate (%)) at baseline, short term and long term evaluation. Source Borghi et al. 2021.

|  | **Intervention** | | | **Control** | | |
| --- | --- | --- | --- | --- | --- | --- |
|  | Baseline | Short term | Long term | Baseline | Short term | Long term |
| **Targeted services** | | | | | | |
| At least two doses of IPT during ANC (%) | 49.5 | 72.9 | 67.5 | 56.7 | 69.2 | 68.4 |
| Institutional delivery rate (%) | 84.7 | 89.2 | 92.2 | 86.8 | 83.1 | 89.4 |

Table S5.2: The difference in performance between baseline and short term, and short term and long term evaluations in the intervention and control sites.

|  | **Intervention** | | **Control** | |
| --- | --- | --- | --- | --- |
|  | Baseline/Short term | Short term/Long term | Baseline/Short term | Short term/Long term |
| **Targeted services** | |  |  |  |
| At least two doses of IPT during ANC (%) | 23.4 | -5.4 | 12.5 | -0.8 |
| Institutional delivery rate (%) | 4.5 | 3 | -3.7 | 6.3 |

Table S5.3: The goal for model calibration for both intervention and control model runs.

|  | **Intervention** | | | **Control** | | |
| --- | --- | --- | --- | --- | --- | --- |
|  | Baseline (aim) | Short term (aim) | Long term (aim) | Baseline (aim) | Short term (aim) | Long term (aim) |
| **Targeted services** | | | | | | |
| At least two doses of IPT during ANC (%) | 49.5 | 72.9 | 67.5 | 49.5 | 62.0 | 61.2 |
| Institutional delivery rate (%) | 84.7 | 89.2 | 92.2 | 84.7 | 81 | 87.3 |

The model output is a good fit for the percentage of women who received two doses of IPT when P4P is switched ‘on’ and ‘off’ in the model (Figures S5.9 and S5.10). The model overestimates the percentage of women who had a facility-based delivery when P4P is turned ‘off’ at the short term time point (Figure S5.11). In the real control evaluation site, facility-based deliveries actually decreased by 3.7% between baseline and the short-term evaluation (Borghi *et al.* 2021). Stakeholders were asked during validation interviews what might have caused this decrease in the control group, without reaching a definitive conclusion on the cause of the decrease which was in contrast to the national trend in facility-based deliveries during this period. The model also overestimates the percentage of women who had a facility-based delivery when P4P is turned ‘on’ at baseline and short-term periods. To ensure a more modest increase in the control and intervention groups, a reduced weight is assigned to the perceived quality of care input for variable equation percentage of women who seek facility-based delivery (see page 23 for equation), as this is the main pathway to impact for P4P on this service. The model slightly underestimates the percentage of women who had a facility-based delivery when P4P is turned ‘on’ at the long-term time point (Figure S5.12).

Figure S5.9: Model calibration results for percentage of women who receive at least two doses of IPT during ANC (%) in the control group.

Figure S5.10: Model calibration results for percentage of women who receive at least two doses of IPT during ANC (%) in the intervention group.

Figure S5.11: Model calibration results for percentage of women who seek a facility-based delivery (%) in the control group.

Figure S5.12: Model calibration results for percentage of women who seek a facility-based delivery (%) in the intervention group.

*Supplementary File 6 – Stakeholder interview validation tool*

***Interviewer:*** This series of interviews have been organised by researchers from Ifakara Health Institute (IHI) and the London School of Hygiene and Tropical Medicine (LSHTM). We are hoping to conduct interviews with experts, such as yourself, to validate a simulation model we have created of the Tanzania maternal and child health (MCH) system response to payment for performance (P4P).

***Interviewer:*** In an earlier interview (June/November/December 2020), we presented the system map we had developed using data collected during the P4P programme in Pwani that ran from 2011-2013. Using this system map, data collected during the programme, evidence from the literature and your feedback, we have now developed a computer model. In the model, we focus on the impact of P4P on the two programme targets where improvements were documented: provision of two doses of intermittent preventive treatment and facility-based deliveries.

***Interviewer:*** To ensure our model is accurately representing the behaviour of the Tanzanian health system and its response to the Pwani P4P scheme, we now require model output to be validated by experts. We also intend to further develop the model to examine health system response to more recent health financing programmes:

(i) The national Results-Based Finance programme (RBF, 2016-2020)

(ii) Direct Health Facility Financing programme (DHFF, 2019-Present)

***Interviewer:*** During this interview we will present and describe the key results from our simulation model related to the health system response to RBF (impact on health worker motivation, availability of medical commodities etc.). Using your knowledge of health system operation, your role will be to evaluate our results and determine their credibility. This crucial step in a series of model validation stages will provide confidence in the model, which will be used to develop policy recommendations for the implementation of results-based finance programmes in Tanzania and other settings.

***Interviewer:*** Just before we begin, I have received a hard copy of your consent form but I would just like to seek your verbal consent that you are happy to continue with the interview and you are happy for me to take written notes and an audio-recording of this session. This is only for our records and shared only with our research team. You can change your mind or stop the interview at any time.

OR

***Interviewer:*** Just before we begin, I am going to take verbal consent for the interview, I can then collect a hard copy of the consent form after the interview. I will now read through the informed consent form with you.

- You understand that your participation is voluntary.
- You understand that you can withdraw from the interview at any time.
- You understand that your feedback during the interview will be recorded via written notes and audio-recorded by a member of the research team (using either encrypted web-based software or a handheld recording device).
- You understand that your feedback collected in this interview will be kept confidential and be used for research purpose only. Also, you understand that your responses will be only shared with/by authorised individuals in the research team from LSHTM and IHI and any information included in the report, academic presentation or in published work will not be identified as the respondent.
- You confirm that you are happy for the research team to contact you in the future to ask follow up questions.
- You agree to take part in the above-named study.

***Interviewer:*** *Interviewer then proceeds with presentation and discussion of key model results and output periodically stopping to check interviewee understanding and to ask if any modifications should be made to the behaviour of the model to reflect their experience of the Tanzania programme.*

***Interviewer:*** At the moment, we are focused on Pwani P4P programme but also hope to model DHFF and RBF. We would like to ask you:

(i) if there other outputs you would like to see from the model

(ii) if there are things you would like to see varied in the model (to see impact on key outcomes).

(iii) do you think this model and types of simulation could be useful for decision making around the design of P4P programmes?

(iv) would you be happy for us to contact you with follow up questions?

***Interviewer:*** Thank you for your time today, this has been incredibly useful and it is very much appreciated by the COSMIC team.

*Model interface slides*


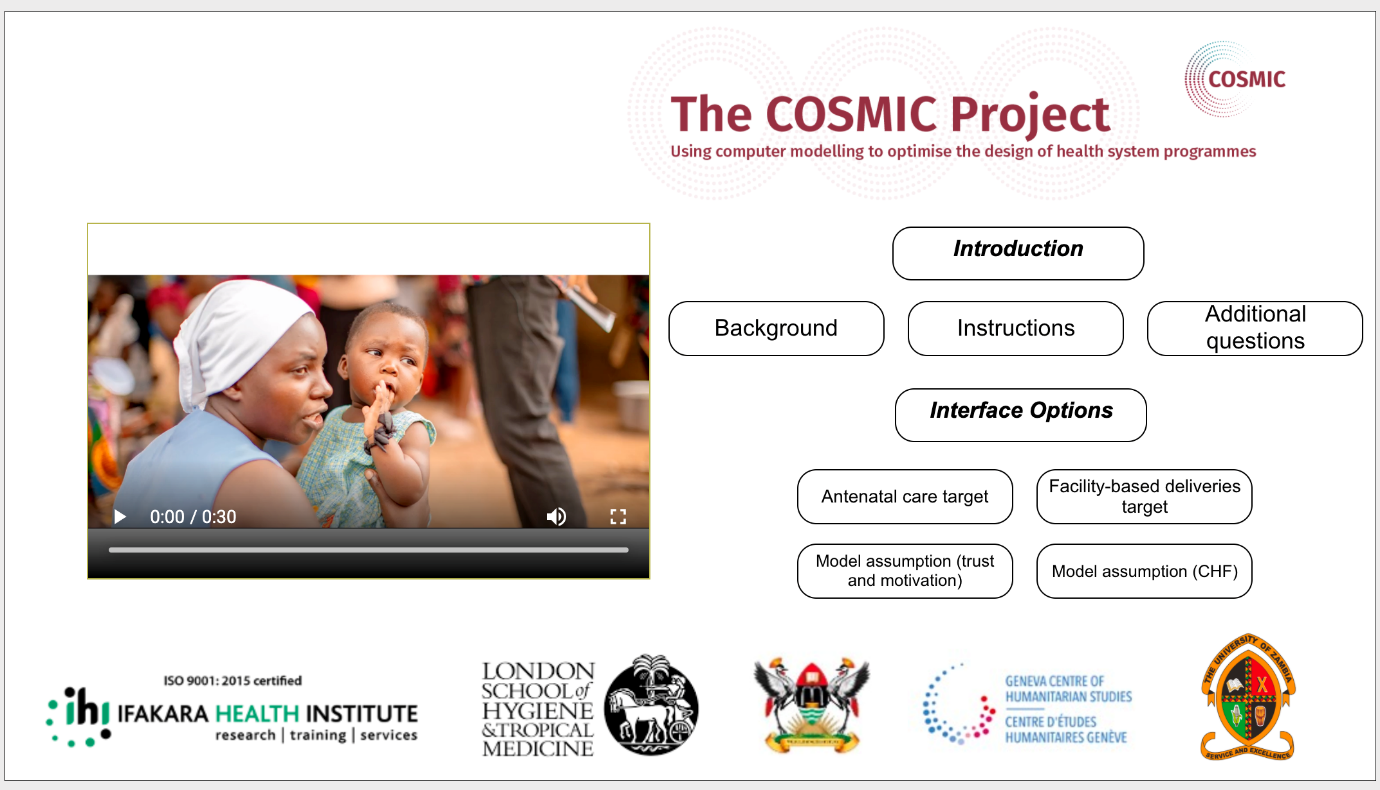


Figure S6.1: Slide 1 – Introduction slide.


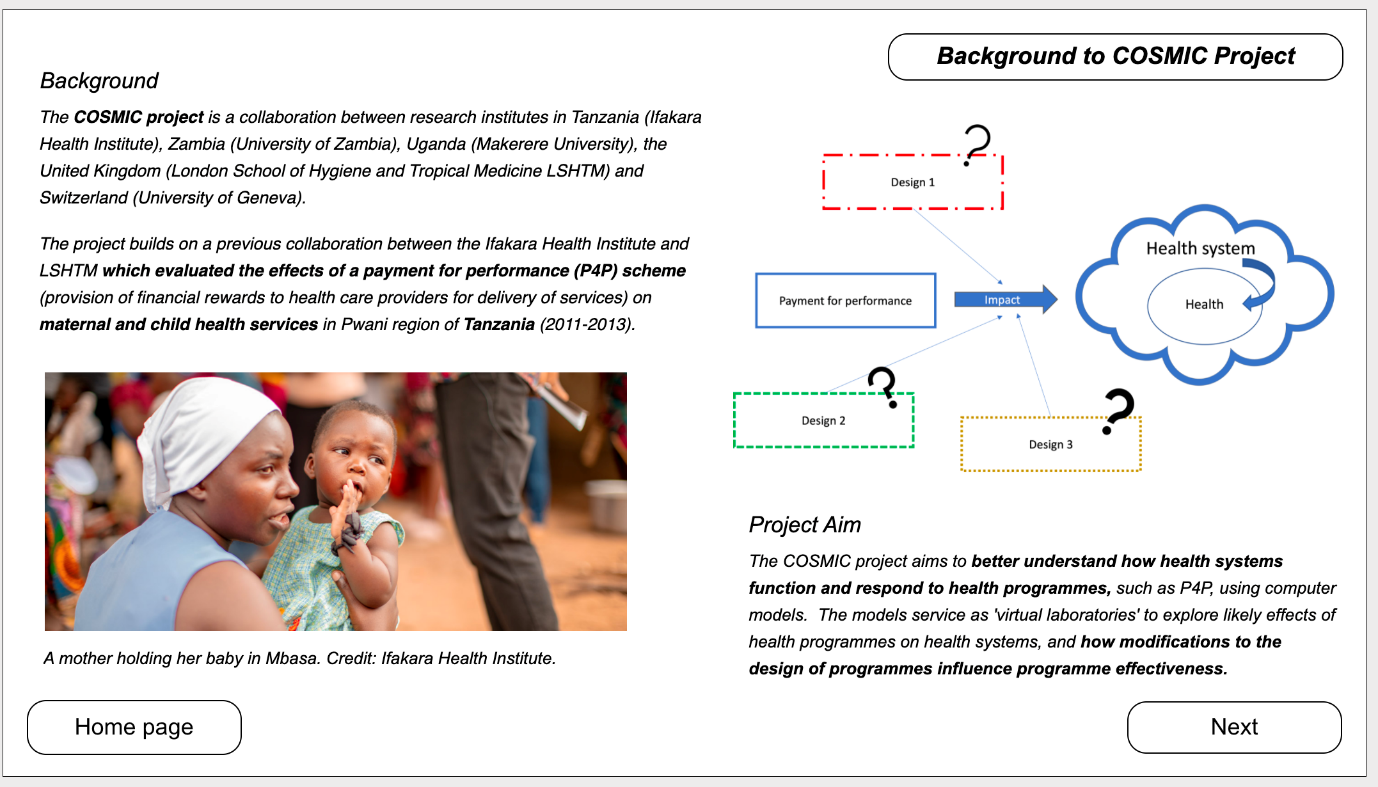


Figure S6.2: Slide 2 – Background (1) slide.


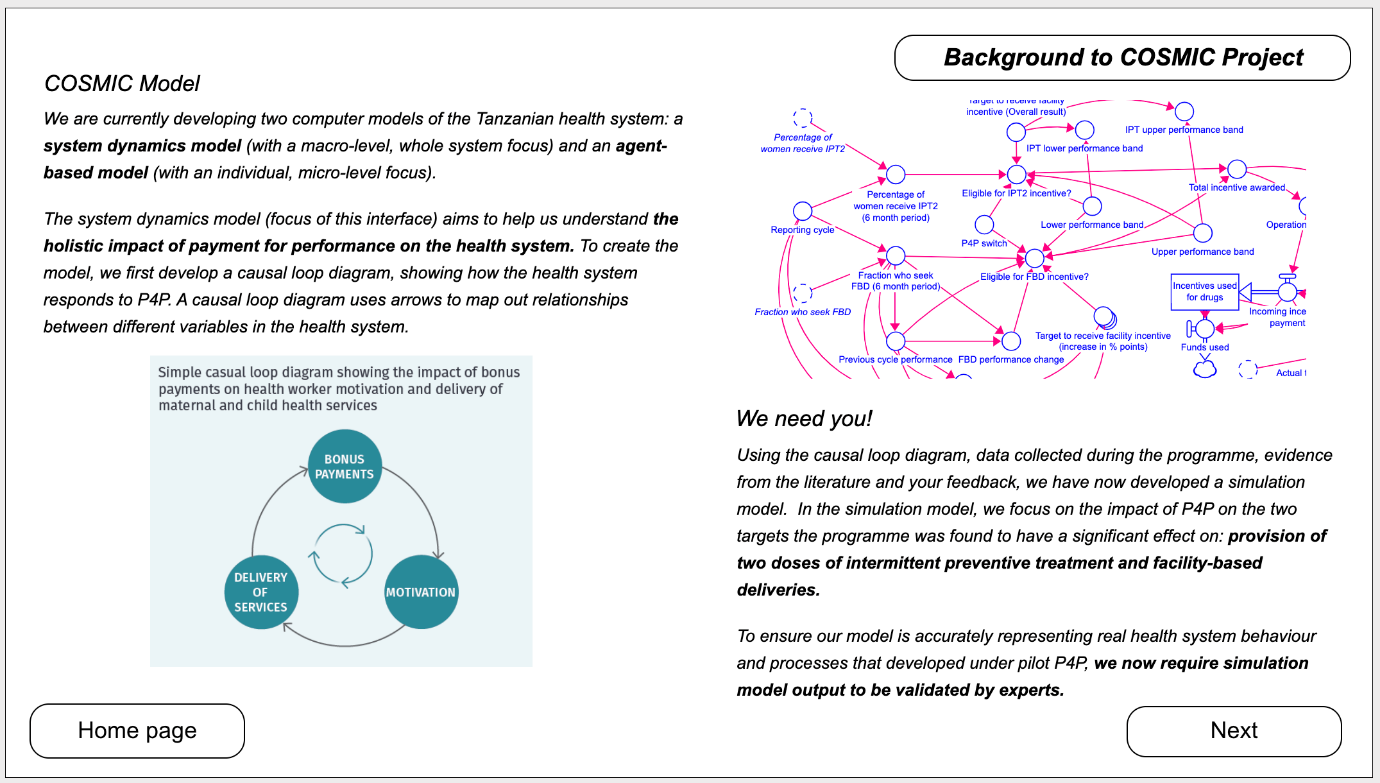


Figure S6.3: Slide 3 – Background (2) slide.


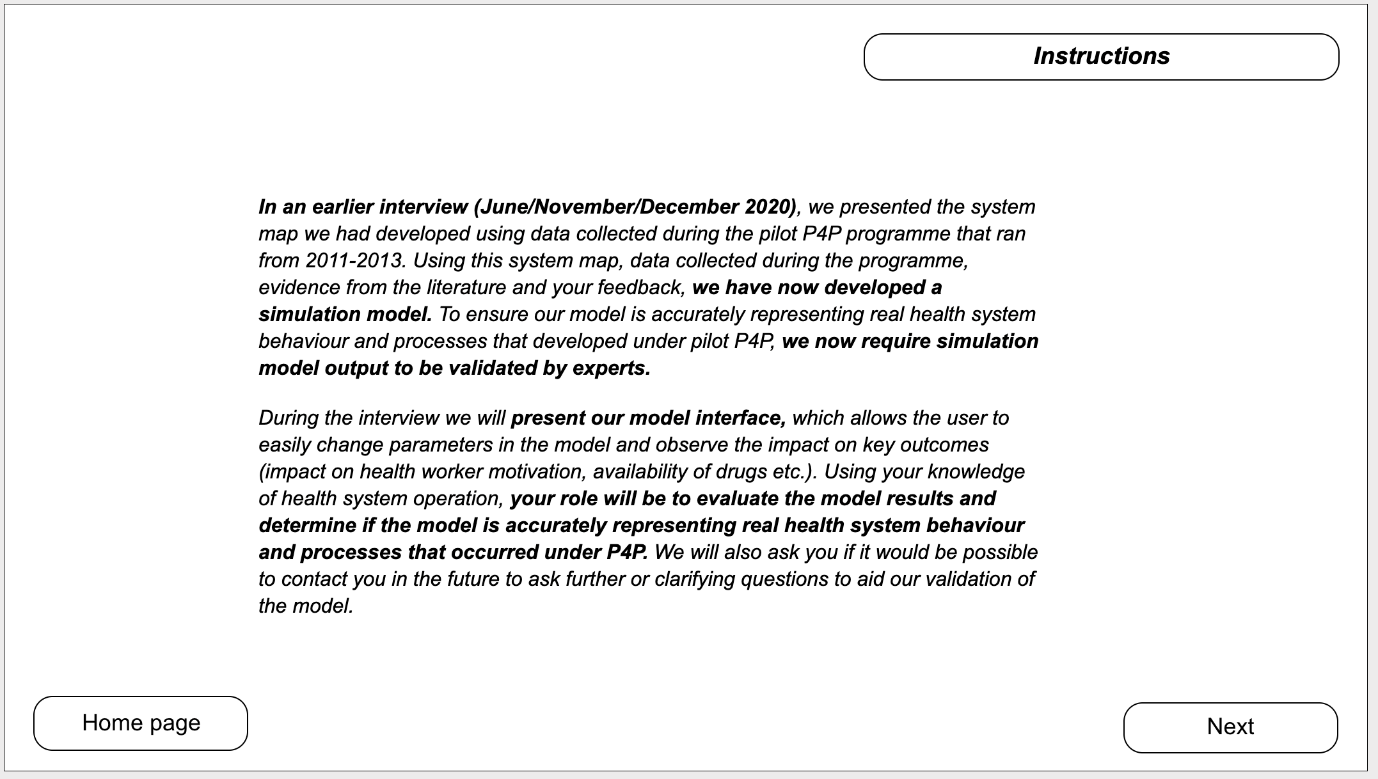


Figure S6.4: Slide 4 – Instruction slide.


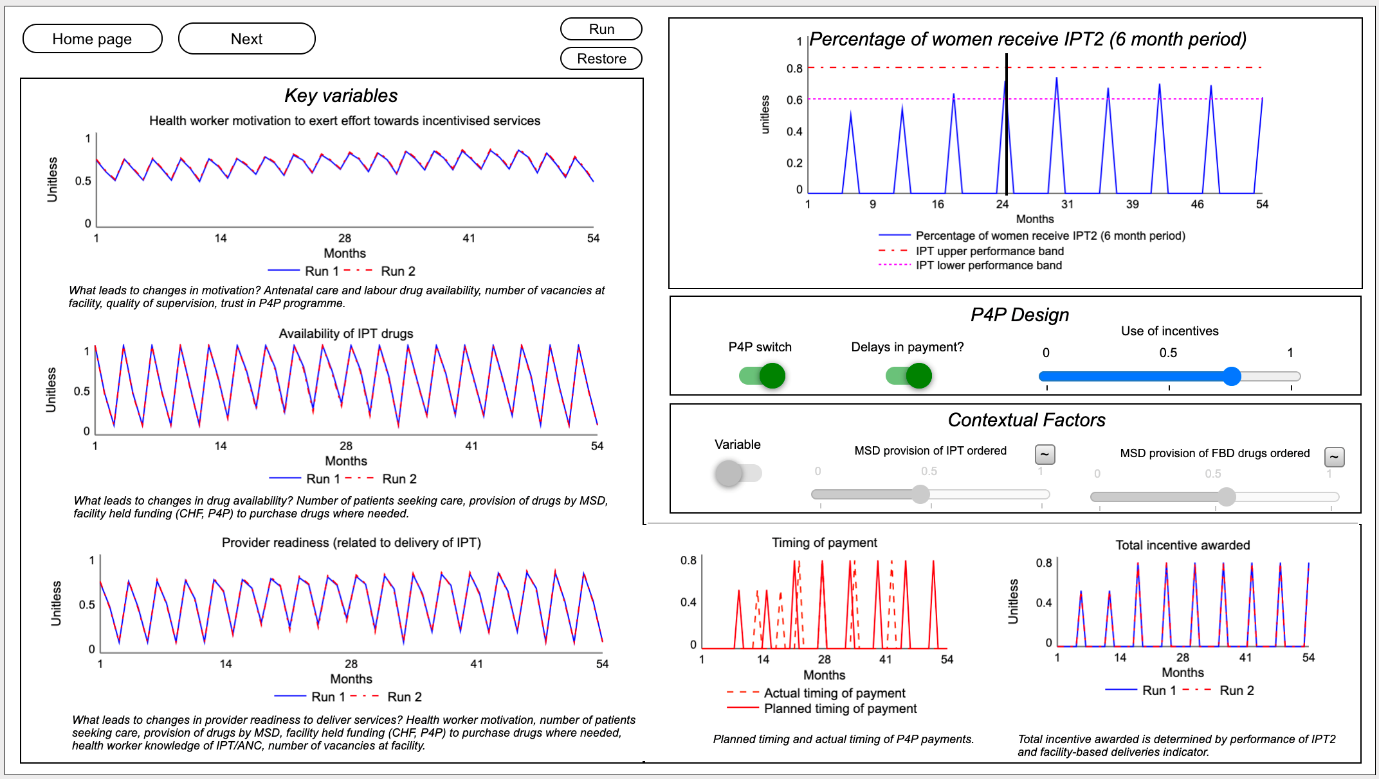


Figure S6.5: Slide 5 – Interactive slide to discuss dynamics around percentage of women who receive at least two doses of IPT during ANC.


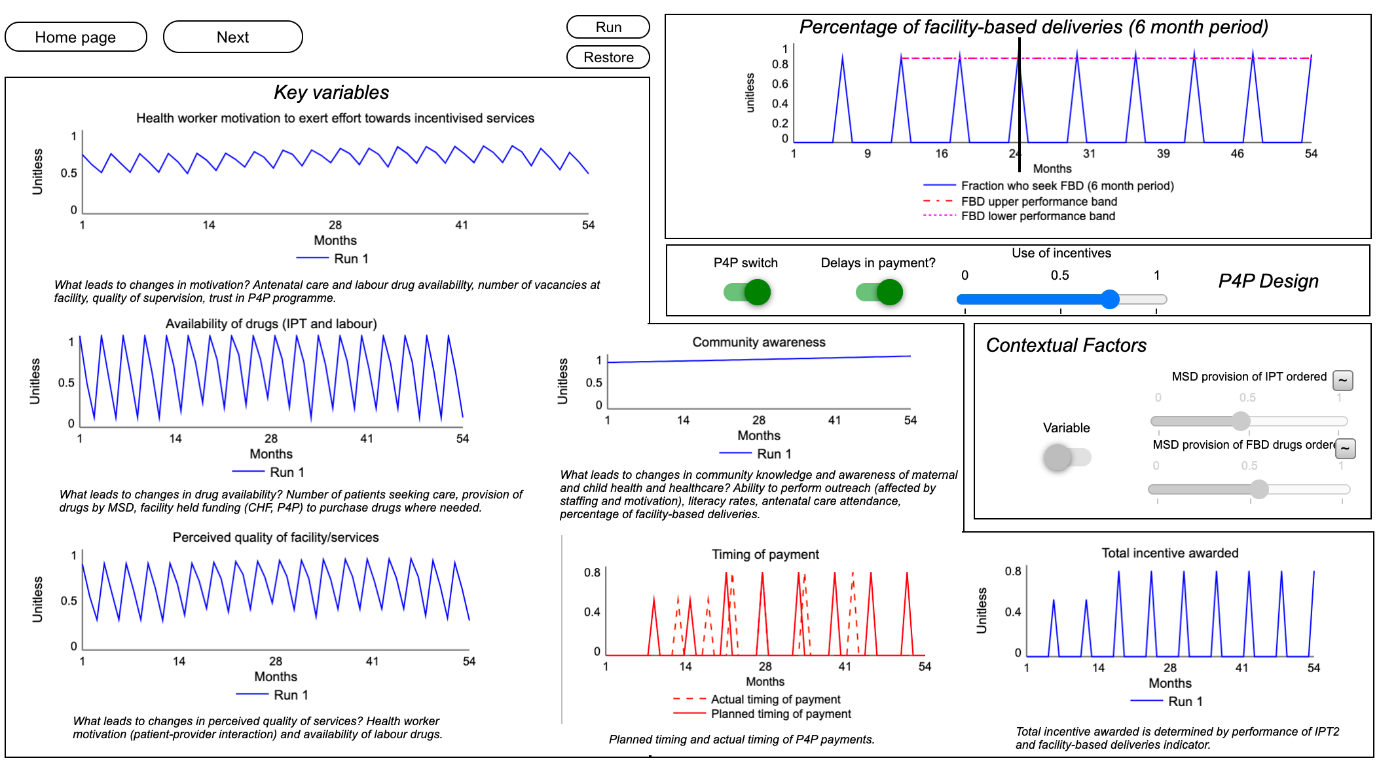


Figure S6.6: Slide 6 – Interactive slide to discuss dynamics around percentage of women who seek facility-based deliveries.


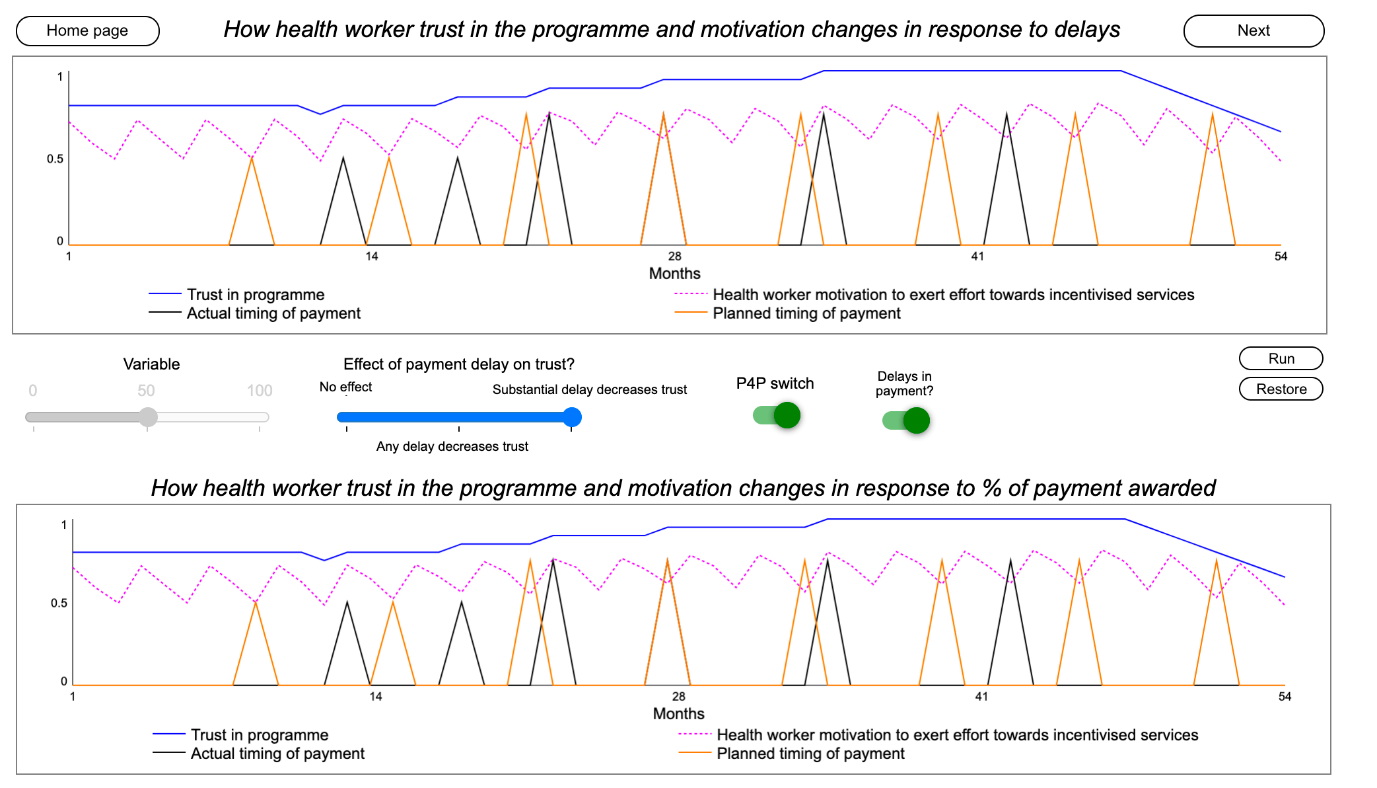


Figure S6.7: Slide 7 – Interactive slide to discuss how health worker trust and motivation responds to changes in timing of payments and amount of payment.


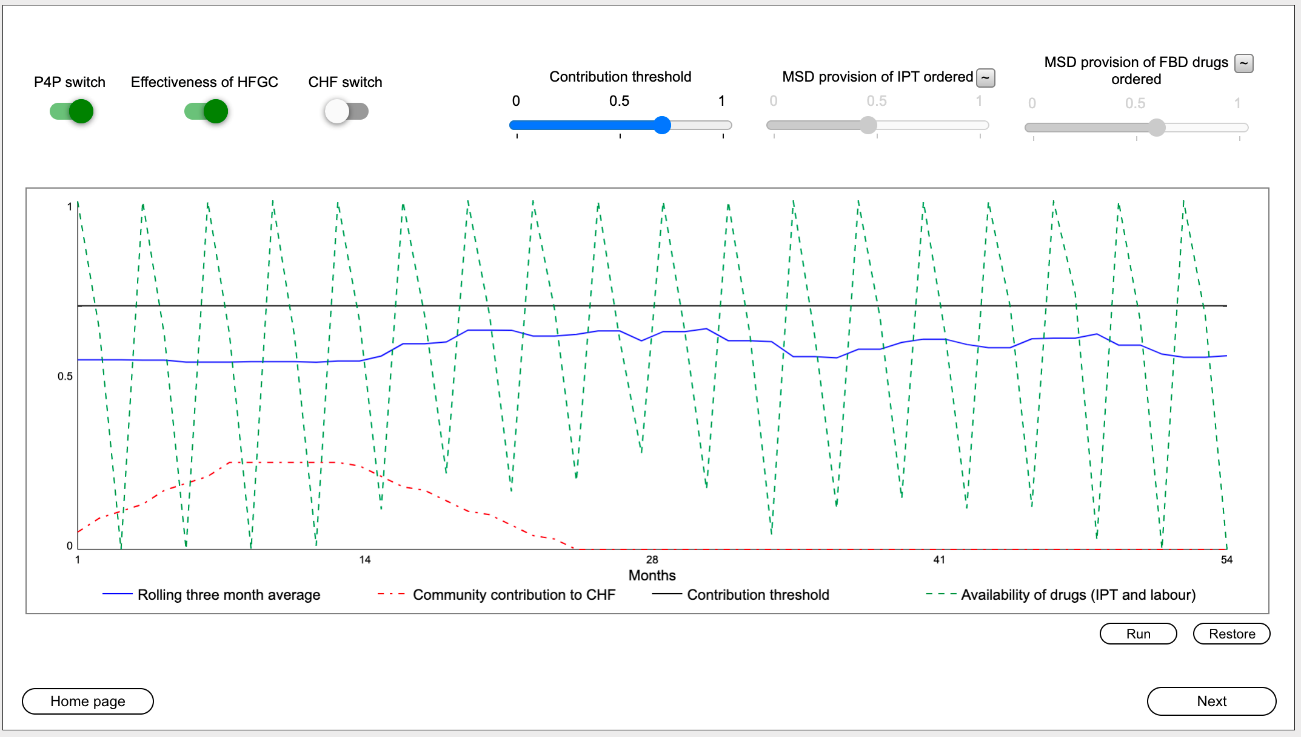


Figure S6.8: Slide 8 – Interactive slide to discuss how drug availability, perceived threshold of acceptable drug availability and effectiveness of Health Facility Governing Committee (HFGC) affect community payment into the Community Health Fund (CHF).


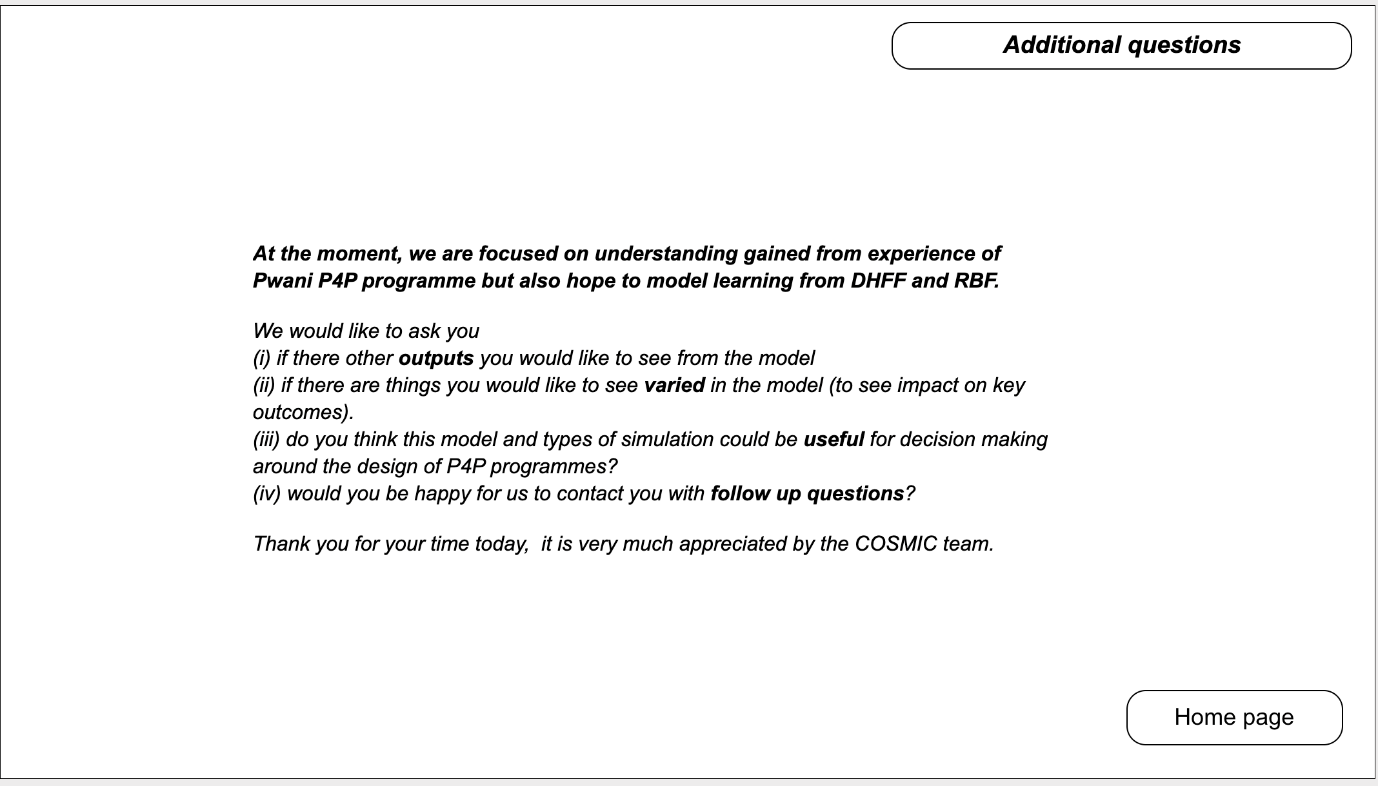


Figure S6.9: Slide 9 – Additional questions and conclusion slide.

*Supplementary File 7 – Changes to model post stakeholder consultation*

The feedback received during stakeholder consultations resulted in some new additions and adjustments to existing model structure, described here.

Key changes consisted of: (i) inclusion of an ‘alternative facility held funding’ variable (ii) adjustment of the effect of incentives on trust in programme and health worker motivation, and (iii) adjustment of effect of payment delays on trust in programme and health worker motivation. For change (i), stakeholders suggested inclusion of other types of facility held funding (other than P4P), as this would affect the purchasing power of facilities for buying additional drugs during the programme. In the current version of the model, this variable ‘alternative facility held funding available’ is static, but in future iterations of the model this will be dynamic. For change (ii), stakeholders commented that health workers would want to improve their performance, so a lower incentive payment (reflective of performance) would not be demotivating but would spur health workers on to try and improve their performance.

For change (iii), stakeholders were presented with three scenarios related to effect of payment delays in the model: i) payment delays do not affect trust and motivation, ii) any delay affects trust and motivation and iii) only severe delays (4+ months) affect trust and motivation. The consensus from stakeholders was that the third scenario, severe delays affect trust and motivation, was most likely, with communication of delays and expected payment dates sustaining trust and motivation up to a point. If payments are not made after a certain period (assumed 4+ months in the model), trust and motivation decrease until a payment is made. This relationship between payment delays, trust and motivation is retained in the model when payment delays are enacted.

Stakeholders remarked on the importance of community health workers and traditional birth attendants in increasing community awareness of services and escorting women to facilities for facility-based deliveries. These dynamics are not included in this current version of the model for the reasons set out in the paper (see sector selection).

*Supplementary File 8 – Sensitivity analysis*

The model was subjected to sensitivity analysis to determine the sensitivity of key outcomes (percentage of women who receive at least two doses of IPT during ANC, percentage of women who seek facility-based delivery) to changes in model parameters. Model parameters deemed appropriate for analysis were adjusted by 10%, with key outcome results recorded. Initial stock values, constant variable values (including table and graphical function values) were adjusted and simulated. Equation based parameters (flows and auxiliary variables) and constant variables where the value was not appropriate for adjustment (such as the ‘on’ and ‘off’ switch for turning the intervention on and off in the model, represented by ‘0’ and ‘1’ in the model) were not subjected to sensitivity analysis.

The following scale was used to determine sensitivity to changes in model variables; sensitive (5% ≤ change in outcome < 15%), very sensitive (15% ≤ change in outcome < 25%) and highly sensitive (25% ≥ change in outcome). Table cells are highlighted where outputs are categorised as sensitive (yellow), very sensitive (orange) and highly sensitive (red). The scale is adapted from Semwanga *et al.* (2016) and presented with smaller intervals for higher sensitivity categories, to further distinguish ‘very sensitive’ from ‘highly sensitive’ results. This analysis shed light on the likely effect of changes to programme design as well as contextual factors on key outcomes.

*Population sector*

Table S8.1: Sensitivity analyses for population sector parameters, change in key outcomes.

|  |  | **Percentage of women who receive at least two doses of IPT during ANC** | | | **Percentage of women who seek facility-based delivery** | | |
| --- | --- | --- | --- | --- | --- | --- | --- |
| **Parameter values adjusted** | **Adjustment** | *Baseline* | *Endline* | *Longterm* | *Baseline* | *Endline* | *Longterm* |
| Above50MortalityRate | -10% | 0.496 | 0.731 | 0.700 | 0.862 | 0.889 | 0.899 |
|  | 10% | 0.496 | 0.731 | 0.700 | 0.862 | 0.889 | 0.899 |
| AdultsAbove50(t) | -10% | 0.496 | 0.731 | 0.700 | 0.862 | 0.889 | 0.899 |
|  | 10% | 0.496 | 0.731 | 0.700 | 0.862 | 0.889 | 0.899 |
| ChildMortalityRate | -10% | 0.496 | 0.731 | 0.700 | 0.862 | 0.889 | 0.899 |
|  | 10% | 0.496 | 0.731 | 0.700 | 0.862 | 0.889 | 0.899 |
| Children(t) | -10% | 0.497 | 0.731 | 0.700 | 0.862 | 0.889 | 0.899 |
|  | 10% | 0.495 | 0.730 | 0.699 | 0.862 | 0.889 | 0.899 |
| Fraction_of_reproductive_age_adults_female | -10% | 0.519 | 0.733 | 0.700 | 0.863 | 0.889 | 0.899 |
|  | 10% | 0.477 | 0.729 | 0.700 | 0.862 | 0.889 | 0.899 |
| General_fertility_rate | -10% | 0.519 | 0.733 | 0.700 | 0.863 | 0.889 | 0.899 |
|  | 10% | 0.477 | 0.729 | 0.700 | 0.862 | 0.889 | 0.899 |
| InfantMortalityRate | -10% | 0.496 | 0.731 | 0.700 | 0.862 | 0.889 | 0.899 |
|  | 10% | 0.496 | 0.731 | 0.700 | 0.862 | 0.889 | 0.899 |
| Infants(t) | -10% | 0.496 | 0.731 | 0.700 | 0.862 | 0.889 | 0.899 |
|  | 10% | 0.496 | 0.731 | 0.700 | 0.862 | 0.889 | 0.899 |
| NeonateMortalityRate | -10% | 0.496 | 0.731 | 0.700 | 0.862 | 0.889 | 0.899 |
|  | 10% | 0.496 | 0.731 | 0.700 | 0.862 | 0.889 | 0.899 |
| Neonates(t) | -10% | 0.496 | 0.731 | 0.700 | 0.862 | 0.889 | 0.899 |
|  | 10% | 0.496 | 0.731 | 0.700 | 0.862 | 0.889 | 0.899 |
| PreschoolerMortalityRate | -10% | 0.496 | 0.731 | 0.700 | 0.862 | 0.889 | 0.899 |
|  | 10% | 0.496 | 0.731 | 0.700 | 0.862 | 0.889 | 0.899 |
| Preschoolers(t) | -10% | 0.496 | 0.731 | 0.700 | 0.862 | 0.889 | 0.899 |
|  | 10% | 0.496 | 0.731 | 0.700 | 0.862 | 0.889 | 0.899 |
| ReprodAgeMortalityRate | -10% | 0.496 | 0.731 | 0.700 | 0.862 | 0.889 | 0.899 |
|  | 10% | 0.496 | 0.731 | 0.700 | 0.862 | 0.889 | 0.899 |
| ReproductiveAgeAdults(t) | -10% | 0.518 | 0.732 | 0.699 | 0.862 | 0.889 | 0.899 |
|  | 10% | 0.478 | 0.730 | 0.700 | 0.862 | 0.889 | 0.899 |

Table S8.2: Sensitivity analyses for population sector parameters, change in key outcomes (% change).

|  |  | Percentage of women who receive at least two doses of IPT during ANC | | | Percentage of women who seek facility-based delivery | | |
| --- | --- | --- | --- | --- | --- | --- | --- |
| **Parameter values adjusted** | **Adjustment** | *Baseline* | *Endline* | *Longterm* | *Baseline* | *Endline* | *Longterm* |
| Above50MortalityRate | -10% | 0.0 | 0.0 | 0.0 | 0.0 | 0.0 | 0.0 |
|  | 10% | 0.0 | 0.0 | 0.0 | 0.0 | 0.0 | 0.0 |
| AdultsAbove50(t) | -10% | 0.0 | 0.0 | 0.0 | 0.0 | 0.0 | 0.0 |
|  | 10% | 0.0 | 0.0 | 0.0 | 0.0 | 0.0 | 0.0 |
| ChildMortalityRate | -10% | 0.0 | 0.0 | 0.0 | 0.0 | 0.0 | 0.0 |
|  | 10% | 0.0 | 0.0 | 0.0 | 0.0 | 0.0 | 0.0 |
| Children(t) | -10% | 0.1 | 0.1 | 0.1 | 0.0 | 0.0 | 0.0 |
|  | 10% | -0.1 | -0.1 | -0.1 | 0.0 | 0.0 | 0.0 |
| Fraction_of_reproductive_age_adults_female | -10% | 4.6 | 0.3 | 0.0 | 0.0 | 0.0 | 0.0 |
|  | 10% | -3.8 | -0.2 | 0.0 | 0.0 | 0.0 | 0.0 |
| General_fertility_rate | -10% | 4.6 | 0.3 | 0.0 | 0.0 | 0.0 | 0.0 |
|  | 10% | -3.8 | -0.2 | 0.0 | 0.0 | 0.0 | 0.0 |
| InfantMortalityRate | -10% | 0.0 | 0.0 | 0.0 | 0.0 | 0.0 | 0.0 |
|  | 10% | 0.0 | 0.0 | 0.0 | 0.0 | 0.0 | 0.0 |
| Infants(t) | -10% | 0.0 | 0.0 | 0.0 | 0.0 | 0.0 | 0.0 |
|  | 10% | 0.0 | 0.0 | 0.0 | 0.0 | 0.0 | 0.0 |
| NeonateMortalityRate | -10% | 0.0 | 0.0 | 0.0 | 0.0 | 0.0 | 0.0 |
|  | 10% | 0.0 | 0.0 | 0.0 | 0.0 | 0.0 | 0.0 |
| Neonates(t) | -10% | 0.0 | 0.0 | 0.0 | 0.0 | 0.0 | 0.0 |
|  | 10% | 0.0 | 0.0 | 0.0 | 0.0 | 0.0 | 0.0 |
| PreschoolerMortalityRate | -10% | 0.0 | 0.0 | 0.0 | 0.0 | 0.0 | 0.0 |
|  | 10% | 0.0 | 0.0 | 0.0 | 0.0 | 0.0 | 0.0 |
| Preschoolers(t) | -10% | 0.0 | 0.0 | 0.0 | 0.0 | 0.0 | 0.0 |
|  | 10% | 0.0 | 0.0 | 0.0 | 0.0 | 0.0 | 0.0 |
| ReprodAgeMortalityRate | -10% | 0.0 | 0.0 | 0.0 | 0.0 | 0.0 | 0.0 |
|  | 10% | 0.0 | 0.0 | 0.0 | 0.0 | 0.0 | 0.0 |
| ReproductiveAgeAdults(t) | -10% | 4.4 | 0.2 | -0.1 | 0.0 | 0.0 | 0.0 |
|  | 10% | -3.6 | -0.1 | 0.1 | 0.0 | 0.0 | 0.0 |

*Demand and Services sector*

Table S8.3: Sensitivity analyses for demand and services (antenatal care) sector parameters, change in key outcomes.

|  |  | Percentage of women who receive at least two doses of IPT during ANC | | | Percentage of women who seek facility-based delivery | | |
| --- | --- | --- | --- | --- | --- | --- | --- |
| **Parameter values adjusted** | **Adjustment** | *Baseline* | *Endline* | *Longterm* | *Baseline* | *Endline* | *Longterm* |
| ANC_visit_1(t) | -10% | 0.487 | 0.731 | 0.700 | 0.862 | 0.889 | 0.899 |
|  | 10% | 0.505 | 0.731 | 0.700 | 0.863 | 0.889 | 0.899 |
| ANC_visit_2(t) | -10% | 0.496 | 0.731 | 0.700 | 0.862 | 0.889 | 0.899 |
|  | 10% | 0.496 | 0.731 | 0.700 | 0.862 | 0.889 | 0.899 |
| ANC_visit_2a(t) | -10% | 0.489 | 0.730 | 0.700 | 0.862 | 0.889 | 0.899 |
|  | 10% | 0.503 | 0.731 | 0.700 | 0.863 | 0.889 | 0.899 |
| ANC_visit_3(t) | -10% | 0.496 | 0.731 | 0.700 | 0.862 | 0.889 | 0.899 |
|  | 10% | 0.496 | 0.731 | 0.700 | 0.862 | 0.889 | 0.899 |
| ANC_visit_3a(t) | -10% | 0.493 | 0.731 | 0.700 | 0.862 | 0.889 | 0.899 |
|  | 10% | 0.499 | 0.731 | 0.700 | 0.862 | 0.889 | 0.899 |
| ANC_visit_3b(t) | -10% | 0.495 | 0.731 | 0.700 | 0.862 | 0.889 | 0.899 |
|  | 10% | 0.497 | 0.731 | 0.700 | 0.862 | 0.889 | 0.899 |
| ANC_visit_4(t) | -10% | 0.496 | 0.731 | 0.700 | 0.862 | 0.889 | 0.899 |
|  | 10% | 0.496 | 0.731 | 0.700 | 0.862 | 0.889 | 0.899 |
| ANC_visit_4a(t) | -10% | 0.496 | 0.731 | 0.700 | 0.862 | 0.889 | 0.899 |
|  | 10% | 0.496 | 0.731 | 0.700 | 0.862 | 0.889 | 0.899 |
| ANC_visit_4b(t) | -10% | 0.495 | 0.731 | 0.700 | 0.862 | 0.889 | 0.899 |
|  | 10% | 0.497 | 0.731 | 0.700 | 0.862 | 0.889 | 0.899 |
| ANC_visit_4c(t) | -10% | 0.496 | 0.731 | 0.700 | 0.862 | 0.889 | 0.899 |
|  | 10% | 0.496 | 0.731 | 0.700 | 0.862 | 0.889 | 0.899 |
| Dropoutrate1 | -10% | 0.496 | 0.731 | 0.700 | 0.863 | 0.890 | 0.899 |
|  | 10% | 0.496 | 0.731 | 0.700 | 0.862 | 0.889 | 0.898 |
| Dropoutrate2 | -10% | 0.497 | 0.733 | 0.702 | 0.863 | 0.889 | 0.899 |
|  | 10% | 0.495 | 0.729 | 0.698 | 0.862 | 0.889 | 0.899 |
| Dropoutrate3 | -10% | 0.499 | 0.734 | 0.703 | 0.863 | 0.890 | 0.899 |
|  | 10% | 0.493 | 0.728 | 0.697 | 0.862 | 0.889 | 0.898 |
| Dropoutrate4 | -10% | 0.501 | 0.735 | 0.705 | 0.863 | 0.890 | 0.899 |
|  | 10% | 0.491 | 0.726 | 0.695 | 0.862 | 0.889 | 0.898 |

Table S8.4: Sensitivity analyses for demand and services (antenatal care) sector parameters, change in key outcomes (% change).

|  |  | Percentage of women who receive at least two doses of IPT during ANC | | | Percentage of women who seek facility-based delivery | | |
| --- | --- | --- | --- | --- | --- | --- | --- |
| **Parameter values adjusted** | **Adjustment** | *Baseline* | *Endline* | *Longterm* | *Baseline* | *Endline* | *Longterm* |
| ANC_visit_1(t) | -10% | -1.7 | 0.0 | 0.0 | 0.0 | 0.0 | 0.0 |
|  | 10% | 1.7 | 0.0 | 0.0 | 0.0 | 0.0 | 0.0 |
| ANC_visit_2(t) | -10% | -0.1 | 0.0 | 0.0 | 0.0 | 0.0 | 0.0 |
|  | 10% | 0.1 | 0.0 | 0.0 | 0.0 | 0.0 | 0.0 |
| ANC_visit_2a(t) | -10% | -1.4 | 0.0 | 0.0 | 0.0 | 0.0 | 0.0 |
|  | 10% | 1.4 | 0.0 | 0.0 | 0.0 | 0.0 | 0.0 |
| ANC_visit_3(t) | -10% | 0.0 | 0.0 | 0.0 | 0.0 | 0.0 | 0.0 |
|  | 10% | 0.0 | 0.0 | 0.0 | 0.0 | 0.0 | 0.0 |
| ANC_visit_3a(t) | -10% | -0.5 | 0.0 | 0.0 | 0.0 | 0.0 | 0.0 |
|  | 10% | 0.5 | 0.0 | 0.0 | 0.0 | 0.0 | 0.0 |
| ANC_visit_3b(t) | -10% | -0.2 | 0.0 | 0.0 | 0.0 | 0.0 | 0.0 |
|  | 10% | 0.2 | 0.0 | 0.0 | 0.0 | 0.0 | 0.0 |
| ANC_visit_4(t) | -10% | 0.0 | 0.0 | 0.0 | 0.0 | 0.0 | 0.0 |
|  | 10% | 0.0 | 0.0 | 0.0 | 0.0 | 0.0 | 0.0 |
| ANC_visit_4a(t) | -10% | -0.1 | 0.0 | 0.0 | 0.0 | 0.0 | 0.0 |
|  | 10% | 0.1 | 0.0 | 0.0 | 0.0 | 0.0 | 0.0 |
| ANC_visit_4b(t) | -10% | -0.2 | 0.0 | 0.0 | 0.0 | 0.0 | 0.0 |
|  | 10% | 0.2 | 0.0 | 0.0 | 0.0 | 0.0 | 0.0 |
| ANC_visit_4c(t) | -10% | 0.0 | 0.0 | 0.0 | 0.0 | 0.0 | 0.0 |
|  | 10% | 0.0 | 0.0 | 0.0 | 0.0 | 0.0 | 0.0 |
| Dropoutrate1 | -10% | 0.0 | 0.0 | 0.0 | 0.1 | 0.0 | 0.0 |
|  | 10% | 0.0 | 0.0 | 0.0 | -0.1 | 0.0 | 0.0 |
| Dropoutrate2 | -10% | 0.2 | 0.3 | 0.3 | 0.0 | 0.0 | 0.0 |
|  | 10% | -0.2 | -0.3 | -0.3 | 0.0 | 0.0 | 0.0 |
| Dropoutrate3 | -10% | 0.5 | 0.4 | 0.4 | 0.0 | 0.0 | 0.0 |
|  | 10% | -0.5 | -0.4 | -0.4 | 0.0 | 0.0 | 0.0 |
| Dropoutrate4 | -10% | 1.0 | 0.6 | 0.7 | 0.1 | 0.1 | 0.1 |
|  | 10% | -1.0 | -0.6 | -0.7 | -0.1 | -0.1 | -0.1 |

Table S8.5: Sensitivity analyses for demand and services (facility-based delivery care) sector parameters, change in key outcomes.

|  |  | **Percentage of women who receive at least two doses of IPT during ANC** | | | **Percentage of women who seek facility-based delivery** | | | |
| --- | --- | --- | --- | --- | --- | --- | --- | --- |
| **Parameter values adjusted** | **Adjustment** | *Baseline* | *Endline* | *Longterm* | | *Baseline* | *Endline* | *Longterm* |
| Belief_in_myths | -10% | 0.496 | 0.731 | 0.700 | | 0.862 | 0.889 | 0.899 |
|  | 10% | 0.496 | 0.731 | 0.700 | | 0.862 | 0.889 | 0.899 |
| Community_awareness(t) | -10% | 0.496 | 0.637 | 0.702 | | 0.839 | 0.857 | 0.871 |
|  | 10% | 0.496 | 0.731 | 0.700 | | 0.886 | 0.914 | 0.914 |
| Decay_awareness | -10% | 0.496 | 0.731 | 0.700 | | 0.862 | 0.889 | 0.899 |
|  | 10% | 0.496 | 0.731 | 0.700 | | 0.862 | 0.889 | 0.899 |
| Distance_to_facility | -10% | 0.496 | 0.637 | 0.702 | | 0.836 | 0.854 | 0.871 |
|  | 10% | 0.496 | 0.731 | 0.700 | | 0.873 | 0.895 | 0.909 |
| Effect_ANC_awareness | -10% | 0.496 | 0.731 | 0.700 | | 0.862 | 0.889 | 0.898 |
|  | 10% | 0.496 | 0.731 | 0.700 | | 0.863 | 0.890 | 0.900 |
| Effect_FBD_awareness | -10% | 0.496 | 0.731 | 0.700 | | 0.862 | 0.889 | 0.898 |
|  | 10% | 0.496 | 0.731 | 0.700 | | 0.863 | 0.890 | 0.900 |
| Effect_literacy_awareness | -10% | 0.496 | 0.731 | 0.700 | | 0.862 | 0.889 | 0.899 |
|  | 10% | 0.496 | 0.731 | 0.700 | | 0.862 | 0.889 | 0.899 |
| Effect_myths | -10% | 0.496 | 0.731 | 0.700 | | 0.862 | 0.889 | 0.899 |
|  | 10% | 0.496 | 0.731 | 0.700 | | 0.862 | 0.889 | 0.899 |
| Effect_outreach_awareness | -10% | 0.496 | 0.731 | 0.700 | | 0.862 | 0.889 | 0.898 |
|  | 10% | 0.496 | 0.731 | 0.700 | | 0.862 | 0.889 | 0.899 |
| Literacy | -10% | 0.496 | 0.731 | 0.700 | | 0.862 | 0.889 | 0.899 |
|  | 10% | 0.496 | 0.731 | 0.700 | | 0.862 | 0.889 | 0.899 |
| "Number_of_facility-based_deliveries"(t) | -10% | 0.496 | 0.731 | 0.700 | | 0.862 | 0.889 | 0.899 |
|  | 10% | 0.496 | 0.731 | 0.700 | | 0.862 | 0.889 | 0.899 |
| Prob_FBD_ANC_1 | -10% | 0.496 | 0.731 | 0.700 | | 0.862 | 0.889 | 0.898 |
|  | 10% | 0.496 | 0.731 | 0.700 | | 0.863 | 0.890 | 0.899 |
| Prob_FBD_ANC_2 | -10% | 0.496 | 0.731 | 0.700 | | 0.861 | 0.888 | 0.897 |
|  | 10% | 0.496 | 0.731 | 0.700 | | 0.864 | 0.891 | 0.900 |
| Prob_FBD_ANC_3 | -10% | 0.496 | 0.698 | 0.699 | | 0.856 | 0.881 | 0.892 |
|  | 10% | 0.496 | 0.731 | 0.700 | | 0.869 | 0.896 | 0.906 |
| Prob_FBD_ANC_4 | -10% | 0.496 | 0.698 | 0.699 | | 0.845 | 0.870 | 0.882 |
|  | 10% | 0.496 | 0.731 | 0.700 | | 0.880 | 0.906 | 0.915 |

Table S8.6: Sensitivity analyses for (facility-based delivery care) sector parameters, change in key outcomes (% change).

|  |  | **Percentage of women who receive at least two doses of IPT during ANC** | | | **Percentage of women who seek facility-based delivery** | | | |
| --- | --- | --- | --- | --- | --- | --- | --- | --- |
| **Parameter values adjusted** | **Adjustment** | *Baseline* | *Endline* | *Longterm* | | *Baseline* | *Endline* | *Longterm* |
| Belief_in_myths | -10% | 0.0 | 0.0 | 0.0 | | 0.0 | 0.0 | 0.0 |
|  | 10% | 0.0 | 0.0 | 0.0 | | 0.0 | 0.0 | 0.0 |
| Community_awareness(t) | -10% | 0.0 | -12.8 | 0.2 | | -2.8 | -3.6 | -3.0 |
|  | 10% | 0.0 | 0.0 | 0.0 | | 2.8 | 2.8 | 1.6 |
| Decay_awareness | -10% | 0.0 | 0.0 | 0.0 | | 0.0 | 0.0 | 0.0 |
|  | 10% | 0.0 | 0.0 | 0.0 | | 0.0 | 0.0 | 0.0 |
| Distance_to_facility | -10% | 0.0 | -12.8 | 0.2 | | -3.1 | -3.9 | -3.1 |
|  | 10% | 0.0 | 0.0 | 0.0 | | 1.2 | 0.7 | 1.1 |
| Effect_ANC_awareness | -10% | 0.0 | 0.0 | 0.0 | | 0.0 | -0.1 | -0.1 |
|  | 10% | 0.0 | 0.0 | 0.0 | | 0.0 | 0.1 | 0.1 |
| Effect_FBD_awareness | -10% | 0.0 | 0.0 | 0.0 | | 0.0 | 0.0 | -0.1 |
|  | 10% | 0.0 | 0.0 | 0.0 | | 0.0 | 0.0 | 0.1 |
| Effect_literacy_awareness | -10% | 0.0 | 0.0 | 0.0 | | 0.0 | 0.0 | 0.0 |
|  | 10% | 0.0 | 0.0 | 0.0 | | 0.0 | 0.0 | 0.0 |
| Effect_myths | -10% | 0.0 | 0.0 | 0.0 | | 0.0 | 0.0 | 0.0 |
|  | 10% | 0.0 | 0.0 | 0.0 | | 0.0 | 0.0 | 0.0 |
| Effect_outreach_awareness | -10% | 0.0 | 0.0 | 0.0 | | 0.0 | 0.0 | -0.1 |
|  | 10% | 0.0 | 0.0 | 0.0 | | 0.0 | 0.0 | 0.1 |
| Literacy | -10% | 0.0 | 0.0 | 0.0 | | 0.0 | 0.0 | 0.0 |
|  | 10% | 0.0 | 0.0 | 0.0 | | 0.0 | 0.0 | 0.0 |
| "Number_of_facility-based_deliveries"(t) | -10% | 0.0 | 0.0 | 0.0 | | 0.0 | 0.0 | 0.0 |
|  | 10% | 0.0 | 0.0 | 0.0 | | 0.0 | 0.0 | 0.0 |
| Prob_FBD_ANC_1 | -10% | 0.0 | 0.0 | 0.0 | | -0.1 | -0.1 | -0.1 |
|  | 10% | 0.0 | 0.0 | 0.0 | | 0.1 | 0.1 | 0.1 |
| Prob_FBD_ANC_2 | -10% | 0.0 | 0.0 | 0.0 | | -0.2 | -0.2 | -0.2 |
|  | 10% | 0.0 | 0.0 | 0.0 | | 0.2 | 0.2 | 0.2 |
| Prob_FBD_ANC_3 | -10% | 0.0 | -4.5 | -0.1 | | -0.7 | -0.9 | -0.8 |
|  | 10% | 0.0 | 0.0 | 0.0 | | 0.7 | 0.7 | 0.8 |
| Prob_FBD_ANC_4 | -10% | 0.0 | -4.5 | -0.1 | | -2.0 | -2.1 | -1.9 |
|  | 10% | 0.0 | 0.0 | 0.0 | | 2.0 | 1.9 | 1.9 |

*Facility Commodities sector*

Table S8.7: Sensitivity analyses for facility commodities (antenatal care) sector parameters, change in key outcomes.

|  |  | **Percentage of women who receive at least two doses of IPT during ANC** | | | **Percentage of women who seek facility-based delivery** | | |
| --- | --- | --- | --- | --- | --- | --- | --- |
| **Parameter values adjusted** | **Adjustment** | *Baseline* | *Endline* | *Longterm* | *Baseline* | *Endline* | *Longterm* |
| MSD_provision_of_IPT_ordered | -10% | 0.440 | 0.666 | 0.635 | 0.860 | 0.884 | 0.897 |
|  | 10% | 0.569 | 0.786 | 0.753 | 0.865 | 0.895 | 0.901 |
| Order_of_IPT_drugs(t) | -10% | 0.490 | 0.730 | 0.700 | 0.862 | 0.889 | 0.899 |
|  | 10% | 0.502 | 0.731 | 0.700 | 0.862 | 0.889 | 0.899 |
| Stock_of_IPT_drugs(t) | -10% | 0.496 | 0.731 | 0.700 | 0.862 | 0.889 | 0.899 |
|  | 10% | 0.489 | 0.731 | 0.700 | 0.862 | 0.889 | 0.899 |

Table S8.8: Sensitivity analyses for facility commodities (antenatal care) sector parameters, change in key outcomes (% change).

|  |  | **Percentage of women who receive at least two doses of IPT during ANC** | | | **Percentage of women who seek facility-based delivery** | | |
| --- | --- | --- | --- | --- | --- | --- | --- |
| **Parameter values adjusted** | **Adjustment** | *Baseline* | *Endline* | *Longterm* | *Baseline* | *Endline* | *Longterm* |
| MSD_provision_of_IPT_ordered | -10% | -11.3 | -8.8 | -9.3 | -0.3 | -0.6 | -0.2 |
|  | 10% | 14.7 | 7.5 | 7.6 | 0.3 | 0.6 | 0.2 |
| Order_of_IPT_drugs(t) | -10% | -1.2 | -0.1 | 0.0 | 0.0 | 0.0 | 0.0 |
|  | 10% | 1.2 | 0.1 | 0.0 | 0.0 | 0.0 | 0.0 |
| Stock_of_IPT_drugs(t) | -10% | -0.1 | 0.0 | 0.0 | 0.0 | 0.0 | 0.0 |
|  | 10% | -1.3 | 0.0 | 0.0 | 0.0 | 0.0 | 0.0 |

Table S8.9: Sensitivity analyses for facility commodities (facility-based delivery care) parameters, change in key outcomes.

|  |  | **Percentage of women who receive at least two doses of IPT during ANC** | | | **Percentage of women who seek facility-based delivery** | | |
| --- | --- | --- | --- | --- | --- | --- | --- |
| **Parameter values adjusted** | **Adjustment** | *Baseline* | *Endline* | *Longterm* | *Baseline* | *Endline* | *Longterm* |
| MSD_provision_of_FBD_drugs_ordered | -10% | 0.496 | 0.731 | 0.700 | 0.859 | 0.888 | 0.898 |
|  | 10% | 0.496 | 0.731 | 0.700 | 0.865 | 0.891 | 0.902 |
| Order_of_FBD_drugs(t) | -10% | 0.496 | 0.731 | 0.700 | 0.862 | 0.889 | 0.899 |
|  | 10% | 0.496 | 0.731 | 0.700 | 0.862 | 0.889 | 0.899 |
| Stock_of_FBD_drugs(t) | -10% | 0.496 | 0.731 | 0.700 | 0.862 | 0.889 | 0.899 |
|  | 10% | 0.496 | 0.731 | 0.700 | 0.862 | 0.889 | 0.899 |

Table S8.10: Sensitivity analyses for facility commodities (facility-based delivery care) parameters, change in key outcomes (% change).

|  |  | **Percentage of women who receive at least two doses of IPT during ANC** | | | **Percentage of women who seek facility-based delivery** | | |
| --- | --- | --- | --- | --- | --- | --- | --- |
| **Parameter values adjusted** | **Adjustment** | *Baseline* | *Endline* | *Longterm* | *Baseline* | *Endline* | *Longterm* |
| MSD_provision_of_FBD_drugs_ordered | -10% | 0.0 | 0.0 | 0.0 | -0.4 | -0.2 | -0.1 |
|  | 10% | 0.0 | 0.0 | 0.0 | 0.2 | 0.2 | 0.4 |
| Order_of_FBD_drugs(t) | -10% | 0.0 | 0.0 | 0.0 | 0.0 | 0.0 | 0.0 |
|  | 10% | 0.0 | 0.0 | 0.0 | 0.0 | 0.0 | 0.0 |
| Stock_of_FBD_drugs(t) | -10% | 0.0 | 0.0 | 0.0 | 0.0 | 0.0 | 0.0 |
|  | 10% | 0.0 | 0.0 | 0.0 | 0.0 | 0.0 | 0.0 |

*Facility Operations sector*

Table S8.11: Sensitivity analyses for facility operations sector parameters, change in key outcomes.

|  |  | **Percentage of women who receive at least two doses of IPT during ANC** | | | **Percentage of women who seek facility-based delivery** | | |
| --- | --- | --- | --- | --- | --- | --- | --- |
| **Parameter values adjusted** | **Adjustment** | *Baseline* | *Endline* | *Longterm* | *Baseline* | *Endline* | *Longterm* |
| Average_attrition_rate | -10% | 0.496 | 0.731 | 0.700 | 0.862 | 0.889 | 0.899 |
|  | 10% | 0.496 | 0.730 | 0.700 | 0.862 | 0.889 | 0.899 |
| Effect_of_delays_in_payment_on_trust [3] | -10% | 0.496 | 0.731 | 0.700 | 0.862 | 0.889 | 0.899 |
|  | 10% | 0.496 | 0.731 | 0.700 | 0.862 | 0.889 | 0.900 |
| "Effect_of_incentive_on_trust_(change)" | -10% | 0.496 | 0.731 | 0.700 | 0.862 | 0.889 | 0.899 |
|  | 10% | 0.496 | 0.731 | 0.700 | 0.862 | 0.889 | 0.899 |
| Effect_of_supervision_on_knowledge[1, 1] | -10% | 0.496 | 0.730 | 0.700 | 0.862 | 0.889 | 0.899 |
|  | 10% | 0.496 | 0.731 | 0.700 | 0.862 | 0.889 | 0.899 |
| Hiring_rate | -10% | 0.496 | 0.730 | 0.700 | 0.862 | 0.889 | 0.898 |
|  | 10% | 0.496 | 0.731 | 0.700 | 0.862 | 0.889 | 0.899 |
| "Knowledge_of_health_workers_(IPT)"(t) | -10% | 0.497 | 0.729 | 0.701 | 0.863 | 0.891 | 0.899 |
|  | 10% | 0.494 | 0.733 | 0.700 | 0.862 | 0.888 | 0.899 |
| "Number_of_health_worker_at_health_facility_(%_filled)"(t) | -10% | 0.497 | 0.729 | 0.700 | 0.862 | 0.889 | 0.898 |
|  | 10% | 0.495 | 0.733 | 0.699 | 0.863 | 0.889 | 0.900 |
| Trust_in_programme(t) | -10% | 0.496 | 0.730 | 0.700 | 0.861 | 0.888 | 0.898 |
|  | 10% | 0.496 | 0.731 | 0.700 | 0.864 | 0.891 | 0.900 |

Table S8.12: Sensitivity analyses for facility operations sector parameters, change in key outcomes (% change).

|  |  | **Percentage of women who receive at least two doses of IPT during ANC** | | | **Percentage of women who seek facility-based delivery** | | | |
| --- | --- | --- | --- | --- | --- | --- | --- | --- |
| **Parameter values adjusted** | **Adjustment** | *Baseline* | *Endline* | *Longterm* | | *Baseline* | *Endline* | *Longterm* |
| Average_attrition_rate | -10% | 0.0 | 0.0 | 0.1 | | 0.0 | 0.0 | 0.0 |
|  | 10% | 0.0 | 0.0 | 0.0 | | 0.0 | 0.0 | 0.0 |
| Effect_of_delays_in_payment_on_trust [3] | -10% | 0.0 | 0.0 | 0.0 | | 0.0 | 0.0 | 0.0 |
|  | 10% | 0.0 | 0.0 | 0.0 | | 0.0 | 0.0 | 0.1 |
| "Effect_of_incentive_on_trust_(change)" | -10% | 0.0 | 0.0 | 0.0 | | 0.0 | 0.0 | 0.0 |
|  | 10% | 0.0 | 0.0 | 0.0 | | 0.0 | 0.0 | 0.1 |
| Effect_of_supervision_on_knowledge[1, 1] | -10% | 0.0 | -0.1 | 0.0 | | 0.0 | 0.0 | 0.0 |
|  | 10% | 0.0 | 0.1 | 0.0 | | 0.0 | 0.0 | 0.0 |
| Hiring_rate | -10% | 0.0 | -0.1 | 0.0 | | 0.0 | 0.0 | 0.0 |
|  | 10% | 0.0 | 0.1 | 0.0 | | 0.0 | 0.0 | 0.0 |
| "Knowledge_of_health_workers_(IPT)"(t) | -10% | 0.3 | -0.3 | 0.2 | | 0.0 | 0.2 | 0.0 |
|  | 10% | -0.3 | 0.4 | 0.0 | | 0.0 | -0.2 | 0.0 |
| "Number_of_health_worker_at_health_facility_(%_filled)"(t) | -10% | 0.2 | -0.2 | 0.1 | | -0.1 | 0.0 | -0.1 |
|  | 10% | -0.2 | 0.3 | -0.1 | | 0.1 | 0.0 | 0.1 |
| Trust_in_programme(t) | -10% | 0.1 | -0.1 | 0.1 | | -0.2 | -0.2 | -0.1 |
|  | 10% | -0.1 | 0.1 | 0.0 | | 0.2 | 0.2 | 0.1 |

*Facility Funding sector*

Table S8.13: Sensitivity analyses for facility funding sector parameters, change in key outcomes.

|  |  | **Percentage of women who receive at least two doses of IPT during ANC** | | | **Percentage of women who seek facility-based delivery** | | |
| --- | --- | --- | --- | --- | --- | --- | --- |
| **Parameter values adjusted** | **Adjustment** | *Baseline* | *Endline* | *Longterm* | *Baseline* | *Endline* | *Longterm* |
| Alternative_facility_held_funding_available | -10% | 0.483 | 0.723 | 0.691 | 0.862 | 0.888 | 0.898 |
|  | 10% | 0.509 | 0.739 | 0.708 | 0.863 | 0.890 | 0.899 |
| "Target_to_receive_facility_incentive_(increase_in_%_points)" | -10% | 0.496 | 0.731 | 0.700 | 0.862 | 0.889 | 0.899 |
|  | 10% | 0.496 | 0.731 | 0.700 | 0.862 | 0.889 | 0.899 |
| "Target_to_receive_facility_incentive_(Overall_result)" | -10% | 0.496 | 0.731 | 0.693 | 0.862 | 0.889 | 0.898 |
|  | 10% | 0.496 | 0.727 | 0.700 | 0.862 | 0.888 | 0.899 |
| Use_of_incentives | -10% | 0.496 | 0.759 | 0.723 | 0.862 | 0.891 | 0.900 |
|  | 10% | 0.496 | 0.701 | 0.676 | 0.862 | 0.886 | 0.900 |

Table S8.14: Sensitivity analyses for facility funding sector parameters, change in key outcomes (% change).

|  |  | **Percentage of women who receive at least two doses of IPT during ANC** | | | **Percentage of women who seek facility-based delivery** | | |
| --- | --- | --- | --- | --- | --- | --- | --- |
| **Parameter values adjusted** | **Adjustment** | *Baseline* | *Endline* | *Longterm* | *Baseline* | *Endline* | *Longterm* |
| Alternative_facility_held_funding_available | -10% | -2.6 | -1.1 | -1.2 | -0.1 | -0.1 | -0.1 |
|  | 10% | 2.6 | 1.1 | 1.2 | 0.1 | 0.1 | 0.1 |
| "Target_to_receive_facility_incentive_(increase_in_%_points)" | -10% | 0.0 | 0.0 | 0.0 | 0.0 | 0.0 | 0.0 |
|  | 10% | 0.0 | 0.0 | 0.0 | 0.0 | 0.0 | 0.0 |
| "Target_to_receive_facility_incentive_(Overall_result)" | -10% | 0.0 | 0.0 | -1.0 | 0.0 | 0.0 | 0.0 |
|  | 10% | 0.0 | -0.5 | 0.0 | 0.0 | -0.1 | 0.0 |
| Use_of_incentives | -10% | 0.0 | 3.8 | 3.4 | 0.0 | 0.2 | 0.2 |
|  | 10% | 0.0 | -4.0 | -3.5 | 0.0 | -0.4 | 0.1 |

*District Manager Operations sector*

Table S8.15: Sensitivity analyses for district manager operations sector parameters, change in key outcomes.

|  |  | **Percentage of women who receive at least two doses of IPT during ANC** | | | **Percentage of women who seek facility-based delivery** | | |
| --- | --- | --- | --- | --- | --- | --- | --- |
| **Parameter values adjusted** | **Adjustment** | *Baseline* | *Endline* | *Longterm* | *Baseline* | *Endline* | *Longterm* |
| Baseline_motivation | -10% | 0.496 | 0.731 | 0.700 | 0.862 | 0.889 | 0.899 |
|  | 10% | 0.496 | 0.731 | 0.700 | 0.862 | 0.889 | 0.899 |
| "District_budget/resources" | -10% | 0.496 | 0.726 | 0.699 | 0.862 | 0.891 | 0.900 |
|  | 10% | 0.496 | 0.731 | 0.700 | 0.863 | 0.889 | 0.899 |
| District_manager_motivation_to_support_facilities(t) | -10% | 0.496 | 0.730 | 0.700 | 0.862 | 0.889 | 0.898 |
|  | 10% | 0.496 | 0.731 | 0.700 | 0.863 | 0.890 | 0.899 |
| Effect_of_incentive_amount_on_motivation | -10% | 0.496 | 0.731 | 0.700 | 0.862 | 0.889 | 0.899 |
|  | 10% | 0.496 | 0.731 | 0.700 | 0.862 | 0.889 | 0.899 |
| "Skill_level_or_knowledge_of_district_manager_(supervision)" | -10% | 0.496 | 0.730 | 0.700 | 0.862 | 0.889 | 0.898 |
|  | 10% | 0.496 | 0.731 | 0.700 | 0.863 | 0.890 | 0.899 |
| "Target_to_receive_CHMT_incentive_(Overall_result)" | -10% | 0.496 | 0.731 | 0.700 | 0.862 | 0.889 | 0.899 |
|  | 10% | 0.496 | 0.731 | 0.700 | 0.862 | 0.889 | 0.899 |

Table S8.16: Sensitivity analyses for district manager operations sector parameters, change in key outcomes (% change).

|  |  | **Percentage of women who receive at least two doses of IPT during ANC** | | | **Percentage of women who seek facility-based delivery** | | |
| --- | --- | --- | --- | --- | --- | --- | --- |
| **Parameter values adjusted** | **Adjustment** | *Baseline* | *Endline* | *Longterm* | *Baseline* | *Endline* | *Longterm* |
| Baseline_motivation | -10% | 0.0 | 0.0 | 0.0 | 0.0 | 0.0 | 0.0 |
|  | 10% | 0.0 | 0.0 | 0.0 | 0.0 | 0.0 | 0.0 |
| "District_budget/resources" | -10% | 0.0 | -0.6 | -0.1 | 0.0 | 0.2 | 0.1 |
|  | 10% | 0.0 | 0.0 | 0.0 | 0.0 | 0.0 | 0.1 |
| District_manager_motivation_to_support_facilities(t) | -10% | 0.0 | 0.0 | 0.0 | 0.0 | 0.0 | 0.0 |
|  | 10% | 0.0 | 0.0 | 0.0 | 0.0 | 0.0 | 0.1 |
| Effect_of_incentive_amount_on_motivation | -10% | 0.0 | 0.0 | 0.0 | 0.0 | 0.0 | 0.0 |
|  | 10% | 0.0 | 0.0 | 0.1 | 0.0 | 0.0 | 0.0 |
| "Skill_level_or_knowledge_of_district_manager_(supervision)" | -10% | 0.0 | -0.1 | 0.0 | -0.1 | 0.0 | -0.1 |
|  | 10% | 0.0 | 0.1 | 0.0 | 0.1 | 0.0 | 0.1 |
| "Target_to_receive_CHMT_incentive_(Overall_result)" | -10% | 0.0 | 0.0 | 0.0 | 0.0 | 0.0 | 0.0 |
|  | 10% | 0.0 | 0.0 | 0.0 | 0.0 | 0.0 | 0.0 |

References

Binyaruka P, Patouillard E, Powell-Jackson T *et al.* 2015. Effect of Paying for Performance on Utilisation, Quality, and User Costs of Health Services in Tanzania: A Controlled Before and After Study. Ostermann J (ed.). *PLoS One* **10**: e0135013.

Borghi J, Binyaruka P, Mayumana I *et al.* 2021. Long-term effects of payment for performance on maternal and child health outcomes: evidence from Tanzania. *BMJ Glob Heal* **6**: e006409.

Borghi J, Mayumana I, Mashasi I *et al.* 2013. Protocol for the evaluation of a pay for performance programme in Pwani region in Tanzania: A controlled before and after study. *Implement Sci* **8**: 80.

Cassidy R, Borghi J, Semwanga AR *et al.* 2022. How to do (or not to do)…using causal loop diagrams for health system research in low and middle-income settings. Health Policy Plan, DOI: 10.1093/heapol/czac064.

Cassidy R, Tomoaia-Cotisel A, Semwanga AR *et al.* 2021. Understanding the maternal and child health system response to payment for performance in Tanzania using a causal loop diagram approach. *Soc Sci Med* **285**: 114277.

Ensor T, Quigley P, Green C *et al.* 2014. Knowledgeable antenatal care as a pathway to skilled delivery: modelling the interactions between use of services and knowledge in Zambia. *Health Policy Plan* **29**: 580–8.

isee systems inc. 2021. STELLA Architect.

Kurowski C, Wyss K, Abdulla S *et al.* 2007. Scaling up priority health interventions in Tanzania: the human resources challenge. *Health Policy Plan* **22**: 113–27.

Ministry of Health and Social Welfare. 2012. *The Pwani Region Pay-for-Performance (P4P) Pilot Design Document*. Dar es Salaam: MoHSW, United Republic of Tanzania

Ministry of Health and Social Welfare. 2013. *Human Resource for Health Country Profile 2012/2013*. p 34. Date of access: December 29, 2022. URL: https://www.jica.go.jp/project/tanzania/006/materials/ku57pq00001x6jyl-att/country_profile_2013.pdf

Ministry of Health and Social Welfare. 2014a. *Staffing Levels for Ministry of Health and Social Welfare Departments, Health Service Facilities, Health Training Institutions and Agencies 2014-2019.* p xi. Date of access: December 29, 2022. URL: https://www.jica.go.jp/project/tanzania/006/materials/ku57pq00001x6jyl-att/REVIEW_STAFFING_LEVEL_2014-01.pdf

Ministry of Health and Social Welfare. 2014b. *Human Resource for Health and Social Welfare Country Profile 2013/2014*. p 35. Date of access: December 29, 2022. URL: https://www.jica.go.jp/project/tanzania/006/materials/ku57pq00001x6jyl-att/COUNTRY_PROFILE.pdf

National Bureau of Statistics. 2011. *Tanzania Demographic and Health Survey*. p 55, 118, 263. Date of access: December 29, 2022. URL: https://dhsprogram.com/publications/publication-fr243-dhs-final-reports.cfm

National Bureau of Statistics. 2013. *Population and Housing Census: Population Distribution by Age and Sex*. p 157. Date of access: December 29, 2022. URL: https://www.nbs.go.tz/index.php/en/census-surveys/population-and-housing-census/163-phc-2012-population-distribution-by-age-and-sex-report

National Bureau of Statistics. 2015. *Mortality and Health Report*. p 24-26.

National Bureau of Statistics. 2018. *National Population Projections*. p 67-76. Date of access: December 29, 2022. URL: https://www.nbs.go.tz/nbs/takwimu/census2012/Projection-Report-20132035.pdf

Pruyt E. 2017. System dynamics: a tool for modelling and testing solutions. In: de Savigny D, Blanchet K, Adam T. Applied Systems Thinking for Health Systems Research : A Methodological Handbook. McGraw-Hill Education, 173–94.

Semwanga AR, Nakubulwa S, Adam T. 2016. Applying a system dynamics modelling approach to explore policy options for improving neonatal health in Uganda. *Heal Res Policy Syst* **14**: 35.

UNESCO Institute for Statistics. United Republic of Tanzania: Education and Literacy. Date of access: July 20, 2021. URL: http://uis.unesco.org/en/country/tz
